# Synthesis and In Vitro Profiling of Psilocin Derivatives: Improved Stability and Synthetic Properties

**DOI:** 10.1021/acs.jmedchem.4c02612

**Published:** 2025-03-20

**Authors:** Julia Eklund, Ulf Bremberg, Jessica Larsson, Edvard Torkelsson, Johan Wennerberg, Symantha Zandelin, Luke R. Odell

**Affiliations:** 1 Department of Medicinal Chemistry, Uppsala University, Box-574, Uppsala SE-751 23, Sweden; 2 Red Glead Discovery, Medicon Village, Lund SE-223 81, Sweden; § Department of Chemistry, Lund University, Lund SE-221 00, Sweden

## Abstract

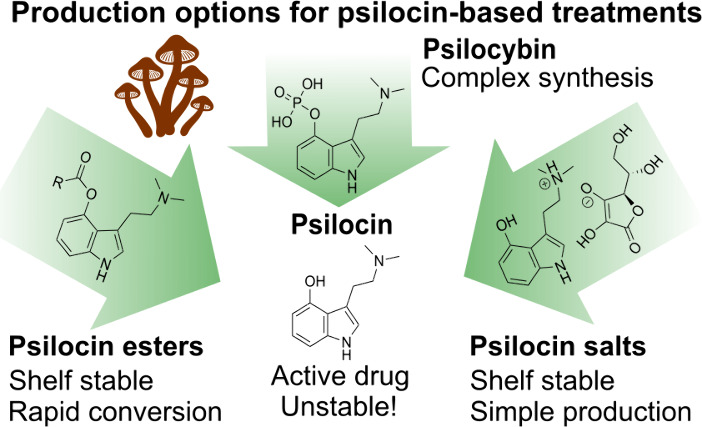

As interest in using psilocybin therapy for treating mental health disorders intensifies, the need for efficient production methods becomes increasingly important. Current medical-grade psilocybin production is inefficient and relies on a complicated multistep synthesis. This study has explored and evaluated psilocin ester prodrugs and psilocin salts as potential alternatives to psilocybin, focusing on their ease of synthesis, chemical stability, and metabolic profiles. A diverse library of 15 psilocin ester prodrugs and six psilocin salts was synthesized and evaluated. The study successfully identified several psilocin ester prodrugs and psilocin salts that exhibited desirable characteristics, including storage and handling stability, rapid metabolic conversion to psilocin, and easy synthesis, with potential advantages over psilocybin. This research introduces viable options through psilocin ester compounds and psilocin salts, offering promising avenues for future development.

## Introduction

In recent years, the therapeutic potential of psilocybin (4-phosphoryloxy*-N,N*-dimethyltryptamine) therapy for treating various mental health disorders has captured increasing interest. This surge in attention has led to a heightened demand for medical-grade psilocybin (suitable for GMP production), underscoring the need for efficient and scalable production methods.^1,2^ The most predominant method for the synthetic production of psilocybin today is a process described by Nichols and Frescas^3^ and optimized by Sherwood et al.^4^ and others.^5−9^ While this route was an improvement of the original method published by Hofmann,^10^ it has some limitations. The major drawback of this approach is the final step focused on the phosphorylation of psilocin (4-hydroxy-*N,N*-dimethyltryptamine), which is complicated and low yielding (47%).^3^ In 2020, Kargbo et al. published a greatly simplified kilogram-scale synthesis of psilocybin using POCl_3_ to phosphorylate psilocin;^11^ however, despite our best efforts we were unable to reproduce this synthesis, even after consulting the authors. While synthetic methods dominate, alternative production options exist, such as extracting psilocybin directly from cultivated mushrooms. Unfortunately, the feasibility of this natural extraction method at an industrial scale may be limited by relatively low yields and complex isolation/purification. Another innovative approach is the bioengineering of psilocybin in e.g., *E. coli*,^12^ which proposes a cost reduction by utilizing less expensive starting materials.^13^ Despite its potential, this method is currently limited to gram-scale experiments, and extensive research is required to enhance yields and achieve commercial viability.^2^

Upon ingestion, the prodrug psilocybin is rapidly metabolized into the active substance psilocin.^14^ This conversion is verified through serotonin 5-HT receptor binding studies, highlighting psilocybin’s low receptor affinity and role as a prodrug of the active compound, psilocin.^15^ In the quest for alternatives to psilocybin, synthetic prodrugs of psilocin such as 4-AcO–DMT (psilacetin, 4-acetoxy-*N,N*-dimethyltryptamine) have been formulated, patented by Albert Hofmann and Franz Troxler in 1963.^16^ In 1999, David Nichols introduced the fumarate salt of 4-AcO–DMT, labeling it the “O-Acetyl Prodrug of Psilocin” and suggested that psilacetin might function as a prodrug of psilocin.^3^ However, recent studies have shown that psilacetin demonstrates significant serotonergic receptor activities in its own right in cellular assays, in addition to being a prodrug for psilocin.^17,18^ Although it is very likely that psilacetin undergoes conversion to psilocin in vivo, information on how rapidly this occurs remains lacking. As a result, it remains unclear whether psilacetin itself can directly engage serotonergic receptors before extensive metabolic conversion. Other acetyl-substituted tryptamines, such as 4-AcO–DET, 4-AcO–DiPT, and 4-AcO–DALT, have also been explored for their pharmacological profiles, further highlighting the interest in this class of compounds.^18−21^ These efforts underscore the importance of investigating novel prodrug alternatives to expand therapeutic options. Indeed, a recent study by Raithatha and co-workers confirmed that N- and O-modified psilocin analogs have unique pharmacological profiles that can differ markedly from that of psilocybin.^22^

Another feasible approach could involve employing the active metabolite psilocin directly in treatments. However, psilocin’s inherent instability poses significant challenges, as the compound degrades during storage. If a more stable form of psilocin could be developed, it could significantly simplify production processes. In fact, a clinical study conducted by Beckley Psytech has already explored this possibility, utilizing psilocin benzoate.^23^ Nevertheless, comprehensive information on the stability of various psilocin salts and formulations remains scarce.

Given these challenges, our study has explored two alternative strategies aimed at improved access to psilocybin-based therapies: the development of psilocin ester prodrugs and the use of psilocin salts. By exploring these alternatives, we aim to identify solutions to address production and stability challenges, making psychedelic treatment and research more accessible.

## Results and Discussion

### Psilocin Esters

Our aim was to synthesize a variety of esters characterized by substantial structural diversity, aiming to encompass a wide spectrum of chemical and metabolic stabilities, exploring the balance between stability during handling and storage, and rapid conversion into psilocin upon ingestion.

One of our choices in ester synthesis was carboxylic acid esters, as one of the most prevalent types of prodrugs. These esters are known for their innate susceptibility to hydrolysis, releasing the active drug compound. The hydrolysis of carboxylic acid esters is primarily facilitated by esterase enzymes, which are abundantly distributed in saliva, plasma and various tissues.^24,25^ In our pursuit of a diverse range of stabilities, we extended our synthesis efforts to include a selection of carbonate esters, alongside a single carbamate ester, to explore a greater range of resistance to enzymatic hydrolysis.^26^ In addition, we synthesized a borate ester^27,28^ and a sulfate ester,^29^ expected to be metabolized by sulfatases and other hydrolyzing enzymes. The borate ester **5** and the sulfate ester **6**, were prepared adapting previously reported procedures.^30,31^ The phosphate ester psilocybin was synthesized as a reference compound. Phosphate esters are frequently employed in prodrug development due to their numerous benefits including high water solubility and cellular permeability, as well as considerable metabolic lability, which can be attributed to the widespread distribution of phosphatases in the body.^32^ Psilocybin **8**, was prepared using a previously published procedure.^4^

### Synthesis

**Scheme 1:**
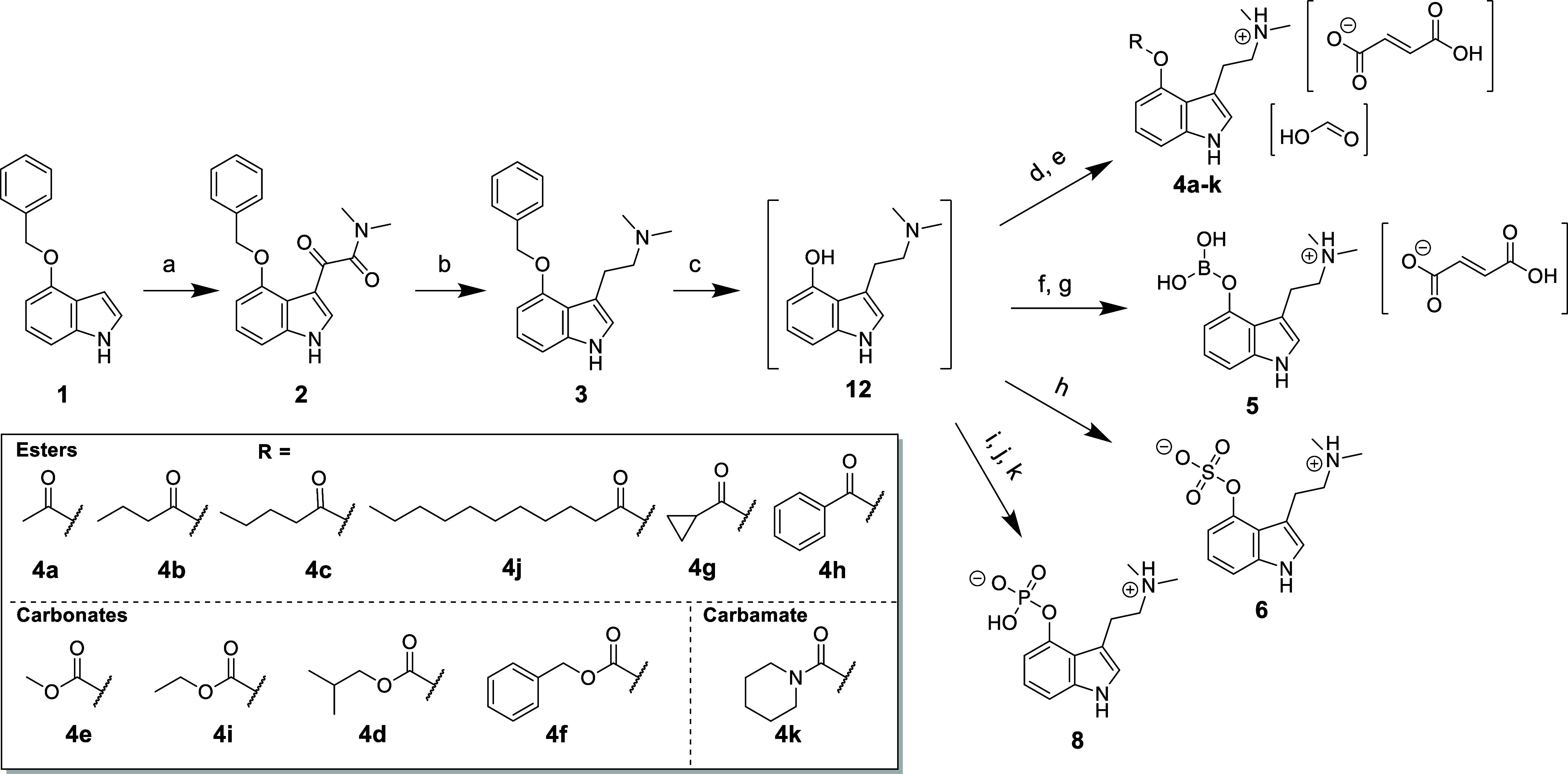
Synthesis of Target Compounds **4–8** Reagents and conditions (a) (i) (COCl)_2_, Et_2_O, 0 °C for 15 min, then rt for 3 h; (ii) (CH_3_)_2_NH, 0–20 °C, 3 h, 78%; (b) LiAlH_4_, 2-MeTHF, reflux, 8 h, 84%; (c) H_2_, Pd/C, toluene/THF, rt, 6 h; (d) RCO_2_O/RCOCl, Cs_2_CO_3_, toluene, rt, 24 h; (e) fumaric acid, EtOH, 30 min, 1–31%; (f) B(OH)_3_, toluene, 80 °C, 3 h; (g) fumaric acid, EtOH, 30 min, 16%; (h) SO_3_•pyridine complex, pyridine, THF, 45 °C, 2 h, 22%; (i) nBuLi, TBPP, THF, −78 – −20 °C, 1.5 h; (j) DCM, reflux, 5 min, 38%; (k) H_2_, Pd/C, MeOH, rt, 1 h, 39%.

Late-stage diversification strategy based on the one-pot deprotection/functionalization of O-benzyl psilocin **3**, (Scheme 1) was employed to effectively synthesize a set of psilocin prodrugs. Notably, this approach would eliminate the need for isolation of air- and light-sensitive psilocin^33^ allowing straightforward purification of the target prodrugs **4**–**6** and **8** following esterification, borylation, sulfonation or phosphorylation.

The synthesis of **3** was achieved using the method described by Nichols,^3^ although a solution of dimethylamine (THF) was used instead of gaseous dimethylamine, making the procedure more convenient on a lab scale. Fortunately, this also resulted in precipitation of **2** from the reaction mixture affording the dimethylamide derivative in 78% yield without any need for further purification. Reduction of **2** with LiAlH_4_ in refluxing 2-MeTHF gave **3** in 84% yield after recrystallization.

**Figure 1:**
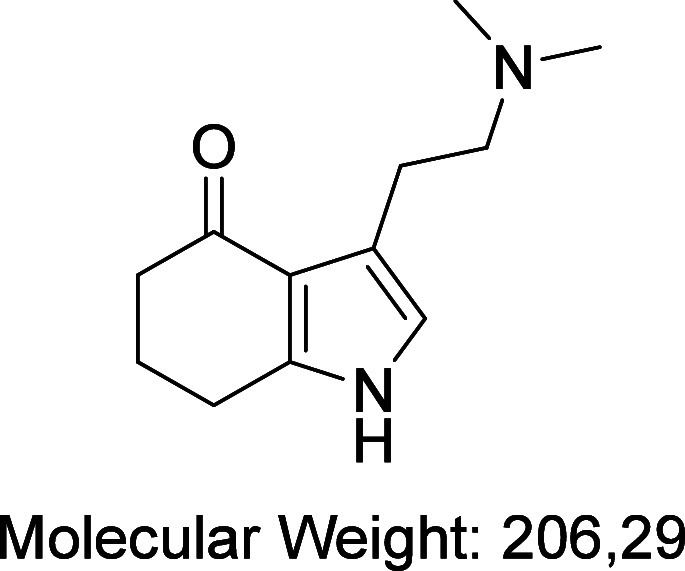
Side product with *m*/*z* 207.

Our initial synthesis attempts focused on the *in situ* deprotection/esterification of **3** using an array of anhydrides and acid chlorides. This resulted in poor conversions, most likely due competing Pd-catalyzed reduction of the activated carboxylic acid derivatives. We also noted formation of significant amounts of a side-product with an *m*/*z* of 207 (Figure 1) that we suspected was due to partial reduction of psilocin.^34^

After some optimization, we noted that this could be minimized by using certain solvents (THF or toluene) and limiting the hydrogenation time to 6 h. To minimize decomposition of the sensitive psilocin intermediate, the catalyst was removed under nitrogen and the psilocin solution was added directly to a solution of the appropriate electrophile. Here it was noted that the choice and amount of base was crucial to avoid competing indole *N*-acylation and the best results were obtained using a small excess of Cs_2_CO_3_. The ester-derived prodrugs **4a**–**k** (Scheme 1) were initially purified using preparative chromatography. Due to their oily nature, these compounds were subsequently precipitated as fumarate salts to facilitate easier handling. However, the yields of the resulting salts were generally low to modest, and this was primarily attributed to losses incurred during the precipitation process, which were compounded by the variable solubility of the different salts (yields before salt conversion are shown in Table S9). The borate **5**, sulfonate **6**, and phosphate **8**, (psilocybin) esters were prepared in a similar fashion following treatment with the appropriate electrophile (Scheme 1).^4,30,31^

### Evaluation of Chemical Stability

The stability of the synthesized compounds was assessed under various pH conditions by subjecting them to 1.0 M HCl, 0.10 M HCl (simulated stomach acid) and 1.0 M NaHCO_3_ to better understand their shelf life and handling requirements (Table 1). It would be most beneficial if a prodrug was acid-sensitive, swiftly hydrolyzing in gastric acid, yet maintaining stability during manufacture, handling and storage. However, stability in acidic conditions may not present a significant issue as esterases could initiate hydrolysis at a later stage. With the exception of compound **5**, all the synthesized compounds, including psilocybin, demonstrated stability in simulated stomach acid (0.1 M HCl). Compounds **8** (psilocybin), **3**, **6** & **4k**, also demonstrated stability in both acidic and basic concentrations (1 M HCl and 1 M NaHCO_3_).

**Table 1:** Evaluation of Chemical Stability^a^

	**(min)**		
compound	1.0 M NaHCO_**3**_	1.0 M HCl	0.10 M HCl
**3**	>10,000	>10,000	>10,000
**4a**	900	300	>10,000
**4b**	3900	900	>10,000
**4c**	3000	800	>10,000
**4d**	2200	>10,000	>10,000
**4e**	1000	9000	>10,000
**4f**	700	6000	>10,000
**4g**	11,000	6000	>10,000
**4h**	9000	>10,000	>10,000
**4i**	3000	>10,000	>10,000
**4j**	1000	1300	>10,000
**4k**	>10,000	>10,000	>10,000
**5**	<5 min	<5 min	<5 min
**6**	>10,000	>10,000	>10,000
**8**	15,000	>10,000	>10,000

a Performed at rt over 24h.

### Evaluation of Metabolic Stability

Due to the very low metabolic stability, many of the compounds were run multiple times with comparable results (Table 2, Table S3). The majority of compounds were found to provide unexpected low response in LC-MS/MS even at the first time point. All compounds gave the expected signals using the same LC-MS/MS method in control samples using only buffer, hence the very low obtained peak areas are due to the addition of biological material. Therefore, additional experiments comparing stability with and without the addition of NADPH cofactor and a very short incubation period were performed (Tables S4 and S5). The results are consistent with very rapid degradation of prodrugs to psilocin by esterases or other non-NADPH-dependent enzymes in the microsomal fraction for some of the prodrugs.^35^ Due to the rapid observed degradation in microsomes, we decided to investigate the stability of five compounds in human plasma, aiming for a broad range of stabilities (Table 2, Table S6). It should be noted that esterase-labile compounds are notoriously difficult to study in microsomal stability studies.^36^

**Table 2:**
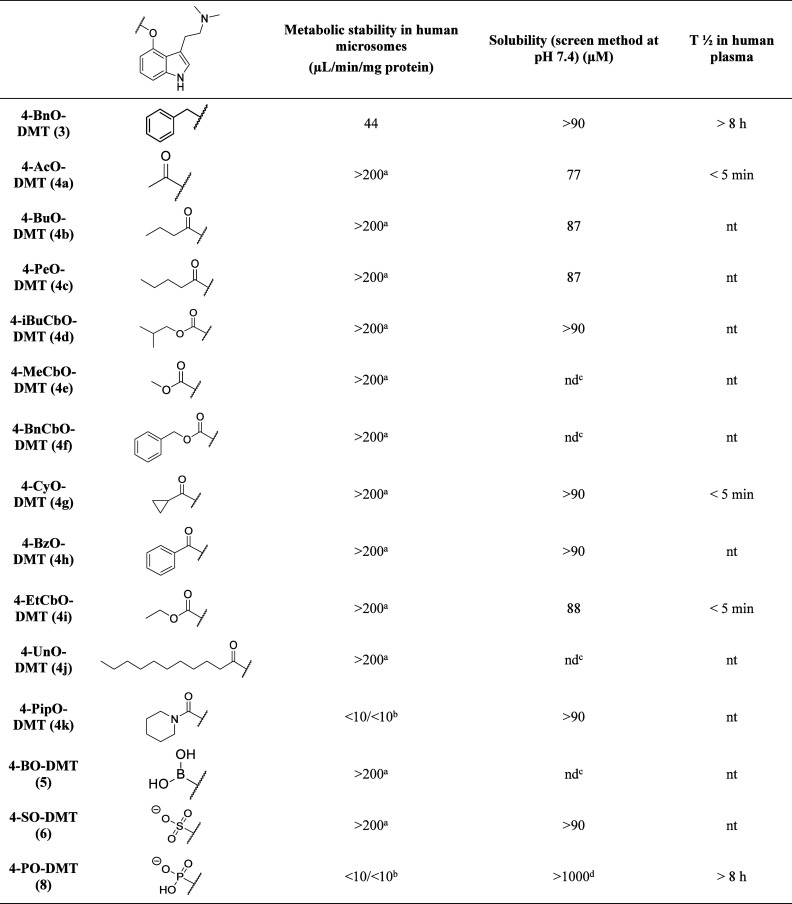
Summary of Metabolic Stability Assessments

a Very low peak area for first time point, instability in assay conditions suspected and confirmed.

b Weak retention in chromatography.

c Solubility could not be accurately determined due to degradation, but the observed data indicate solubility >90 μM.

d Results obtained with solid solubility assay as the sample coeluted with DMSO. nd = not determined, nt = not tested.

### Plasma Stability

For compounds **4a**, **4g** and **4i**, almost no compound remained after the sampling at 5 min, while no degradation was observed for compound **3** or the reference compound **8** (psilocybin). An additional experiment where the compounds were added to plasma pretreated with acetonitrile to precipitate enzymes (Tables S6 and S7) revealed that **4a** and **4i** were also heavily degraded in inactivated plasma while **3, 4g** and **8** were fairly stable. This suggest that very low levels of esterases are sufficient for degradation of the labile acetyl ester and ethyl carbonate groups.

### Degradation of Psilocin Ester Prodrugs in Simulated Gastrointestinal Conditions

Given that the literature has shown that psilacetin and several other psilocin ester prodrugs possess affinity for serotonin receptors, our objective was to ensure that these prodrugs are fully converted into psilocin before reaching systemic circulation. Rapid conversion in the stomach would render the receptor affinity of the intact prodrug irrelevant. To address this concern, we investigated the stability of the ester prodrugs in Fasted State Simulated Intestinal Fluid (FaSSIF) supplemented with porcine liver esterase (20 IU/mL).

The half-lives of six psilocin ester prodrugs were determined through triplicate measurements. Although all prodrugs degraded rapidly, there was significant variability in their degradation rates (Table 3). Prodrug **4d** exhibited the fastest degradation, while the other prodrugs showed progressively longer half-lives, indicating differences in their stability under the simulated gastrointestinal conditions.

**Table 3:** Degradation Kinetics of Psilocin Esters in Simulated Gastrointestinal Conditions

				**% prodrug remaining**	
compound	**(min)**^a^	**predicted****(min)**^b^	**predicted abs.**^d^	**no esterase30 min.**^e^	**measured after1 min.**^c^
**4a**	0.91 ± 0.10	0.07	6.2 × 10^–21^		47
**4d**	0.26 ± 0.02	0.02	7.0 × 10^–77^		7
**4e**	1.41 ± 0.32	0.10	4.1 × 10^–13^	100	60
**4f**	0.44 ± 0.04	0.03	6.6 × 10^–45^		21
**4g**	1.22 ± 0.04	0.09	1.9 × 10^–15^	100	57
**4i**	0.56 ± 0.12	0.04	2.6 × 10^–35^	100	28

a At 20 IU/mL (Mean ± SD).

b At 270 IU/mL.

c After 1 min at 20 IU/mL.

d Calculated remaining prodrug available for GI absorption, predicted level after 5 min at 270 IU/mL.

e % prodrug remaining after 30 min with no esterase.

To extrapolate these findings to physiological conditions, an esterase activity of 270 IU/mL, representing the lower limit observed in the fasted state,^37^ was assumed. Given that enzyme-catalyzed reactions generally follow first-order kinetics, the half-lives and % remaining prodrug after 5 min at 270 IU were recalculated accordingly. All prodrugs exhibited a rapid degradation to psilocin under these conditions, with negligible amounts calculated to remain (Table 3).

### Evaluation of Solubility

For a drug to be orally available, it normally must be soluble in an aqueous medium. The solubility data for the compounds were determined in a screening method with phosphate buffer pH 7.4 including 1% DMSO and is presented in Table 2 (Table S8). The more labile compounds degraded over time in DMSO, causing challenges for the solubility determinations. This could be solved by freshly made DMSO solutions, and in one case (**8**) by using solids for solubility determination. The obtained data suggest high solubility (>85 μM) for most compounds. In this assay, the solubility range is limited from 0.1 to 100 μM.

### Discussion

Our aim was to identify easily synthesized, chemically stable and metabolically labile psilocin ester prodrugs as potential bioequivalent alternatives to psilocybin. The goal was to synthesize psilocin esters covering a broad range of metabolic stabilities. Fifteen compounds were synthesized, and the chemical and metabolic stability was evaluated.

Several of the compounds, specifically **4d**, **4h** & **4i** demonstrated desirable properties for a psilocin ester prodrug. These compounds, warrant further examination to determine their viability as psilocybin substitutes. Although not all esters were tested for degradation in plasma, it is assumed that their degradation patterns will be similar to those observed in microsomes due to the presence of esterases in both media.

Ultimately, all the assessed compounds underwent degradation, except for the ether **3**, phosphate **8**, and carbamate **4k**. These results are consistent with previous findings where psilocin carbonate prodrugs were shown to degrade under acidic conditions and convert to psilocin upon oral administration.^38^

An unexpected result was the observed stability of psilocybin in both plasma and microsomes, despite the fact that psilocybin degrades rapidly upon ingestion.^1,14,32^ This discrepancy in degradation rates could be attributed to the presence of phosphatases, which might be more abundant in other organs than the liver and blood. This is consistent with the publication by Eivindvik et al., which indicates that the site of conversion for psilocybin in rats occurs in the jejunum.^39^ Understanding the nuances of *in vivo* psilocybin hydrolysis could provide valuable insights for future investigations in this field.

However, the situation is complex when we consider prodrugs such as the psilocin esters. The key lies in the location and timing of their conversion to psilocin. If the conversion happens before entering the systemic circulation, the properties of the prodrugs are less important, as it is the properties of psilocin that will dictate its absorption and distribution. The ideal prodrug should possess chemical stability across a wide pH range, high aqueous solubility, and good permeability, all of which contribute to its effective delivery within the body. Additionally, it is critical for the prodrug to have little or no pharmacological effects or toxicity on its own. Furthermore, once the prodrug is ingested, it should undergo a rapid and quantitative breakdown to psilocin. It is important to note that the activation of a prodrug can occur at different stages in the body - prior to absorption, either in the oral cavity, gastrointestinal fluid or the intestinal enterocytes, or postabsorption, in plasma, the liver or other compartments. Finally, the toxicity of the byproduct from activating the prodrug should be considered.

Our stability testing in FaSSIF supplemented with esterase indicates that psilocin ester prodrugs, including 4-AcO–DMT, are rapidly converted to psilocin under simulated gastrointestinal conditions. When extrapolated to physiological esterase activity (270 IU/mL), all tested prodrugs degrade rapidly enough to remain well below the 0.1% impurity threshold within 5 min in the stomach, minimizing the potential for prodrugs interacting with off-targets. The reduction of prodrug contents to <0.1% is relevant since this is the regulatory limit for impurity contents, avoiding extensive toxicology investigations. Discussions with regulators will be required to support this interpretation of prodrugs as a psilocin formulation, leaving <0.1% prodrug as impurity. This rapid conversion supports the intended use of these prodrugs as alternatives to psilocybin, ensuring that only psilocin is efficiently delivered to the bloodstream. These results are consistent with those of Raithatha et al., who also reported rapid metabolism of their ester prodrugs to psilocin across all biological fractions tested^22^ and a recently published study reported a nonsignificant difference in psilocin exposure after dosing mice with psilocybin or 4-AcO–DMT.^40^ Plasma measurements of psilocin release from a prodrugs represent a conservative estimate of esterase activity, since whole blood and other tissues in the body are known to have significantly higher esterase activity.^41^

The variability in half-lives among the prodrugs indicates that some, such as **4d**, are more rapidly converted than others. This rapid conversion aligns with our objective to eliminate the receptor activity of the intact ester and suggests that prodrugs like **4d** could offer favorable pharmacokinetic profiles. The consistent esterase activity levels between fasted and fed states,^37^ indicate that the findings should be generalizable to most patients. The observed difference in stability is consistent with published esterase reactivity of esters and carbonates.^42^

Our results support the hypothesis that ester prodrugs can serve as effective alternatives to psilocybin, providing predictable pharmacokinetics and reduced risk of unintended pharmacological effects due to intact prodrug presence in the systemic circulation. Further studies are needed to confirm these results in vivo and to explore the pharmacokinetic profiles of these ester prodrugs compared to psilocybin, particularly focusing on their conversion rates and potential to achieve therapeutic effects with minimal side effects.

Finally, the observation of high solubility of the psilocin esters is an encouraging outcome, indicating the possibility of high oral bioavailability, although further in vitro and in vivo tests are needed to confirm this.

### Psilocin Salts

Next, we assessed the stability of various psilocin salts, aiming at simplifying manufacturing processes and improving handling and storage of psilocin-based drug products. Psilocin salts were subjected to acid and base treatment, thermal degradation, photolysis, and oxidation to evaluate their stability (Table 4). Stability assessments were conducted in a deionized water:acetonitrile solution (1:1).

**Table 4:** Forced Degradation Study Design

**stress condition**	**conditions**	**sampling time**
acid	1.0 M HCl, 20 °C	1, 3, and 23 h
base	0.10 M NaOH, 20 °C	1, 3, and 23 h
oxidative	0.30% H_2_0_2_, 20 °C	1, 3, and 23 h
thermal	water bath, 60 °C	5, 24, and 143 h
photolytic	UV lamp, 20 °C	1, 7, and 15 days

Several salts (Table 5) were chosen for evaluation of psilocin stability. HCl was selected for its prevalence in the literature, psilocin benzoate for its reported stability in IV infusions,^23,43^ and ascorbic and boric acid for their antioxidant properties.^44^ Trifluoroacetic acid (TFA) was targeted for its higher acidity and weaker counterion effects, and phosphoric acid for its common use in pharmaceutical salts^32^ and similarity to psilocybin.

**Table 5:** General Observations of Psilocin Salts in Solid Form Over 3 Weeks in −18 °C Freezer

compound	**salt**	**appearance**	**stability**^a^	**hygroscopic**^b^
**9a**	HCl	white crystals	not very stable	yes
**9b**	borate	white powder	no change	no
**9c**	benzoate	white powder	no change	no
**9d**	ascorbate	yellow powder	no change	no
**9e**	phosphate	white powder	darkens after 1 week	no
**9f**	TFA	light brown crystals	degrades within 3 days	yes

a Assessed by visual inspection and LCMS analysis.

b Assessed by visual inspection.

### Sample Preparation and Storage

The salt preparation was performed under inert atmosphere by adding the appropriate acid dissolved in isopropanol, to a solution of psilocin free base in THF at room temperature. The solution was stirred at room temperature for 30 min before the solvent was evaporated.

Psilocin salt samples were stored under nitrogen wrapped in aluminum foil at −18 °C to minimize exposure to light and oxidative degradation. During the handling of the psilocin salts, specific properties were noted which may impact their suitability for pharmaceutical use (Table 5). Both the HCl salt **9a** and the TFA **9f** salt demonstrated highly hygroscopic properties, visual inspections noting a transition from a crystalline form to an oily appearance. This characteristic is generally considered undesirable for pharmaceutical salts, as it can affect the stability, storage, and handling of the product.^45^ Additionally, the TFA salt **9a** displayed significant stability issues, with the first batch degrading during storage after only 3 days, necessitating the preparation of a new batch for analysis.

During this period, the salt changed color to black, and its degradation was confirmed by LCMS. Conversely, the borate **9b**, benzoate **9c** and ascorbate **9d** appeared to be stable enough to store as a solid in the freezer for an extended period (Table 5).

### Stability of Psilocin Salts in Acidic Conditions (1.0 M HCl)

All tested psilocin salts demonstrated high stability when subjected to 1.0 M HCl, with no observable degradation by LCMS over 24 h. As no decrease of psilocin could be observed, the half-life is expected to exceed 10 000 min (1 week) (Table 6, Figure S1). These results are consistent with previous findings, showing that psilocin is stable in acidic environments for 24 h.^46^

**Table 6:** Forced Degradation Studies of Psilocin Salts in Solution After 24 h at RT

		1.0 M HCl		0.10 M NaOH			**0.30% H**_**2**_**O**_**2**_			
compound	**salt**	**color**^a^	**% psilocin remaining**^b^	**(min)**	**color**	**% psilocin remaining**	**(min)**	**color**	**% psilocin remaining**	**(min)**
**9a**	HCl	clear	100%	>10,000	black	0%	500	pink tint	100%	>10,000
**9b**	borate	clear	93%	>10,000	black	0%	400	brown	0%	<60
**9c**	benzoate	clear	100%	>10,000	black	0%	500	brown tint	94%	>10,000
**9d**	ascorbate	clear	97%	>10,000	black	6%	1000	yellow tint	98%^d^	>10,000^d^
**9e**	phosphate	clear	94%	>10,000	black	0%	300	gray	83%	800^c^
**9f**	TFA	clear	99%	>10,000	black	0%	900	yellow	78%	2000

a Assessed by visual inspection after 24h.

b % Psilocin remaining after 24 h compared with the first measuring point at 1 h, measured with LCMS.

c Underestimated due to insufficient mixing in one sample.

d Mean value from two separate runs performed in triplicates.

### Stability of Psilocin Salts under Basic Conditions (0.10 M NaOH)

When subjected to basic conditions using an excess of NaOH, the ascorbic salt **9d** demonstrated superior resistance to degradation compared to other tested salts. It was the only salt to retain a small, yet detectable quantity of psilocin after 24 h. In contrast, all other salts were completely degraded after 24 h. (Table 6, Figure S2). Barrow et al. have reported a 60% degradation of psilocin freebase after only 2 h in NaOH solution (1 M).^46^

### Response to Oxidative Stress (0.30% H_2_O_2_)

When exposed to 0.30% H_2_O_2_, the ascorbate **9d,** benzoate **9c** and HCl salts **9a** displayed exceptional stability, with no degradation observed and a calculated half-lives exceeding 10000 min (1 week). The phosphate **9e**, and TFA **9f** salts exhibited moderate stability, each showing only a slight reduction in psilocin concentration, with their half-lives more than 24 h. The borate salt **9b**, however, was notably less stable, undergoing complete degradation within just 3 h of exposure (Table 6, Figure S3).

### Thermal Stability at 60 °C

Thermal exposure (in water:acetonitrile solution, 1:1) highlighted the remarkable stability of the ascorbic salt **9d**, which retained about 42% after 143 h (6 days), making it the only salt with detectable psilocin levels at the end of this period, as all other salts had completely degraded (Figures S4, S8, and S16). The calculated half-life for ascorbate was 9000 min, nearly 4 days and the phosphoric salt had a half-life of 4000 min. In contrast, the HCl **9a**, benzoic **9c**, and TFA **9f** salts all had a half-life of less than 2 days, while the borate salt **9b** was very unstable, completely degrading by the 24 h mark (Table 7, Figure S4).

**Table 7:** Thermal Degradation Studies of Psilocin Salts in 60 °C After 6 Days

compound	**salt**	**color(24 h)**^a^	**% psilocin remaining**	**(min)**
**9a**	HCl	black	0%	3000
**9b**	borate	black	0%	<700
**9c**	benzoate	black	0%	2000
**9d**	ascorbate	golden brown	42%^b^	9000^b^
**9e**	phosphate	black	0%	4000
**9f**	TFA	black	0%	2000

a Pictures of the samples were taken after 24h.

b Mean value of triplicates.

### Photolytic Stability

Photolytic stability assessments at RT in water:acetonitrile, 1:1 (12900 lx combined 465 nm and broad spectrum), demonstrated that the ascorbic **9d** and HCl **9a** salts were highly resistant to light-induced degradation, retaining 39% and 51% of their psilocin content respectively after 14 days. In contrast, the phosphoric **9e** and TFA **9f** salts were less stable, with only 33% and 22% of psilocin remaining after the same period. The benzoate **9c** and borate **9b** salts underwent rapid degradation, completely breaking down within 1 week. Notably, further analysis of a control sample, which was stored in darkness in air at RT for a week, showed that the dissolved borate salt **9b** was entirely degraded, suggesting that factors beyond light exposure contributed to its rapid breakdown (Table 8, Figure S5). In related studies, when exposed to UV radiation at 254 nm, psilocin freebase in EtOH was found to be notably unstable, showing 9% degradation after just 1.75 h.^46^

**Table 8:** Photolytic Degradation Studies of Psilocin Salts After 14 Days

compound	**salt**	**color(24 h)**^a^	**% psilocin remaining**	**(min)**
**9a**	HCl	gray	51%	20,000
**9b**	borate	black	0%	<1000^b^
**9c**	benzoate	brown	0%	9000
**9d**	ascorbate	yellow tint	39%^c^	20,000^c^
**9e**	phosphate	gray	33%	20,000
**9f**	TFA	black	22%	10,000

a See Figure S13.

b Analysis of the control sample, not exposed to light, revealed complete degradation of the borate salt, indicating degradation was independent of light exposure.

c Mean value of triplicates.

### Visual Inspection

In acidic and basic environments, discoloration was a clear indicator of degradation, with some salts showing greater resistance than others. Oxidative and thermal conditions also revealed significant differences in salt stability through changes in color, ranging from slight discoloration to complete darkening. Photolytic testing confirmed these patterns, where lesser color changes suggested higher stability. Visual assessments aligned well with LCMS purity so that changes in color indicated increased levels of psilocin impurities (Table 6, Figures S6–S13).

### Discussion

While numerous researchers have explored production methods of psilocybin, studies on psilocin salts have only been disclosed in patent literature until now. Our research aims to fill this gap by assessing the stability of various psilocin salts under multiple degradative conditions. Our experimental results reveal substantial differences in stability linked to the type of counterion used in the salts. These findings suggest that certain salt forms do offer enhanced stability and potential for further development.

**Figure 2:**
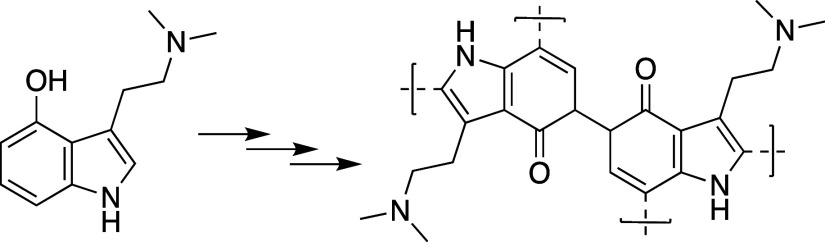
Polymerization of psilocin.

Notably, the ascorbic salt **9d** emerged as the most resistant to degradation across nearly all tested conditions. Its stability in oxidative environments, where a radical reaction triggers polymerization of the molecule (Figure 2),^33^ highlights the protective role of ascorbic acid. The antioxidative properties of ascorbic acid effectively shield psilocin from oxidative degradation, underscoring its potential as a stable formulation. Despite its performance under oxidative conditions, the HCl salt **9a** is less suited for use as a stable therapeutic agent due to its hygroscopic nature and instability in solid form. This finding underscores the potential of the benzoate **9c** and ascorbate **9d** salts as more promising candidates for further investigation.

Literature findings reveal that psilocin freebase is highly unstable in oxidative conditions, showing complete degradation after only two hours.^46^ The significant variances in stability observed among the psilocin salts, suggest that optimizing salt selection is a promising approach to enhance stability and may provide psilocin forms suitable for long-term storage.

One limitation of our study is that the stability testing of the psilocin salts was conducted exclusively in solution. This was an intentional part of study design accelerating degradation to highlight differences between salts, since the stability of compounds in solution is generally lower than the corresponding solids.^47^ To address this limitation, future research should include comprehensive stability testing of psilocin salts in their solid forms, ideally as finished drug products.

Our findings offer valuable insights into the stability of psilocin salts and their potential as substitutes for psilocybin. Utilizing psilocin salts could circumvent the need for the complex phosphorylation process involved in psilocybin production. Additionally, psilocin salts may offer pharmacokinetic advantages, such as a faster onset and more controlled dosing, potentially reducing side effects.

## Conclusions

Our study has explored two alternative strategies aimed at simplifying psilocybin production: the development of psilocin ester prodrugs and the use of psilocin salts. These approaches offer simpler synthesis pathways and possibly improved pharmacokinetic profiles, presenting more practical and cost-effective alternatives to psilocybin with comparable therapeutic effects. Following optimization, the synthesis cost of psilocin esters is anticipated to be comparable to, or potentially lower than, that of psilocybin, owing to the simplified process and use of cost-effective reagents. For example, the cost of ethyl chloroformate is 7.5 EUR/mol compared to 21 EUR/mol and 772 EUR/mol for POCl_3_ and TBPP, respectively.^48^ In the case of psilocin salts, the cost is expected to be even lower, as their preparation eliminates one synthesis step, further reducing material and operational expenses. Moreover, salt formation requires only simple addition of the appropriate acid and evaporation to afford quantitative yields, in contrast to the complex and moderately yielding direct phosphorylation^11^ or TBPP^4^ methods for psilocybin production. Each strategy, however, presents specific challenges. For psilocin ester prodrugs, ensuring rapid hydrolysis to psilocin is critical to prevent any pharmacological activity from the prodrug itself. Additionally, adequate solubility is essential for effective systemic absorption. For psilocin salts, the primary challenge lies in enhancing chemical stability to prevent degradation during storage and handling.

An important aspect to consider is the likelihood of these compounds gaining approval as pharmaceuticals. Compounds containing O-sulfate and O-borate groups are already present in approved drugs, which may facilitate the regulatory approval process.^49−51^ Moreover, ascorbic acid is considered safe and is utilized in various pharmaceutical formulations, including IV, supporting the potential acceptability of these alternatives from a safety standpoint.^52^

For psilocin esters, we evaluated metabolic and chemical stability, as well as solubility. Psilocin salts were evaluated for chemical stability to determine their resilience against degradation during storage and handling. Our study has identified several psilocin ester prodrugs and psilocin salts that exhibit desirable properties, indicating their potential as viable alternatives to psilocybin.

Given the increasing demand for medical-grade psilocybin and its potential upcoming approval as pharmaceutical, it is important to continually explore innovative production strategies. This study represents a preliminary step in exploring alternative methods for psilocybin production. As the acceptance and use of medical-grade psilocybin continue to evolve, the production strategies examined here have the potential to improve the scalability and affordability of treatments. Such improvements could, in turn, contribute to broader access to these therapies, potentially addressing mental health needs more efficiently and equitably. Further research and optimization of these alternative compounds may support future advancements in psychiatric and neurological treatment approaches.

## Experimental Section

### Chemistry

The solvent used where HPLC grade. All commercial reagents were used as received. Analytical HPLC/ESI-MS was performed using UV detection (214, 254, and 280 nm) and electrospray ionization (ESI) MS on a Kinetex C18 column (50 × 3.0 mm, 2.6 μm particle size, 100 Å pore size) with gradients of acetonitrile in 0.05% aqueous HCOOH as mobile phase at a flow rate of 1.5 mL/min. All compounds are >95% pure by HPLC analysis. Preparative HPLC was performed on an Agilent system, using a VP 100*32 mm NUCLEODUR C18 HTEC 5 μM column from Macheraey-Nagel. Thin layer chromatography (TLC) was performed on silica gel plates (Merck) and the plates were visualized under UV light (254 nm). Reactions were not performed under inert atmosphere, unless stated otherwise. ^1^H and ^13^C NMR spectra were recorded using a Bruker DPX400 spectrometer (400 MHz) or Bruker Avance Neo (600 MHz) using deuterated DMSO unless otherwise stated. Chemical shifts are reported in parts per million (ppm) and referenced, unless otherwise stated, to residual solvent (δH = 2.54, δC = 40.45). Coupling constants are reported in Hz. Organic phases were dried over anhydrous sodium sulfate. Hydrogenation was performed with stainless steel high-pressure reactor HEL CAT-7 (7 × 10 mL).

### 4-Benzyloxyindol-3-yl-*N,N*-dimethylglyoxylamide (**2**)^3^

4-benzyloxyindole **1** (10.0 g, 44.8 mmol, 1.0 equiv) was dissolved in dry Et_2_O (245 mL) in a 3-necked flask equipped with a thermometer and cooled on an ice-salt bath to 0 °C. The flask was evacuated and filled with nitrogen. Oxalyl chloride (4.99 mL, 58.2 mmol, 1.3 equiv) diluted in dry Et_2_O (15 mL) was added dropwise to maintain an internal temperature of 0–5 °C. The solution was stirred for 15 min in an ice bath, then at room temperature for 3h with a gentle nitrogen sparge to remove evolved HCl. The nitrogen sparge was removed and the solution was cooled on an ice-salt bath to an internal temperature of 0 °C. A solution of 2.0 M dimethylamine in THF (53 mL, 105.9 mmol, 2.4 equiv) was added dropwise at a rate sufficient to maintain an internal temperature of 0–5 °C. A couple of minutes after the addition, an off-white precipitate started to form. Analysis with LCMS showed that the reaction was halfway done, and more dimethylamine was added (22.0 mL, 47.9 mmol, 1.1 equiv). The reaction was stirred for 3h in room temperature yielding an off-white slurry. The precipitate was collected with a P4 glass funnel by suction filtration and subsequently suspended in distilled H2O (150 mL) to remove dimethylamine hydrochloride. The suspension was stirred for 1h, then filtered and washed with H2O (3 × 50 mL) and hexane (50 mL). The collected solids were dried overnight on the vacuum line to afford a caramel-colored powder of **2**. The product was 98% pure according to LCMS and NMR showed all the correct peaks according to the reference. Yield: 78% (11.2 g, 34.8 mmol). ^**1**^**H NMR** (600 MHz, DMSO-*d*_6_) δ 12.27 (s, 1H), 8.06 (d, *J* = 3.2 Hz, 1H), 7.61 (d, *J* = 7.4 Hz, 2H), 7.37 (dd, *J* = 7.6 Hz, 2H), 7.28 (t, *J* = 7.3 Hz, 1H), 7.13–7.07 (m, 2H), 6.70 (dd, *J* = 7.2, 1.5 Hz, 1H), 5.26 (s, 2H), 2.92 (s, 3H), 2.89 (s, 3H). ^**13**^**C NMR** (151 MHz, DMSO) δ 186.3, 168.4, 152.3, 138.9, 137.6, 135.6, 128.2, 127.2, 126.8, 124.1, 114.4, 114.1, 105.6, 104.4, 69.3, 36.6, 33.2.

### 4-Benzyloxy-*N*,*N*-dimethyltryptamine (**3**)^4^

The indole **2** (11.2 g, 34.8 mmol, 1.0 equiv) was suspended in anhydrous 2-MeTHF (250 mL) and cooled to −78 °C. The ice bath was removed and while stirring, LiAlH_4_ pellets (3.69 g, 97.5 mmol, 2.8 equiv) were added to the solution. The solution was allowed to warm to RT and a reflux condenser was fitted to the flask. With continuous stirring, the mixture was heated to reflux for 8h. The solution was allowed to cool to RT and sodium sulfate decahydrate (38.7 g, 120 mmol, 1.23 equiv) was added carefully. The solution was stirred for 15 min followed by the addition of 20g of Celite. Thereafter, isopropanol (60 mL) was added, and the mixture was stirred for another 30 min. THF (50 mL) was added to assist in the breakup of the thick slurry. A Buchner funnel packed with a thin pad of Celite, fitted with a filter paper on top was used to filter the slurry. The vacuum was turned off, THF (50 mL) was added, and the filter cake was broken up and mixed using a spoon. The slurry was filtered again, and the process was repeated three times. All of the organic filtrates were combined and concentrated under vacuum to provide a light brown oil. The oil was dissolved in EtOAc (50 mL) and concentrated again to provide a beige solid. Recrystallization from EtOAc provided beige crystals of **3**. The product was >99% pure according to LCMS and NMR showed all the correct peaks according to the reference. Yield: 84% (8.56 g, 29.1 mmol). ^**1**^**H NMR** (400 MHz, CDCl_3_) δ 8.18 (s, 1H), 7.53–7.48 (m, 2H), 7.42–7.36 (m, 2H), 7.33 (dd, *J* = 8.4, 6.1 Hz, 1H), 7.06 (dd, *J* = 7.9 Hz, 1H), 6.96 (d, *J* = 8.2 Hz, 1H), 6.89 (s, 1H), 6.55 (d, *J* = 7.7 Hz, 1H), 5.18 (s, 2H), 3.11–3.05 (m, 2H), 2.69–2.61 (m, 2H), 2.16 (s, 6H). ^**13**^**C NMR** (101 MHz, CDCl_3_) δ 154.2, 140.4, 138.6, 137.8, 131.1, 128.9, 128.3, 123.1, 121.1, 117.8, 105.2, 100.8, 70.4, 61.7, 45.3, 25.2. HRMS-ESI calcd for: C_19_H_23_N_2_O [M + H]+: 295.1810. Found: 295.1807

### General Procedure for Psilocin Esters (**4a–4k**)^3^

and novel procedures A 10 mL glass vial was charged with 3 (150 mg, 0.51 mmol, 1.0 equiv), 10% palladium on charcoal (75.0 mg, 0.07 mmol, 0.14 equiv) and dissolved in anhydrous toluene/THF (7.5 mL). After the vial was placed in the hydrogenation apparatus, it was filled with hydrogen, purged three times, filled to 60 psi, and left stirring for 6 h at RT. The solution was thereafter filtered in darkness, under nitrogen, through a thin pad of Celite into a vial charged with cesium carbonate (199 mg, 0.61 mmol, 1.2 equiv) and anhydride/acyl chloride. After stirring for 24 h, the mixture was filtered through a syringe filter (pore size 0.45 μm) and the solvent was evaporated. The crude oil was dissolved in a small amount of (MeOH/50 mM NH_4_HCO_3_ buffer) (1:1) and purified using preparative HPLC (MeCN/50 mM NH_4_HCO_3_ buffer). Collected fractions were concentrated on the freeze-dryer for 24 h, then the remaining powder was dissolved in 1 mL of water and subjected to another freeze-drying cycle to remove residual NH_4_HCO_3_. After evaporation, the oils/semisolids were dissolved in a small amount of EtOH, and 1.0 equiv fumaric acid dissolved in EtOH (30.0 mg/mL) was added. The precipitate was filtered, washing with 2 mL EtOAc, and dried on the vacuum line overnight providing **4a**–**k** as off-white solids. The products were analyzed with LCMS and NMR.

### Compound **4a**

The compound was synthesized using the general method using acetic anhydride (67.4 μL, 0.71 mmol, 1.4 equiv) and toluene as solvent. Purification was performed using preparative HPLC (gradient 0–40% MeCN, 50 mM NH_4_HCO_3_ buffer). HPLC purity: 98.0%. Yield: 14% (17.7 mg, 0.07 mmol). ^**1**^**H NMR** (500 MHz, DMSO-*d*_6_) δ 11.18 (s, 1H), 7.24 (dd, *J* = 8.1, 0.7 Hz, 1H), 7.19 (d, *J* = 2.3 Hz, 1H), 7.05 (dd, *J* = 7.9 Hz, 1H), 6.69 (dd, *J* = 7.6, 0.6 Hz, 1H), 6.50 (s, 3H), 2.92 (ddd, *J* = 9.6, 5.5, 2.1 Hz, 2H), 2.86 (ddd, *J* = 12.0, 5.9, 2.2 Hz, 2H), 2.50 (s, 6H), 2.37 (s, 3H). ^**13**^**C NMR** (126 MHz, DMSO-*d*_6_) δ 169.7, 167.8, 143.5, 138.5, 135.0, 123.9, 121.1, 119.3, 111.6, 109.5, 109.5, 58.9, 43.4, 22.5, 20.8. HRMS-ESI calcd for: C_14_H_19_N_2_O_2_ [M + H]+: 247.1447. Found: 247.1450.

### Compound **4b**

The compound was synthesized with the general method using butyric anhydride (117 μL, 0.71 mmol, 1.4 equiv) and toluene as solvent. Purification was performed using preparative HPLC (gradient 20–60% MeCN, 50 mM NH_4_HCO_3_ buffer). HPLC purity 96.5%. Yield: 18% (24.6 mg, 0.09 mmol). ^**1**^**H NMR** (500 MHz, DMSO-*d*_6_) δ 11.16 (s, 1H), 7.26–7.22 (m, 1H), 7.19 (d, *J* = 2.3 Hz, 1H), 7.05 (dd, *J* = 7.9 Hz, 1H), 6.69–6.65 (m, 1H), 6.53 (s, 2H), 2.92 (s, 4H), 2.69 (t, *J* = 7.3 Hz, 2H), 2.52 (s, 6H), 1.69 (h, *J* = 7.4 Hz, 2H), 0.99 (t, *J* = 7.4 Hz, 3H). ^**13**^**C NMR** (126 MHz, DMSO-*d*_6_) δ 172.1, 167.5, 143.5, 138.5, 134.8, 123.7, 121.2, 119.4, 111.6, 109.4, 109.3, 58.5, 43.3, 35.2, 22.4, 17.8, 13.5. HRMS-ESI calcd for: C_16_H_23_N_2_O_2_ [M + H]+: 275.1760. Found: 275.1761.

### Compound **4c**

The compound was synthesized with the general method using pentanoic anhydride (212 μL, 0.71 mmol, 1.4 equiv) and toluene as solvent. Purification was performed using preparative HPLC (gradient 20–60% MeCN, 50 mM NH_4_HCO_3_ buffer). HPLC purity 96.4%. Yield: 11% (15.7 mg, 0.05 mmol). ^**1**^**H NMR** (500 MHz, DMSO-*d*_6_) δ 11.17 (s, 1H), 7.26–7.22 (m, 1H), 7.19 (d, *J* = 2.3 Hz, 1H), 7.05 (dd, *J* = 7.9 Hz, 1H), 6.69–6.65 (m, 1H), 6.54 (s, 2H), 2.94 (s, 4H), 2.70 (t, *J* = 7.5 Hz, 2H), 2.54 (s, 6H), 1.70–1.61 (m, 2H), 1.44–1.35 (m, 2H), 0.92 (t, *J* = 7.4 Hz, 3H). ^**13**^**C NMR** (126 MHz, DMSO-*d_6_*) δ 172.3, 167.4, 143.5, 138.5, 134.8, 123.7, 121.2, 119.3, 111.6, 109.4, 109.2, 58.4, 43.2, 33.1, 26.4, 22.3, 21.7, 13.7. HRMS-ESI calcd for: C_17_H_25_N_2_O_2_ [M + H]+: 289.1916. Found: 289.1918.

### Compound **4d**

The compound was synthesized with the general method using isobutyl chloroformate (167 μL, 1.02 mmol, 2.0 equiv) and THF as solvent. Purification was performed using preparative HPLC (gradient 20–60% 50 mM, NH_4_HCO_3_ buffer). HPLC purity 95.1%. As the product was a fine white solid, no fumarate treatment was needed. Yield: 23% (35.8 mg, 0.12 mmol). ^**1**^**H NMR** (500 MHz, DMSO-*d*_6_) δ 11.16 (s, 1H), 8.27 (s, 1H), 7.27 (dd, *J* = 8.1, 0.6 Hz, 1H), 7.20 (d, *J* = 2.2 Hz, 1H), 7.05 (dd, *J* = 7.9 Hz, 1H), 6.78 (dd, *J* = 7.6, 0.6 Hz, 1H), 4.02 (d, *J* = 6.6 Hz, 2H), 2.88–2.79 (m, 2H), 2.71–2.62 (m, 2H), 2.34 (s, 6H), 1.98 (m, *J* = 6.7 Hz, 1H), 0.95 (d, *J* = 6.7 Hz, 6H). ^**13**^**C NMR** (126 MHz, DMSO-*d*_6_) δ 164.4, 153.6, 143.9, 138.5, 124.0, 121.0, 119.2, 111.0, 110.1, 109.8, 74.2, 59.7, 44.2, 27.3, 23.1, 18.6. HRMS-ESI calcd for: C_17_H_25_N_2_O_3_ [M + H]+: 305.1865. Found: 305.1863.

### Compound **4e**

The compound was synthesized with the general method using dimethyl dicarbonate (76.5 μL, 0.71 mmol, 1.4 equiv) and toluene as solvent. Purification was performed using preparative HPLC (gradient 0–40% MeCN, 50 mM NH_4_HCO_3_ buffer). HPLC purity 95.6%. Yield: 2% (2.18 mg, 0.01 mmol). ^**1**^**H NMR** (500 MHz, DMSO-*d*_6_) δ 11.24 (s, 1H), 7.30–7.27 (m, 1H), 7.24 (d, *J* = 2.3 Hz, 1H), 7.07 (dd, *J* = 7.9 Hz, 1H), 6.84–6.80 (m, 1H), 6.57 (s, 5H), 3.87 (s, 3H), 3.01–2.98 (m, 2H), 2.96–2.92 (m, 2H), 2.60 (s, 6H). ^**13**^**C NMR** (126 MHz, DMSO-*d*_6_) δ 166.7, 154.1, 143.8, 138.6, 134.4, 124.4, 121.3, 119.0, 111.1, 110.0, 108.6, 58.3, 55.5, 42.9, 21.8. HRMS-ESI calcd for: C_14_H_19_N_2_O_3_ [M + H]+: 263.1396. Found: 263.1400.

### Compound **4f**

The compound was synthesized with the general method using benzyl chloroformate (144 μL, 1.02 mmol, 2.0 equiv) and THF as solvent. Purification was performed using preparative HPLC (gradient 20–90% MeCN 50 mM, NH_4_HCO_3_ buffer). HPLC purity (acidic) 92.3% Yield: 1% (1.59 mg, 0.01 mmol). ^**1**^**H NMR** (500 MHz, DMSO-*d*_6_) δ 11.20 (s, 1H), 8.32 (s, 1H), 7.49–7.38 (m, 6H), 7.29–7.26 (m, 1H), 7.19 (d, *J* = 2.2 Hz, 1H), 7.06 (dd, *J* = 7.9 Hz, 1H), 6.81–6.78 (m, 1H), 6.47 (s, 19H), 5.29 (s, 2H), 2.83–2.78 (m, 2H), 2.68–2.62 (m, 2H), 2.29 (s, 6H). ^**13**^**C NMR** (126 MHz, DMSO-*d*_6_) δ 167.6, 164.4, 153.5, 143.9, 138.6, 135.1, 128.6, 128.6, 128.5, 124.2, 121.1, 119.2, 111.1, 109.9, 69.9, 59.5, 43.9, 22.8. HRMS-ESI calcd for: C_20_H_23_N_2_O_3_ [M + H]+: 339.1709. Found: 339.1717.

### Compound **4g**

The compound was synthesized with the general method using cyclopropane carbonyl chloride (92.5 μL, 1.02 mmol, 2.0 equiv) and THF as solvent. Purification was performed using preparative HPLC (gradient 0–50% MeCN, 50 mM NH_4_HCO_3_ buffer). HPLC purity 98.4% Yield: 10% (13.5 mg, 0.05 mmol). ^**1**^**H NMR** (500 MHz, DMSO-*d*_6_) δ 11.17 (s, 1H), 7.25–7.22 (m, 1H), 7.18 (d, *J* = 2.2 Hz, 1H), 7.03 (dd, *J* = 7.9 Hz, 1H), 6.68–6.64 (m, 1H), 6.48 (s, 3H), 2.90 (dd, *J* = 9.4, 5.5 Hz, 2H), 2.83 (dd, *J* = 9.4, 5.3 Hz, 2H), 2.46 (s, 6H), 2.02 (tt, *J* = 7.7, 4.8 Hz, 1H), 1.10–0.99 (m, 4H). ^**13**^**C NMR** (126 MHz, DMSO-*d*_6_) δ 173.5, 167.8, 143.6, 138.5, 135.1, 123.8, 121.1, 119.5, 111.5, 109.9, 109.5, 58.9, 43.6, 22.6, 12.6, 9.0. HRMS-ESI calcd for: C_16_H_21_N_2_O_2_ [M + H]+: 273.1603. Found: 273.1613.

### Compound **4h**

The compound was synthesized with the general method using benzoic anhydride (161 mg, 0.71 mmol, 1.4 equiv) and toluene as solvent. Purification was performed using preparative HPLC (gradient 40–100% MeCN, 50 mM NH_4_HCO_3_ buffer). HPLC purity >99%. Yield: 6% (10.1 mg, 0.03 mmol). ^**1**^**H NMR** (500 MHz, DMSO-*d*_6_) δ 11.20 (s, 1H), 8.25–8.20 (m, 2H), 7.77 (dd, *J* = 7.5 Hz, 1H), 7.64 (dd, *J* = 7.8 Hz, 2H), 7.31 (d, *J* = 8.1 Hz, 1H), 7.20 (d, *J* = 2.0 Hz, 1H), 7.11 (dd, *J* = 7.9 Hz, 1H), 6.82 (d, *J* = 7.5 Hz, 1H), 6.50 (s, 1H), 2.82–2.76 (m, 2H), 2.70–2.64 (m, 2H), 2.11 (s, 6H). ^**13**^**C NMR** (126 MHz, DMSO-*d*_6_) δ 167.6, 165.1, 143.6, 138.6, 134.9, 134.1, 129.9, 129.1, 123.9, 121.2, 119.6, 111.7, 110.1, 109.7, 59.2, 43.6, 23.0. HRMS-ESI calcd for: C_19_H_21_N_2_O_2_ [M + H]+: 309.1603. Found: 309.1605.

### Compound **4i**

The compound was synthesized with the general method using ethyl chloroformate (97.0 μL, 1.02 mmol, 2.0 equiv) and THF as solvent. Purification was performed using preparative HPLC (gradient 0–50% MeCN, 50 mM NH_4_HCO_3_ buffer). HPLC purity >99%. Yield: 6% (8.56 mg, 0.03 mmol). ^**1**^**H NMR** (500 MHz, DMSO-*d*_6_) δ 11.23 (s, 1H), 7.28 (d, *J* = 8.1 Hz, 1H), 7.23 (d, *J* = 2.1 Hz, 1H), 7.07 (dd, *J* = 7.9 Hz, 1H), 6.81 (d, *J* = 7.6 Hz, 1H), 6.54 (s, 2H), 4.28 (q, *J* = 7.1 Hz, 2H), 2.94 (s, 3H), 2.55 (s, 5H), 2.51–2.49 (m, 3H), 1.30 (t, *J* = 7.1 Hz, 3H). ^**13**^**C NMR** (126 MHz, DMSO-*d*_6_) δ 167.3, 153.5, 143.8, 138.6, 134.7, 124.3, 121.3, 119.1, 111.2, 109.9, 108.9, 64.7, 58.5, 43.0, 21.9, 14.1. HRMS-ESI calcd for: C_15_H_21_N_2_O_3_ [M + H]+: 277.1552. Found: 277.1558.

### Compound **4j**

The compound was synthesized with the general method using undecanoyl chloride (87.5 μL, 0.82 mmol, 2.0 equiv) and THF as solvent. Purification was performed using preparative HPLC (gradient 40–100% MeCN, 50 mM NH_4_HCO_3_ buffer). HPLC purity (acidic) > 99%. Yield: 1% (2.07 mg, 0.01 mmol). ^**1**^**H NMR** (500 MHz, DMSO-*d*_6_) δ 11.15 (s, 1H), 7.23 (d, *J* = 8.1 Hz, 1H), 7.18–7.15 (m, 1H), 7.03 (dd, *J* = 7.9 Hz, 1H), 6.65 (d, *J* = 7.6 Hz, 1H), 6.48 (s, 19H), 2.89–2.83 (m, 2H), 2.76 (q, *J* = 7.1 Hz, 2H), 2.68 (t, *J* = 7.4 Hz, 2H), 2.41 (s, 6H), 1.66 (p, *J* = 7.4 Hz, 2H), 1.40–1.33 (m, 2H), 1.24 (s, 18H). ^**13**^**C NMR** (126 MHz, DMSO-*d_6_*) δ 174.6, 172.3, 167.5, 143.5, 138.5, 135.0, 123.7, 121.0, 119.4, 111.5, 109.9, 109.4, 59.2, 43.8, 33.8, 33.4, 31.3, 28.9, 28.7, 28.7, 28.6, 24.5, 24.3, 22.1, 14.0. HRMS-ESI calcd for: C_23_H_37_N_2_O_2_ [M + H]+: 373.2855. Found: 373.2856.

### Compound **4k**

The compound was synthesized with the general method using 4-morpholinecarbonyl chloride (93.9 μL, 0.82 mmol, 1.6 equiv). Purification was performed using preparative HPLC (gradient 20–60% MeCN, 50 mM NH_4_HCO_3_ buffer). HPLC purity >99%. Yield: 31% (50.5 mg, 0.16 mmol). ^**1**^**H NMR** (500 MHz, DMSO-*d*_6_) δ 11.05 (s, 1H), 7.20 (d, *J* = 8.0 Hz, 1H), 7.14 (d, *J* = 2.0 Hz, 1H), 7.01 (dd, *J* = 7.9 Hz, 1H), 6.62 (d, *J* = 7.5 Hz, 1H), 6.51 (s, 1H), 3.64 (s, 2H), 3.42 (s, 2H), 2.92–2.83 (m, 2H), 2.78–2.69 (m, 2H), 2.38 (s, 6H), 1.61 (s, 4H), 1.54(s, 2H). ^**13**^**C NMR** (126 MHz, DMSO-*d*_6_) δ 167.6, 153.4, 144.5, 138.4, 134.9, 123.1, 121.0, 120.2, 111.8, 110.3, 108.9, 59.1, 45.0, 44.5, 44.0, 25.5, 25.2, 23.7, 23.1. HRMS-ESI calcd for: C_18_H_26_N_3_O_2_ [M + H]+: 316.2025. Found: 316.2036.

### Compound **5**(31)

A 10 mL glass vial was charged with **3** (150 mg, 0.51 mmol, 1.0 equiv), and 10% palladium on charcoal (75.0 mg, 0.07 mmol, 0.14 equiv). Anhydrous toluene (7.5 mL) was added, and the vial was placed in the hydrogenation apparatus. The apparatus was filled with hydrogen, purged three times, filled to 60 psi, and left stirring for 6 h at RT. The solution was thereafter filtered in darkness, under nitrogen, through a thin pad of Celite into a vial charged with boric acid (31.5 mg, 0.51 mmol, 1.0 equiv). The solution was heated to 80 °C for three hours while continuously stirring. After cooling to room temperature, the solvents were evaporated leaving a brown oil. Purification was performed using preparative HPLC (gradient 0–10% MeCN, 50 mM NH_4_HCO_3_ buffer). Collected fractions were concentrated on the freeze-dryer for 24 h, then the remaining powder was dissolved in 1 mL of water and subjected to another freeze-drying cycle to remove residual NH_4_HCO_3_. After evaporation, the oil was dissolved in a small amount of EtOH, and 1equiv fumaric acid dissolved in EtOH (30 mg/mL) was added. The precipitate was filtered and dried on the vacuum line overnight providing **5** as a brown solid. The product was analyzed with LCMS and NMR. NMR revealed that the compound was isolated as mixed fumarate/formiate salts. Yield: 16% (20.5 mg, 0.083 mmol). ^**1**^**H NMR** (500 MHz, DMSO-*d*_6_) δ 10.70 (s, 1H), 6.96 (d, *J* = 2.1 Hz, 1H), 6.83–6.78 (m, 1H), 6.76 (dd, *J* = 8.0, 0.8 Hz, 1H), 6.49 (s, 2H), 6.31 (dd, *J* = 7.4, 0.8 Hz, 1H), 3.06–2.99 (m, 2H), 2.92 (t, *J* = 7.3 Hz, 2H), 2.50 (s, 6H). ^**13**^**C NMR** (126 MHz, DMSO-*d*_6_) δ 168.0, 151.6, 138.7, 122.0, 121.3, 116.5, 111.1, 103.2, 102.8, 59.6, 43.3, 22.8, 18.6. FT-ICR-MALDI-HRMS calcd for: C_12_H_18_BN_2_O_3_ [M + H]+: 249.1413. Found: 249.1407. (see supplementary for information)

### Compound **6**(30)

A 10 mL glass vial was charged with **3** (150 mg, 0.51 mmol, 1.0 equiv) and 10% palladium on charcoal (75.0 mg, 0.07 mmol, 0.14 equiv). Anhydrous THF (7.5 mL) was added, and the vial was placed in the hydrogenation apparatus. The apparatus was filled with hydrogen, purged three times, filled to 60 psi, and left stirring for 6 h at RT. The solution was thereafter filtered in darkness, under nitrogen, through a thin pad of Celite into a round-bottom flask. The solvent was evaporated, and sulfur trioxide pyridine complex (203 mg, 1.27 mmol, 2.5 equiv) was added. The flask was cycled through vacuum-nitrogen three times and degassed pyridine (20 mL) was added to the flask using a cannula. The solution was stirred at 45 °C for two hours under nitrogen and then at room temperature overnight. Thereafter, the pyridine was evaporated, and the residue was dissolved in 5 mL of (MeOH/50 mM NH_4_HCO_3_ buffer) (1:1). Purification was performed using preparative HPLC (gradient 0–10% MeCN, 50 mM NH_4_HCO_3_ buffer). Collected fractions were concentrated on the freeze-dryer for 24 h, then the remaining powder was dissolved in 1 mL of water and subjected to another freeze-drying cycle to remove residual NH_4_HCO_3_ affording **6** as a dark red-brown powder. The product was analyzed with LCMS and NMR. HPLC purity 98%. Yield: 22% (32.2 mg, 0.11 mmol). ^**1**^**H NMR** (500 MHz, DMSO-*d*_6_) δ 10.93–10.88 (m, 1H), 7.12 (d, *J***=** 2.3 Hz, 1H), 7.06 (ddd, *J***=** 14.1, 7.9, 0.9 Hz, 2H), 6.96 (dd, *J***=** 7.9 Hz, 1H), 3.30 (dd, *J***=** 9.9, 6.4 Hz, 2H), 3.15 (dd, *J***=** 10.1, 6.2 Hz, 2H), 2.78 (s, 6H). ^**13**^**C NMR** (126 MHz, DMSO-*d*_6_) δ 146.8, 138.4, 123.3, 121.4, 119.6, 109.8, 108.7, 107.1, 58.2, 42.6, 21.4.

### Compound **8** (Psilocybin)^4,53^

Step 1: A 10 mL glass vial was charged with (150 mg, 0.51 mmol, 1.0 equiv) of **3**, and 10% palladium on charcoal (75.0 mg, 0.07 mmol, 0.14 equiv). Anhydrous THF (7.5 mL) was added, and the vial was placed in the hydrogenation apparatus. The apparatus was filled with hydrogen, purged three times, filled to 60 psi, and left stirring for 6 h at RT. The solution was thereafter filtered in darkness, under nitrogen, through a thin pad of Celite into a 20 mL vial equipped with a septum. The solution was cooled to −78 °C and a solution of 2.5 M BuLi in hexanes (0.25 mL, 0.61 mmol, 1.2 equiv) was added slowly. The olive-green mixture was stirred for 10 min and tetrabenzyl pyrophosphate (302 mg, 0.56 mmol, 1.1 equiv) was added in one portion. After 1.5 h, the cooling bath was removed, and the temperature was allowed to slowly rise to −25 °C. The solution was stored in the freezer at −25 °C overnight. Basic alumina (1g) was added in one portion and the solution was diluted with EtOAc (10 mL). The mixture was filtered through a thin pad of Celite and washed with EtOAc (4 × 10 mL). The combined filtrates were concentrated in a round-bottomed flask. The gray oil was redissolved in DCM (5 mL) and heated using a heat gun to boil for 5 min. The solution was allowed to reach room temperature and was then stored in the fridge overnight. The blue-gray precipitate was filtered using a Büchner funnel and washed with DCM (3 × 5 mL). The precipitate was transferred to a round-bottomed flask and dried overnight to provide a light-purple solid of the intermediate [CAS: 562105–76–4] (**7**) (90 mg, 38%). The intermediate was directly used in the next step. Step 2: A 10 mL glass vial was charged with **7** (90 mg, 0.193 mmol) and 10% palladium on charcoal (20.0 mg, 0.02 mmol, 0.10 equiv). MeOH (6 mL) was added, and the vial was placed in the hydrogenation apparatus. The apparatus was filled with hydrogen, purged three times, filled to 60 psi, and left stirring for one hour at RT. The solution was filtered through a thin pad of Celite and washed with MeOH (3 × 10 mL). The combined filtrates were concentrated on the rotary evaporator and dried overnight on the vacuum line providing a transparent solid. Purification was performed using preparative HPLC (gradient 0–10% MeCN, 50 mM NH_4_HCO_3_ buffer). Collected fractions were concentrated on the freeze-dryer for 24 h, then the remaining powder was dissolved in 1 mL of water and subjected to another freeze-drying cycle to remove residual NH_4_HCO_3_ affording **8** as a white fluffy powder. The product was analyzed with LCMS and NMR. Yield: 39% (21.6 mg, 0.08 mmol). ^**1**^**H NMR** (500 MHz, DMSO-*d*_6_) δ 10.89–10.84 (m, 1H), 6.98 (dd, *J* = 7.9, 2.2 Hz, 2H), 6.91 (q, *J* = 4.5 Hz, 2H), 3.16 (s, 4H), 2.65 (s, 6H). ^**13**^**C NMR** (126 MHz, DMSO-*d*_6_) δ 147.4, 138.4, 121.6, 119.3, 109.8, 108.8, 106.0, 58.3, 42.1, 21.8.

### General Procedure for Preparation of Psilocin Salts

A 10 mL glass vial was charged with **3** (150 mg, 0.51 mmol, 1.0 equiv), 10% palladium on charcoal (75.0 mg, 0.07 mmol, 0.14 equiv) and dissolved in anhydrous THF (7.5 mL). After the vial was placed in the hydrogenation apparatus, it was filled with hydrogen, purged three times, filled to 60 psi, and left stirring for 6 h at RT. The solution was thereafter filtered in darkness, under nitrogen, through a thin pad of Celite into a vial. The solvent was evaporated to weigh the exact amount of produced psilocin. Thereafter, psilocin **12** (1 equiv) was dissolved in 5 mL THF under inert atmosphere. The acid (1 equiv) (except HCl where 2.3 equiv was added^1^) was dissolved in 2 mL isopropanol and, if required for dissolution, heated carefully, and added to the solution. The mixture was stirred for 30 min before solvent evaporation.

^1^due to degradation of psilocin when 1 equiv HCl was added, we decided to use an excess of HCl as the residual acid can be evaporated easily.

### Compound **9a** HCl Salt

The compound was synthesized using the general method using HCl 4 M (in dioxane) (0.149 mL, 0.597 mmol, 2.3 equiv). Yield: 62.4 mg (quantitative). Appearance: White crystals. The product was >99% pure according to LCMS. ^**1**^**H NMR** (500 MHz, DMSO-*d*_6_) δ 10.83 (s, 1H), 9.58 (s, 1H), 7.03 (d, *J* = 2.3 Hz, 1H), 6.83 (t, 1H), 6.78 (dd, *J* = 8.1, 0.9 Hz, 1H), 6.37 (dd, *J* = 7.3, 0.9 Hz, 1H), 3.33–3.28 (m, 2H), 3.20–3.16 (m, 2H), 2.78 (d, *J* = 4.6 Hz, 6H). ^**13**^**C NMR** (126 MHz, DMSO-*d*_6_) δ 151.4, 138.7, 122.2, 121.7, 116.2, 109.0, 103.1, 103.0, 58.2, 42.1, 21.5.

### Compound **9b** Borate Salt

The compound was synthesized using the general method using boric acid (16 mg, 0.259 mmol, 1 equiv). Yield: 69.0 mg (quantitative). Appearance: White powder. The product was 98% pure according to LCMS. ^**1**^**H NMR** (500 MHz, DMSO-*d*_6_) δ 10.58 (s, 1H), 6.91 (d, *J* = 2.2 Hz, 1H), 6.79 (t, 1H), 6.73 (dd, *J* = 8.0, 0.9 Hz, 1H), 6.25 (dd, *J* = 7.4, 0.9 Hz, 1H), 2.87 (t, *J* = 6.7 Hz, 2H), 2.55 (t, *J* = 6.7 Hz, 2H), 2.22 (s, 6H). ^**13**^**C NMR** (126 MHz, DMSO-*d*_6_) δ 151.8, 138.7, 121.8, 121.1, 116.9, 112.9, 103.52, 102.7, 61.4, 45.1, 24.5.

### Compound **9c** Benzoic Salt

The compound was synthesized using the general method using benzoic acid (32 mg, 0.259 mmol, 1 equiv). Yield: 84.7 mg (quantitative). Appearance: White powder. The product was 95% pure according to LCMS. ^**1**^**H NMR** (600 MHz, DMSO-*d*_6_) δ 10.60 (s, 1H), 7.94 (d, *J* = 7.8 Hz, 2H), 7.56 (t, *J* = 7.2 Hz, 1H), 7.46 (t, *J* = 7.6 Hz, 2H), 6.93 (d, 1H), 6.80 (t, *J* = 7.7 Hz, 1H), 6.75 (d, *J* = 8.0 Hz, 1H), 6.27 (d, *J* = 7.4 Hz, 1H), 2.94 (t, *J* = 7.0 Hz, 2H), 2.69 (t, *J* = 7.0 Hz, 2H), 2.33 (s, 6H). ^**13**^**C NMR** (150 MHz, DMSO-*d*_6_) δ 167.9, 151.9, 138.9, 132.2, 129.3, 128.4, 122.0, 121.3, 116.9, 112.4, 103.5, 102.9, 60.9, 44.6, 24.1.

### Compound **9d** Ascorbate Salt

The compound was synthesized using the general method using ascorbic acid (46 mg, 0.259 mmol, 1 equiv). Yield: 99.0 mg (quantitative). Appearance: Yellow powder. The product was 95% pure according to LCMS. ^**1**^**H NMR** (500 MHz, DMSO-*d*_6_) δ 10.65 (s, 1H), 6.95 (d, *J* = 2.2 Hz, 1H), 6.83–6.78 (m, 1H), 6.75 (dd, *J* = 8.1, 0.9 Hz, 1H), 6.28 (dd, *J* = 7.4, 0.9 Hz, 1H), 4.51 (s, 1H), 3.77 (dt, *J* = 12.2, 6.1 Hz, 1H), 3.62 (td, *J* = 6.7, 2.6 Hz, 1H), 3.43–3.41 (m, 2H), 2.96 (t, *J* = 7.1 Hz, 2H), 2.80 (t, *J* = 7.0 Hz, 2H), 2.41 (s, 6H). ^**13**^**C NMR** (126 MHz, DMSO-*d*_6_) δ 171.4, 151.7, 138.7, 122.0, 121.4, 116.7, 111.6, 103.4, 102.9, 75.9, 69.4, 62.1, 60.5, 44.2, 25.5.

### Compound **9e** Phosphate Salt

The compound was synthesized using the general method using phosphoric acid (19 μL, 0.259 mmol, 1 equiv). Yield: 84.8 mg (quantitative). Appearance: White powder. The product was 97% pure according to LCMS. ^**1**^**H NMR** (600 MHz, DMSO-*d*_6_) δ 10.69 (s, 1H), 6.98 (d, 1H), 6.81 (t, *J* = 7.8 Hz, 1H), 6.76 (d, *J* = 8.0 Hz, 1H), 6.31 (d, *J* = 7.4 Hz, 1H), 3.08–3.03 (m, 2H), 3.02–2.96 (m, 2H), 2.56 (s, 6H). ^**13**^**C NMR** (150 MHz, DMSO-*d*_6_) δ 151.5, 138.7, 122.0, 121.5, 116.5, 110.6, 103.2, 102.9, 59.5, 43.3, 22.7.

### Compound **9f** TFA Salt

The compound was synthesized using the general method using TFA (19 μL, 0.259 mmol, 1 equiv). Yield: 82.6 mg (quantitative). Appearance: Light brown crystals. The product was 99% pure according to LCMS. ^**1**^**H NMR** (600 MHz, DMSO-*d*_6_) δ 10.77 (s, 1H), 7.03 (d, *J* = 2.0 Hz, 1H), 6.84 (t, 1H), 6.79 (d, *J* = 8.0 Hz, 1H), 6.34 (d, *J* = 7.4 Hz, 1H), 3.26–3.23 (m, 2H), 3.14–3.10 (m, 2H), 2.76 (s, 6H). ^**13**^**C NMR** (150 MHz, DMSO-*d*_6_) δ 151.4, 150.3, 138.7, 122.2, 121.7, 116.3, 109.2, 103.4, 103.1, 58.8, 42.7, 22.0.

## Stability Testing of Psilocin Esters

### Evaluation of Chemical Stability

Ten μL of HCl (2M)/ NaHCO_3_ (1M) containing internal reference (5-(2-nitro-propenyl)-benzo(1,3)dioxole) (10 mM) was added to 10 μL (10 mM DMSO stock) of each test compound. The solutions were mixed and stored at room temperature. The concentration of psilocin and parent compound were analyzed at 0–24 h using LC-MS. The concentration of both parent prodrug and psilocin liberated were expressed relative to the internal standard.

### Evaluation of Stability in Simulated Stomach Acid^54^

Ten μL of HCl (0.02 M) containing internal reference (5-(2-nitro-propenyl)-benzo(1,3)dioxole) (10 mM) was added to 10 μL (10 mM DMSO stock) of each test compound. The solutions were mixed and stored at room temperature. The concentration of psilocin and parent compound were analyzed at various time points using LC-MS. The concentration of both parent prodrug and psilocin liberated were expressed relative to the internal standard.

### Microsome Assay Method Description

The assay was run at 37 °C with a microsomal protein concentration of 0.5 mg/mL and a compound concentration of 1 μM in a 0.1 M potassium phosphate buffer at pH 7.4. The compounds were added to the microsome suspension whereafter the enzymatic reaction was initiated by the addition of the cofactor NADPH, giving a final NADPH concentration of 1 mM. Samples at six time points (0, 5, 10, 15, 25, and 45 min) were withdrawn from the incubation and the enzymatic reaction was terminated by protein precipitation with an equal volume of acetonitrile. After a centrifugation step, an aliquot of the resulting supernatant was mixed with three aliquots of milli Q water before analysis with tandem mass spectrometry (LC-MS/MS).

## Forced Degradation Studies of Psilocin Salts

### Preparation of Stock and Standard Solutions

Stock solutions of psilocin salts were prepared at a concentration equivalent to 4.0 mg/mL of freebase psilocin in acetonitrile:deionized water mixture 1/1 (v/v). Each stock solution was then mixed with an equal volume of a degradant solution (1.0 M HCl, 0.10 M NaOH, 0.30% H_2_O_2_) or with deionized water in the case of thermal and photolytic stability experiments. This method of dilution adjusted the concentration to 2.0 mg/mL of freebase psilocin, which ensured uniform experimental conditions across all tests.

The internal standard (IS) stock was prepared by dissolving 3.5 mg indole acetic acid in 4 mL acetonitrile:deionized water mixture 1/1 (v/v) to reach a concentration of 5 mM.

### Acid, Basic and Oxidative Degradation

The psilocin salt stock solutions were treated with 1.0 M HCl, 0.10 M NaOH or 0.30% H_2_O_2_ at room temperature. The decrease in psilocin concentration was analyzed from 0 to 24 h using LC-MS.

### Thermal Degradation

For thermal stress testing, an aliquot of the stock solution was mixed with an equal volume of water and incubated at 60 °C. The decrease in psilocin concentration was analyzed from 0 to 6 days using LC-MS.

### Photolysis

For light exposure, an aliquot of the stock solution was mixed with an equal amount of water and exposed to 12 900 lx for 15 days using a photoreactor.^55^ The decrease in psilocin concentration was analyzed from 0 to 15 days using LC-MS. A control sample in dark was also analyzed.

### Chromatographic Analysis of Stressed Samples

Prior to analysis, an aliquot was withdrawn and mixed with an equal volume of IS stock solution adjusting the concentration to 1.0 mg/mL freebase psilocin equivalents. Samples (3 uL) were taken periodically and analyzed via HPLC (0–40% MeCN over 3 min) and the eluent was monitored at 214 nm. A calibration curve for psilocin HCl was established to quantify changes in psilocin concentration (Figure S18).

### Normalization of Outliers

During our analysis, we encountered an issue where the V-shaped sample vessel caused insufficient mixing in some samples, leading to abnormally small IS signals and therefore disproportionately large psilocin signals. To investigate this phenomenon, we conducted a control experiment. This involved diluting the IS stock with water and analyzing it using LC-MS, initially without mixing, then progressively increasing the mixing time using a vortex. A control sample was left unmixed to observe the effects of natural diffusion. The conclusion from this control experiment was that natural diffusion takes hours to give a homogeneous sample, or at least 10 s of intense vortex mixing. The control experiment also showed that insufficient mixing led to decrease in the IS signal with the same proportion as the increase of the analyte signal. Due to a lack of material, it was not feasible to repeat the experiment. Based on the control experiment findings, we corrected for the insufficient mixing in some samples by normalizing the IS and analyte values, It is important to note that the observed trends were consistent with non-normalized data.

To identify insufficiently mixed samples, we used a cutoff of IS AUC three standard deviations lower than the mean value. To avoid skewing the standard deviation values, the outliers were excluded from the calculation. For identified samples with in-sufficient mixing, the IS AUC was increased to the median IS AUC, and the psilocin AUC decreased with the same proportion as the IS AUC was increased. After normalization of insufficiently mixed sample values, the concentration of psilocin was recalculated using the calibration curve, psilocin and IS AUC values.

### Kinetic Calculations

The stability of psilocin salts under various stress conditions was quantitatively analyzed by calculating the half-life based on first-order kinetic reactions. The rate constant, k, was derived from the slope between consecutive data points representing the concentration of psilocin over time. For each experimental condition, k values were calculated for intervals between measurement points, typically resulting in two k values per condition per salt. The average of these k values was then used to compute the half-life of the salts using the standard decay formula. Due to the precision of our measurements, the half-life values are reported with one significant figure.

## Supporting Information Available

NMR spectra of tested compounds, Supporting Information and methods, tables for: metabolic stability assay, Cofactor assay, Plasma stability assay, solubility assay, supplementary figures of forced degradation assay, supplementary figures showing pictures of solutions during the forced degradation assay (PDF)
Molecular formula strings (CSV)

## Abbreviations

COCl_2_: oxalyl chloride
Cs_2_CO_3_: cesium carbonate
DMSO: dimethyl sulfoxide
Et_2_O: diethyl ether
EtOH: ethanol
FaSSIF: fasted state simulated intestinal fluid
H_2_O_2_: hydrogen peroxide
HCl: hydrochloric acid
HPLC: high performance liquid chromatography
5-HT: serotonin receptor
IS: internal standard
IV: intravenous
LC-MS: liquid chromatography mass spectrometry
LiAlH_4_: lithium aluminum hydride
M: Molar
MeCN: acetonitrile
MeOH: methanol
2-MeTHF: 2-methyltetrahydrofuran
NaHCO_3_: sodium bicarbonate
NaOH: sodium hydroxide
nd: not determined
NMR: nuclear magnetic resonance
nt: not tested
ppm: parts per million
SO_3_•pyridine complex: sulfur trioxide pyridine complex
STS: Speeter and Anthony synthesis
TBPP: tetrabenzyl pyrophosphate
TFA: trifluoroacetic acid
THF: tetrahydrofuran
TLC: thin layer chromatography
UV: ultraviolet
