# Supplementary material for: Synthesis and In Vitro Profiling of Psilocin Derivatives: Improved Stability and Synthetic Properties
Source: J Med Chem. 2025 Mar 20;68(7):7153–65. doi: 10.1021/acs.jmedchem.4c02612 (PMC11997985; doi:10.1021/acs.jmedchem.4c02612)
Supplement: Supplementary file 1 — jm4c02612_si_001.pdf [file jm4c02612_si_001.pdf]

Supporting information

Synthesis and In Vitro Profiling of Psilocin Derivatives: Improved stability and Synthetic Properties

Julia Eklund[1], Ulf Bremberg*[1], Jessica Larsson[2], Edvard Torkelsson[2], Johan Wennerberg*[2,3], Symantha Zandelin[2], Luke R. Odell*[1]

1. Department of Medicinal Chemistry, Uppsala University, Box-574, SE-751 23 Uppsala, Sweden Uppsala University 2. Red Glead Discovery, Medicon Village, SE-223 81 Lund, Sweden 3. Department of Chemistry, Lund University, SE-221 00 Lund, Sweden.

Table of contents

Materials S2

Experimental procedures S3

Supplementary tables S5

Supplementary figures S9

^{1}H NMR and ^{13}C NMR for compounds **4a-k**, **5**, **6**, **8**, **9a-f** S16

Representative LCMS S36

References S42

Materials

Materials for experiments described in the supporting information (metabolic stability, plasma stability)

Instrumentation LC-MS/MS

Autosampler	Shimadzu NexeraXR SIL-20AC
Degasser	Shimadzu FCV-11AL
Gradient LC-pumps	Two Shimadzu NexeraXR LC-20AD pumps
Oven Shimadzu	CTO-20AC
System controller	Shimadzu CBM-20A
Mass spectrometer	Sciex QTRAP4500 with Analyst software 1.6.3
MS-probe	Turbospray ESI+

Analytical parameters

Analytical Flow	0.3 mL/min
Analytical column	Phenomenex LUNA C18 Polar, 1.6 µm, 2.1x50mm
Injection volume	10 µL
Column temperature	+40°C
Mobile phase A	H2O/MeCN/Formic acid 95/5/0.1
Mobile phase B	H2O/MeCN/Formic acid 5/95/0.1
MS detection	MRM; ESI Positive mode

Chemicals

Human plasma Innovative research, Pooled Human plasma, K2 EDTA, IPLAK2E100ML-32215, Storage at -20°C, Expiration date: 06/16/2023

Human microsomes	Thermo Fisher Scientific, Catalogue number HMMCPL, batch PL050G-B
Acetonitrile (MeCN)	LiChrosolv, hypergrade for LC-MS, Merck Chemicals
Ethanol	Solveco, 99.7% Absolute, Spectrographic
Formic acid (HCOOH)	For LC-MS, Carlo-Erba
Water	> 18 MΩcm quality (Milli-Q, Millipore)

Table S1 Gradient conditions for compounds with the exception of psilocybin

Time (min)	Mobile phase B%	Curve
0.0	5	linear
4.0	95	
4.5	95	linear
4.6	5	
6.0	5	Eq

Table S2 Gradient conditions for psilocybin

Time (min)	Mobile phase B%	Curve
0.0	0	linear
0.5	0	
4.0	95	
4.5	95	linear
4.6	0	
6.0	0	Eq

Materials for experiments described in the supporting information (solubility)

Chemicals

Dimethylsulfoxide (DMSO) CHROMASOLV™ Plus, >99.7%
Acetonitrile (MeCN) LiChrosolv, hypergrade for LC-MS, Merck Chemicals
Formic acid (HCOOH) For LC-MS, Carlo-Erba
Water > 18MΩcm quality (Milli-Q, Millipore
Di-sodium-hydrogenphoshate dihydrate Merck
Sodium-dihydrogenphosphate monohydrate Merck

Instrumentation

Autosampler Agilent 1260 Multisampler, G7167A
Gradient LC-pump Agilent 1260 Flexible Pump, G7104C
UV-detector Agilent 1260 DAD HS, G7117C
Column oven Agilent 1260 MCT, G7116A

Analytical parameters

Analytical Flow 0.5 mL/min
Analytical column Luna® Omega 1.6 µm Polar C18 100Å, 50 x 2.1 mm
Injection volume 3.0 µL (15 µL for "high" injection of buffer sample)
Column temperature +40°C
Mobile phase A H2O/MeCN/Formic acid 95/5/0.1
Mobile phase B H2O/MeCN/Formic acid 5/95/0.1

Materials and analytical parameters for FT-ICR-MALDI-HRMS

The high-resolution mass spectrometry (HR-MS) experiments were performed using a MALDI-Fourier-transform ion cyclotron resonance (FTICR) (7T solariX XR-2ω, Bruker Daltonics) mass spectrometer equipped with a Smartbeam II 2 kHz laser. Briefly, a stock solution (1mg/ml) of the compound 5 was prepared and mixed 1:1 with a matrix solution containing DHB (40 mg/ml) in 50% ACN (0.02% TFA). 1 µl of the mixed solution was spotted onto MPTP MALDI target plate (Bruker Daltonics) and dried at room temperature. The instrument was tuned for optimal detection of small molecules (m/z 86–500) in positive polarity using the quadrature phase detection (QPD) (2ω) mode. The method was calibrated with red phosphorus over the mass range and DHB matrix ion [2M–2H2O+H]+ m/z 273.039364 was used as lock mass. The compound was identified with sub ppm mass accuracy and specific isotopic pattern of boron in its molecular structure.

Experimental procedures

Plasma stability

Incubation samples. 10 mM stock solutions of test compounds in DMSO were used to prepare 100 µM solutions by dilution in in EtOH/water 25/75 (v/v). 6 µL of the 100 µM solution was spiked into 594 µL pre-warmed plasma at 37 °C giving a final concentration of 1 µM. The incubation tube was then mixed on a vortex. A sample (60 µL) was taken and added to an Eppendorf tube containing 180 µL acetonitrile. The tube was thoroughly mixed with a vortex. The precipitated sample was then treated with centrifugation (10.000 g, 10 minutes, 18 °C), 150 µL of the supernatant was mixed with 450 µL water directly into a LC-vial (glass). This step was repeated for every time point. The first time point was approximately taken 15-20 seconds after spiking. Other timepoints were 5,10, 30, 60, 120, 240 minutes. **Pre-precipitated samples.** 594 µL of pre-warmed plasma was precipitated with 1800 µL ACN. 6 µL of the 100 µM solution was spiked into the precipitated plasma. 1 mL was removed and added to an Eppendorf tube. After a centrifugation step (10.000 g, 10 minutes, 18 °C), 150 µL of the supernatant was mixed with 450 µL water directly into a LC-vial (glass).

Metabolic stability in human microsomes

The assay was run at 37°C with a microsomal protein concentration of 0.5 mg/mL and a compound concentration of 1 µM in a 0.1 M potassium phosphate buffer at pH 7.4. The compounds were added to the microsome suspension whereafter the enzymatic reaction was initiated by the addition of the co-factor NADPH, giving a final NADPH concentration of 1 mM (Table 9). Samples

at six time points (0, 5, 10, 15, 25, and 45 minutes) were withdrawn from the incubation and the enzymatic reaction was terminated by protein precipitation with an equal volume of acetonitrile. After a centrifugation step, an aliquot of the resulting supernatant was mixed with three aliquots of milli Q water before analysis with tandem mass spectrometry (LC-MS/MS). The human microsomal stability assay includes two positive reference compounds, verapamil and dextromethorphan.

Solubility

For each compound, 6 µL of 10 mM stock solutions were added to 594 µL 100 mM phosphate buffer, pH 7.4 to a nominal concentration of 100 µM. The mixture was rigorous mixed at room temperature for 20-24 hours. Prior to HPLC-UV analysis the mixture was filtered through a Millex – LH, 0.45 µm. The concentration of the filtrated samples was determined by comparison with a 100 µM standard sample in DMSO. The exact mass for the compound was verified by single-quad MS. No results can be reported if purity for the standard sample is lower than 80%. . The reported range for this assay is from 0.1 to 100 µM.

Solid solubility assay (compound 8): After addition of 100 mM phosphate buffer, pH 7.4, to dry substance, the samples were agitated for 24 hours. The samples were subsequently filtered, diluted and analysed by HPLC-UV. Samples were quantified by comparison with a three-point standard curve (1-100 µM).

Stability study in FaSSIF supplemented with esterase

The stability of the synthesized psilocin ester prodrugs was evaluated under simulated gastrointestinal (GI) conditions using Fasted State Simulated Intestinal Fluid (FaSSIF) supplemented with esterase. The concentration of the ester prodrugs was set at 0.292 mM, which corresponds to their predicted concentration in the human stomach following oral administration, based on a biologically equivalent dose of psilocybin (25 mg). This concentration was calculated assuming a fasting gastric fluid volume of 50 mL, plus an additional 250 mL to account for the intake of a glass of water, resulting in a total gastric volume of 300 mL.[1]

FaSSIF was prepared from 3F powder and FaSSIF buffer concentrate (Biorelevant) according to protocol from the manufacturer.[2] DMSO stock solutions of the prodrugs were diluted in FaSSIF to yield a concentration of 0.365 mM. For each experiment, 80 µL of the diluted stock solution was transferred to an Eppendorf tube and pre-incubated at 37°C for 5 minutes. The reaction was initiated by adding 20 µL of pre-incubated porcine liver esterase (MedChemExpress) (100 IU/mL) dissolved in FaSSIF, resulting in a final esterase activity of 20 IU/mL and a final prodrug concentration of 0.292 mM. The reaction mixture was vigorously mixed and incubated at 37°C. After one minute, the enzymatic activity was terminated by the addition of 100 µL ice-cold acetonitrile containing 0.5% (v/v) formic acid. The samples were then vortex-mixed for 1 minute, followed by centrifugation at 5000 rpm for 5 minutes. A control sample without esterase was prepared in the same manner. The supernatant was analyzed using LC-MS to quantify the extent of prodrug degradation.

Rationale for Enzyme Concentration and Experimental Conditions. The enzymatic activity in the experiments was set at 20 IU/mL to avoid the rapid hydrolysis that would occur at physiological esterase levels in the stomach, which range from 270 to 4900 IU/mL in the fasted state.[3] This lower enzyme concentration was chosen to allow for the accurate measurement of the prodrug degradation rates, by leaving a quantifiable amount of prodrug after 1 minute.

Criteria for prodrug conversion rate. Previous studies have demonstrated that serum drug concentrations can increase rapidly, as early as 10 minutes after ingestion.[4] Gastric emptying half-life has been estimated to be approximately 11-15 minutes.[1] To account for these conditions and create a conservative "safety window," we established a criterion where only 0.1% of the prodrug would remain after 5 minutes, based on regulatory requirements to have <0.1% of any specific impurity in a GMP manufactured drug. This target would ensure that the prodrug is almost completely converted to psilocin well before entering systemic circulation. The required half-life to achieve this level of degradation was calculated by solving for $t_{½}$ using the formula for exponential decay:

$$t_{\frac{1}{2}} = \frac{t}{log_2\left(\frac{1}{0.001}\right)}$$

For the prodrug concentration to decrease to 0.1% of its initial level within 5 minutes, the required half-life was calculated to be < 0.5 minutes.

Supplementary tables

Table S3 Evaluation of metabolic stability in human microsomes

No.	Metabolic stability (µL/min/mg protein)	Comment
**3**	43/44	
**4a**	>200	Rapid degradation
**4b**	>200	Rapid degradation
**4c**	>200	Rapid degradation
**4d**	>200	Rapid degradation
**4e**	>200	Rapid degradation
**4f**	>200	Rapid degradation
**4g**	>200	Rapid degradation
**4h**	>200	Rapid degradation
**4i**	>200	Rapid degradation
**4j**	>200	Rapid degradation
**4k**	<10/<10	Low retention in chromatography
**5**	>200	Rapid degradation, problem finding precursor/product transition.
**6**	>200	Rapid degradation
**8**	<10/<10	Low retention in chromatography.

Table S4 Results from the modified microsome assay where degradation with and without the addition of NADPH was compared

No.	Co-factor experiment
**3**	No degradation without co-factor
**4a**	Degradation without co-factor
**4b**	No degradation without co-factor
**4c**	No degradation without co-factor
**4d**	Very low peak area for the first time point, instability suspected
**4e**	Very low peak area for the first time point, instability suspected
**4f**	Very low peak area for the first time point, instability suspected
**4g**	Degradation without co-factor
**4h**	Degradation without co-factor
**4i**	Degradation without co-factor
**4j**	Degradation without co-factor
**4k**	Not tested
**5**	Very low peak area for the first time point, instability suspected
**6**	No degradation without co-factor
**8**	No degradation without co-factor

Co-factor experiment. In order to determine if the low response and rapid degradation seen in the microsome media was due to metabolism by the microsomes (consisting mainly of cytochrome P450 enzymes) or if it was due to other factors, the compounds (with the exception of 4k) were incubated in the microsome suspension both with and without the addition of NADPH. Samples

were taken at three timepoints, and the degradation was compared to determine if it was dependent on NADPH. Compound 4k was not analyzed as it was found to be stable in the microsome assay as well as providing a high response in the analysis (Table S4). Compounds **4a, 4g**, **4h**, **4i** & **4j** were found to give intrinsic clearance values > 200 µL/min/mg protein both with and without the addition of NADPH. This suggests that the degradation is not dependent on the major enzyme groups found in the microsomes that need to be activated by a co-factor.

Table S5 Percent of peak area found in microsome suspension 15 seconds after the addition of NADPH in comparison with the same amount dissolved in H2O/EtOH

No.	Area at 0.25 min. in microsomes/NADPH suspension	Area in solution (125 nM)	% found in microsome suspension
**3**	3000000	3390000	88.5
**4a**	850	3690000	0.0
**4b**	9485	3340000	0.3
**4c**	5900	3580000	0.2
**4d**	2063	3420000	0.1
**4e**	194	2110000	0.0
**4f**	3635	1610000	0.2
**4g**	1155000	1980000	58
**4h**	1920000	4400000	44
**4i**	253750	3550000	7.1
**4j**	588500	1320000	45
**4k**	3157500	3990000	79
**5**	56025	77400	72
**6**	213500	1440000	15
**8**	104325	128000	81.5

Comparison with compounds in solution. A comparison was made between the peak area of the compounds in solution with that obtained in the microsome suspension directly after the addition of NADPH (about 0.25 minutes) (Table S5). Before the addition of NADPH, the compounds are equilibrated in the microsome suspension for approximately five minutes. All compounds were prepared in solution by diluting a portion of the DMSO solutions with H2O/EtOH (75:25) to 125 nM (the final concentration of compounds for analysis in the microsome assay).

For the compounds where no results from the microsome assay could be reported due to low peak area (**4d, 4e, 4f** & **5)**, less than one percent of the expected peak area as compared with the area in solution was detected. This suggests that the compounds degrade rapidly before the addition of the co-factor after five minutes in incubation. For compounds **4g**, **4h**, **4i** & **4j**, which were found to give an acceptable response for the initial time points in the microsome assay followed by rapid degradation, somewhat higher relative peak areas were found.
It is not clear why compound **6**, which was found to give a low clearance value in the microsome assay and no degradation without NADPH was only found at 15% of the area seen in solution in the microsome suspension.

Table S6 Time-dependent decline in concentration of five compounds for in vitro plasma stability in human plasma and calculated half-life

	% REMAINING							
**Time (min)**	0.2	5	10	30	60	120	240	**Half life**
**3**	100	103	103	105	111	110	107	> 8 h
**4a**	100	0.2	0.2	0.2	0.2	0.1	0.2	< 5 min
**4g**	100	1.5	0.1	0.0	0.0	0.0	0.0	< 5 min
**4i**	100	0.0	0.0	0.0	0.0	0.0	0.0	< 5 min
**8**	100	135	124	130	122	127	122	> 8 h

Plasma stability. The compounds were incubated separately at a concentration of 1 µM in +37°C pre-warmed pooled human plasma from commercial resources. The incubations were performed over a four-hour period with the first sample taken after addition of the compounds to plasma followed by a thorough mixing, with further sampling at 5, 10, and 30 minutes as well as 1, 2, and 4 hours. The samples were analyzed by LC-MS/MS using relative peak areas for the calculations of the time-dependent decline in concentration and half-life as seen in Table S6.

Table S7 Comparison of peak area in incubation plasma after addition and mixing with pre-precipitated plasma.

No.	Peak area in incubation plasma (0.2 min)	Peak area with pre-precipitated plasma	% found in incubation plasma at time 0.2 min
**3**	942500	821000	115
**4a**	86300	884000	9.8
**4g**	401500	444000	90
**4i**	225000	1290000	17
**8**	35550	53200	67

Test of compounds in pre-precipitated plasma. An additional experiment was conducted by adding the compounds to pre-precipitated plasma (see methods) in order to determine if degradation occurred in inactive plasma. The results in Table S7 suggest that some of the compounds (**4a** and **4i**) were heavily degraded already in the first incubation sample taken 0.2 minutes after the addition of the compound. The compounds which were found to degrade most rapidly in the incubation plasma had degraded substantially in the 0.2 minutes sample in which the vials were only mixed before the precipitation was carried out. Compound **3** did not present any degradation in any of the experiments while the reference compound **8** seems to be stable in plasma in vitro for more than 8 hours, but did show some degradation in the first sample point at time 0.2 min. The difference is slightly higher than the normal acceptable analytical variation but may still have an analytical origin as the compound gives poor chromatographic properties for reversed-phase LC conditions.

Table S8 Determined solubility in 100 mM phosphate buffer, pH 7.4, 1% DMSO.

No.	Determined solubility (µM)
**3**	>90
**4a**	77
**4b**	87
**4c**	87
**4d**	>90
**4e**	*
**4f**	*
**4g**	>90
**4h**	>90

**4i**	88
**4j**	*
**4k**	>90
**5**	*
**6**	>90
**8**	>1000**

*Solubility can't be determined due to degradation/impurity present in samples of DMSO standard or filtrated buffer sample. Though, the observed data indicate a high solubility in this assay of probably >90 µM..

**Results obtained with solid solubility assay as the sample co-eluted with DMSO.

Solubility. We encountered challenges in determining the solubility for several compounds, primarily due to degradation both in the standard samples and the filtered buffer samples. Despite these complications, the data obtained suggest a high solubility, potentially in excess of 90 µM. In the instances where we could produce reliable results, all compounds demonstrated commendable solubility, further supporting this assumption. Solubility data for Psilocybin (compound 8) indicate values >1000 µM, which represents the upper limit of the assay.

Table S9 Salt configuration and yield before/after salt precipitation.

	Assumption 1 psilocin ester proton = 1 integral								
**Sample id**	**Formiate int. (11 ppm)**	**Fumarate int. (6.5 ppm)**	**Salt Mw (g/mol)**	**Amt. recov. as salt (mg)**	**Parent mol. Mw (g/mol)**	**Parent mol. Formula**	**Parent mol. amt. (mg)**	**Yield (%)**	**Yield bf. salt prec. (%)**
3	0	0	294,4	-	294,40	$C_{19}H_{22}N_2O$	8560,0	84%	-
4a	0,97	3,11	470,5	33,8	246,31	C14H18N2O2	17,7	14%	45%
4b	0,94	2	432,7	38,8	274,36	C16H22N2O2	24,6	18%	59%
4c	0,94	2	446,8	24,3	288,39	C17H24N2O2	15,7	11%	18%
4d	1	0	349,4	41,1	304,39	C17H24N2O3	35,8	23%	-
4e	1,01	5,47	625,2	5,2	262,31	C14H18N2O3	2,2	2%	52%
4f	0,89	21	1597,2	7,5	338,41	C20H22N2O3	1,6	1%	13%
4g	0,93	3,26	503,4	24,9	272,35	C16H20N2O2	13,5	10%	66%
4h	0,95	1	409,2	13,4	308,38	C19H20N2O2	10,1	6%	19%
4i	1,03	2,23	452,1	14,0	276,34	C15H20N2O3	8,6	6%	33%
4j	0,88	18	1456,8	8,1	372,55	C23H36N2O2	2,1	1%	38%
4k	0,95	1	416,2	66,6	315,42	C18H25N3O2	50,5	31%	57%
5	1	1,61	386,5	32,0	248,09	C12H17BN2O3	20,5	16%	-
6	0	0	284,3	-	284,33	C12H16N2O4S	32,2	22%	-
8	0	0	284,3	-	284,25	C12H17N2O4P	21,6	15%	-

Supplementary figures

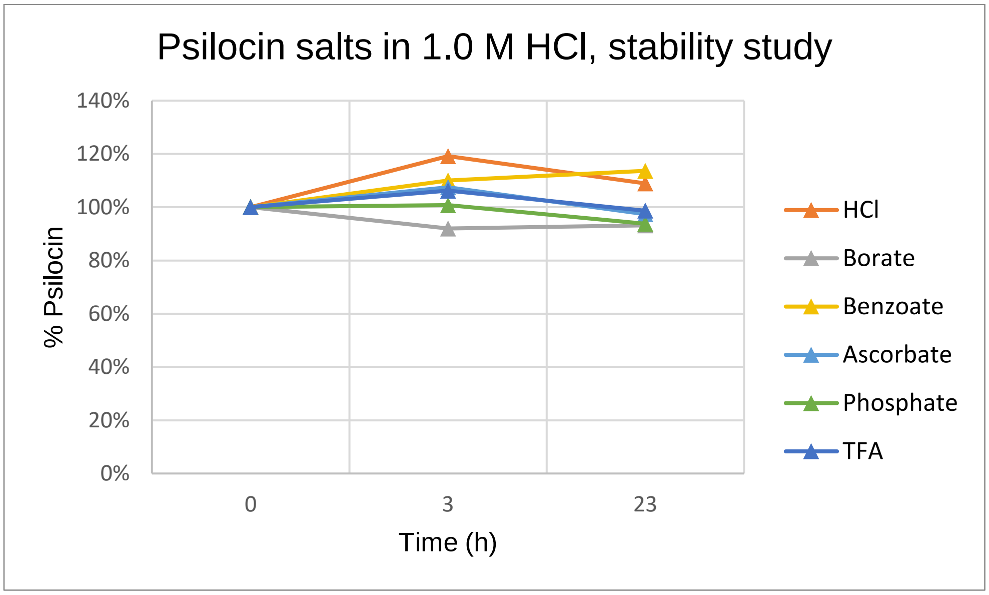

Figure S1 Concentration of various psilocin salts (HCl, Borate, Benzoate, Ascorbate, Phosphate, and TFA) measured at 0, 3, and 23 hours during a forced degradation study in 1.0 M HCl at room temperature. Concentrations were quantified using liquid chromatography-mass spectrometry (LC-MS) relative to an internal standard using a calibration curve.

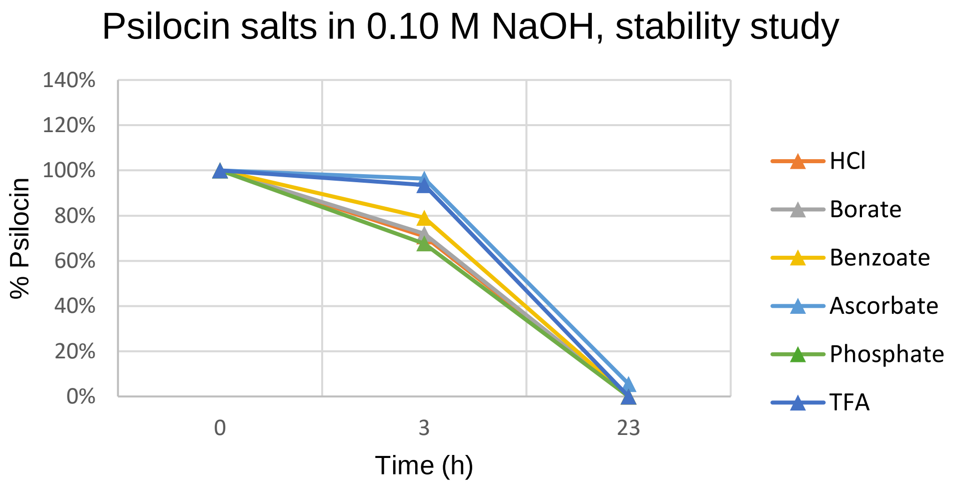

Figure S2 Concentration of various psilocin salts (HCl, Borate, Benzoate, Ascorbate, Phosphate, and TFA) measured at 0, 3, and 23 hours during a forced degradation study in 0.10 M NaOH at room temperature. Concentrations were quantified using liquid chromatography-mass spectrometry (LC-MS) relative to an internal standard using a calibration curve.

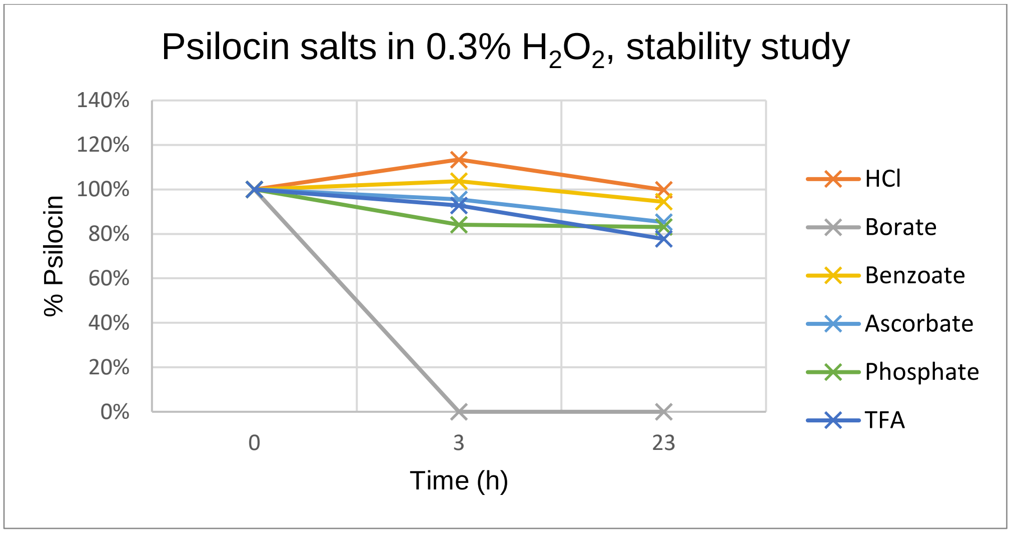

Figure S3 Concentration of various psilocin salts (HCl, Borate, Benzoate, Ascorbate, Phosphate, and TFA) measured at 0, 3, and 23 hours during a forced degradation study in 0.3% H_2O_2 at room temperature. Concentrations were quantified using liquid chromatography-mass spectrometry (LC-MS) relative to an internal standard using a calibration curve.

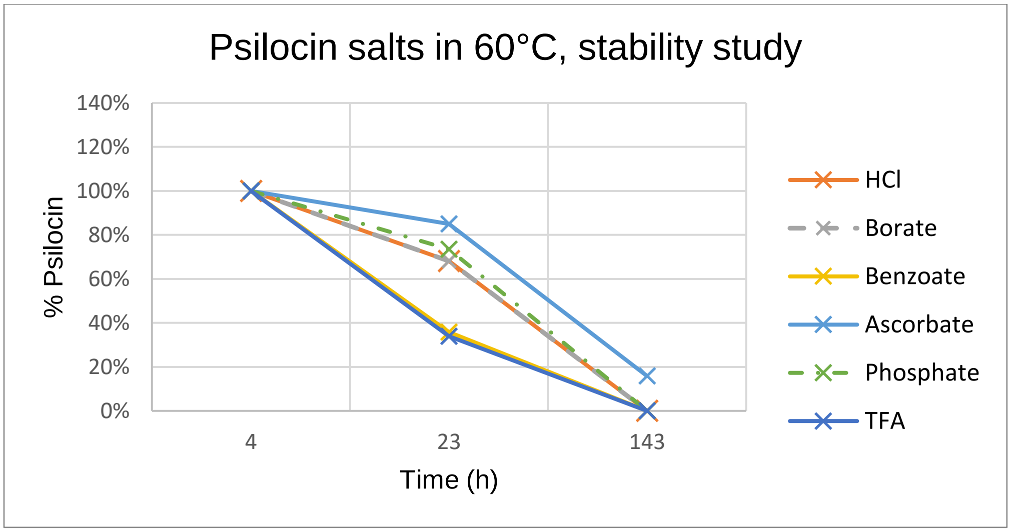

Figure S4 Concentration of various psilocin salts (HCl, Borate, Benzoate, Ascorbate, Phosphate, and TFA) measured at 4, 23, and 143 hours during a forced degradation study in 60°C. Concentrations were quantified using liquid chromatography-mass spectrometry (LC-MS) relative to an internal standard using a calibration curve.

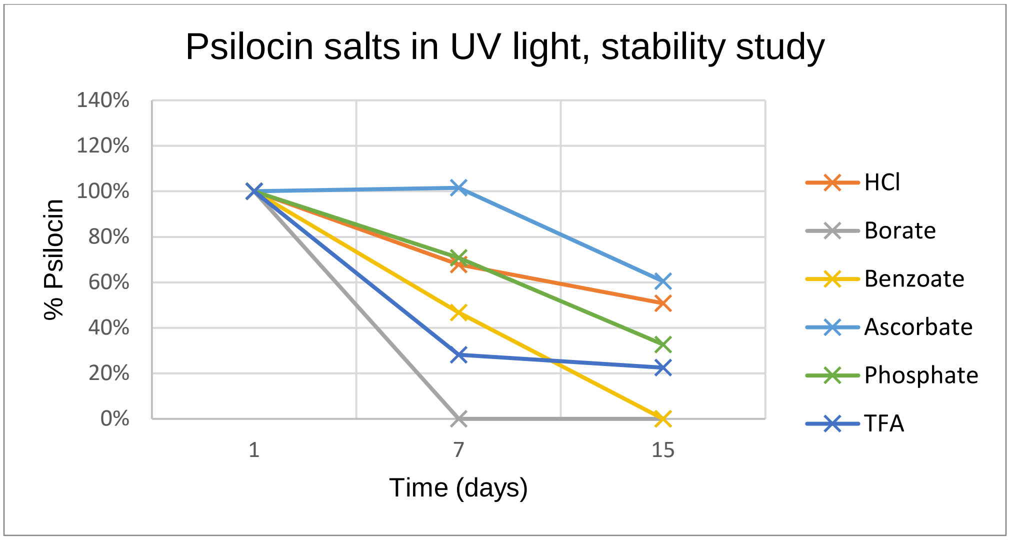

Figure S5 Concentration of various psilocin salts (HCl, Borate, Benzoate, Ascorbate, Phosphate, and TFA) measured at 1, 7, and 15 days during a forced degradation study (in water:acetonitrile, 1:1) in UV light (4 broad spectrum led-strips XH-B01020 (3200 K, 2 W) and 16 16 LED-strips XH-B01020 (465 nm, 2 W), 12900 lux measured at test vial, distance 10 cm, at room temperature. Concentrations were quantified using liquid chromatography-mass spectrometry (LC-MS) relative to an internal standard using a calibration curve.

Rerun of ascorbic salt performed in triplicates

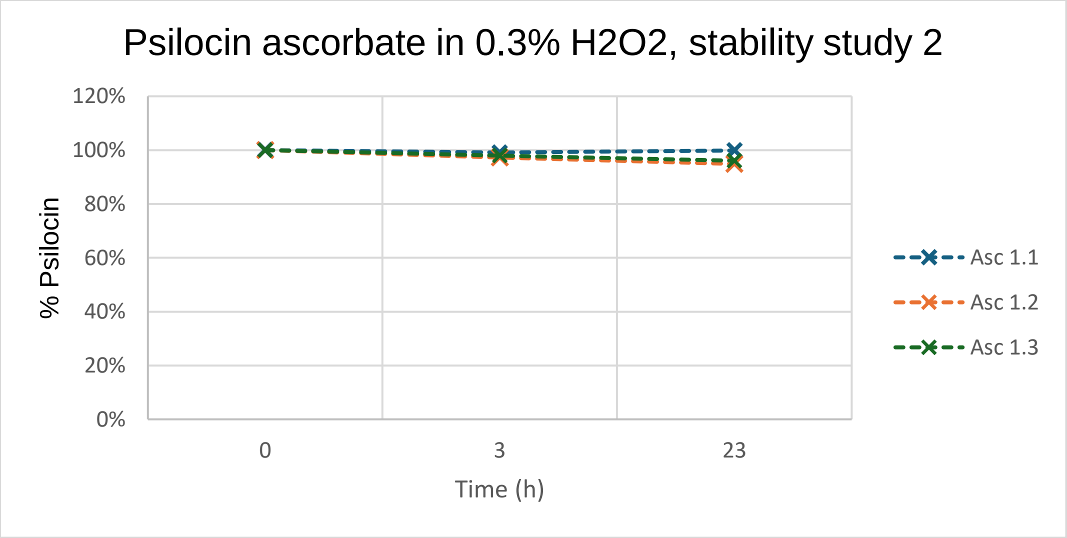

Figure S6 Concentration of the ascorbic salt measured at 0, 3, and 23 hours during a forced degradation study in 0.3% H_2O_2 at room temperature. Concentrations were quantified using liquid chromatography-mass spectrometry (LC-MS) relative to an internal standard using a calibration curve.

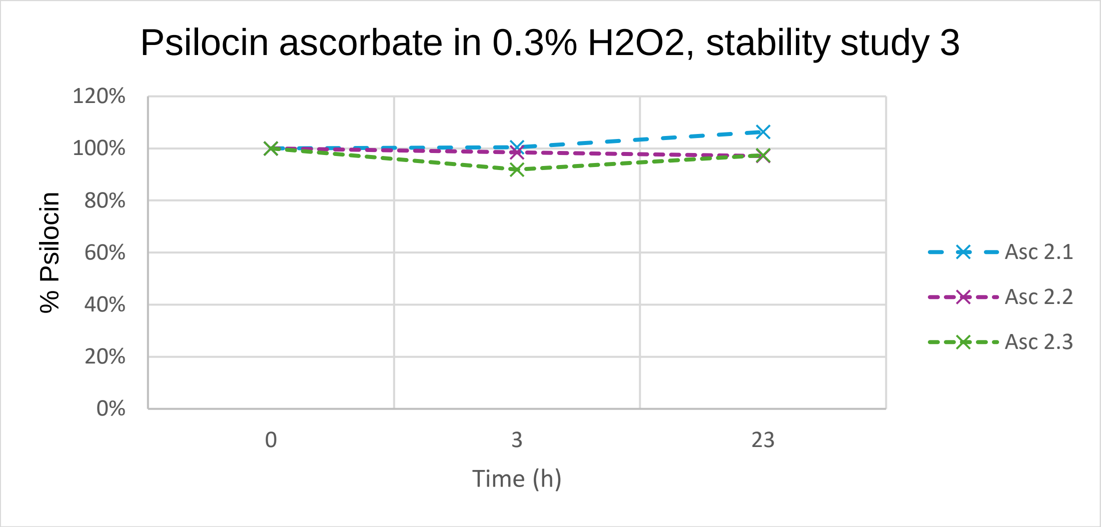

Figure S7 Concentration of the ascorbic salt measured at 0, 3, and 23 hours during a forced degradation study in 0.3% H_2O_2 *at room temperature. Concentrations were quantified using liquid chromatography-mass spectrometry (LC-MS) relative to an internal standard using a calibration curve.*

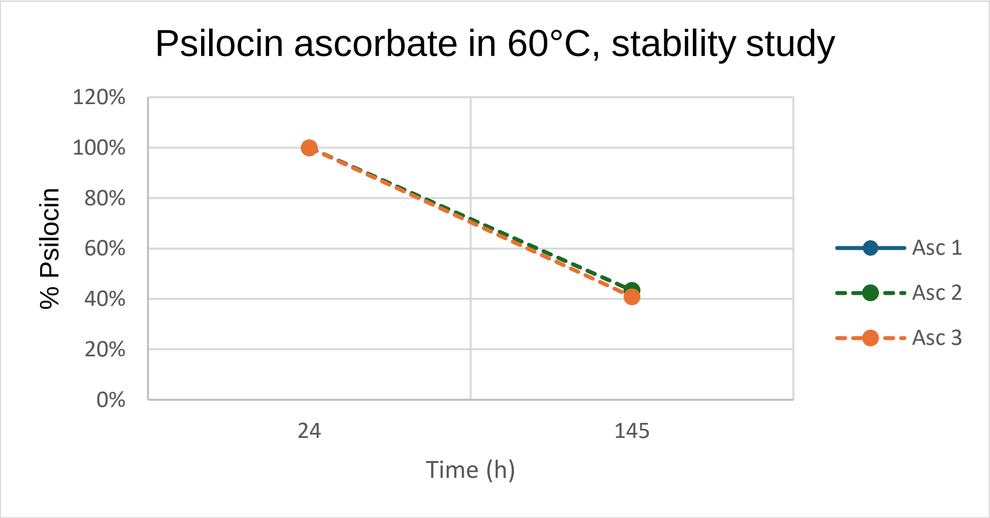

Figure S8 Concentration of the ascorbic salt measured at 24, and 145 hours during a forced degradation study in 60°C. Concentrations were quantified using liquid chromatography-mass spectrometry (LC-MS) relative to an internal standard using a calibration curve.

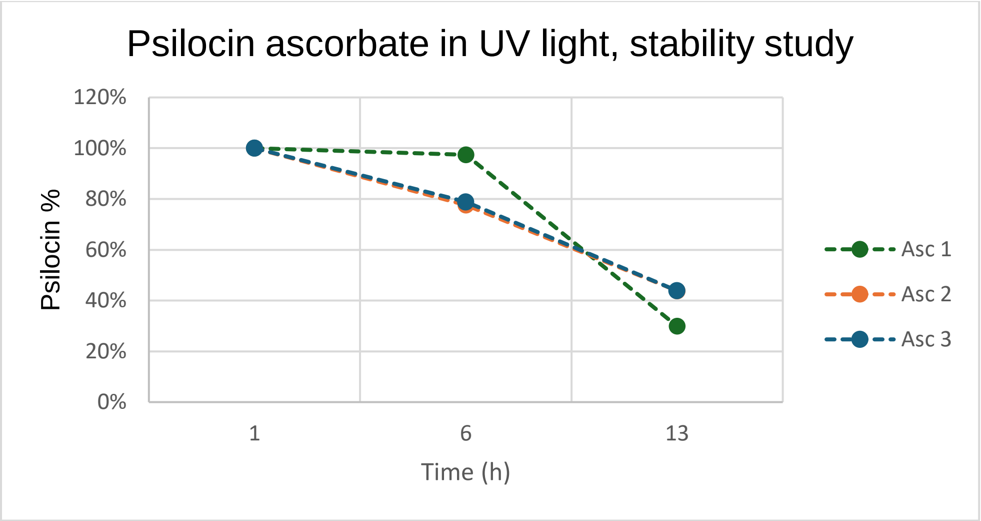

Figure S9 Concentration of the ascorbic salt measured at 1, 6, and 13 days during a forced degradation study (in water:acetonitrile, 1:1) in UV light (4 broad spectrum led-strips XH-B01020 (3200 K, 2 W) and 16 16 LED-strips XH-B01020 (465 nm, 2 W), 12900 lux measured at test vial, distance 10 cm, at room temperature. Concentrations were quantified using liquid chromatography-mass spectrometry (LC-MS) relative to an internal standard using a calibration curve.

Photos of vials during forced degradation of psilocin salts

Since it is known that psilocin darkens upon degradation,[5] photos of the vials were taken to follow the degradation process visually.

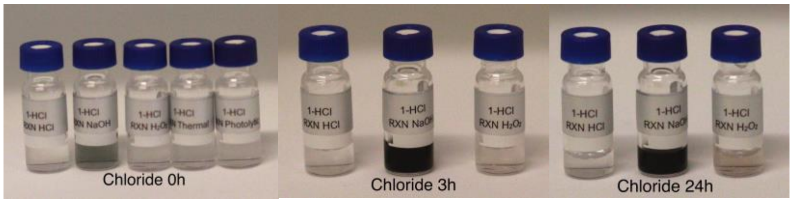

Figure S10 Pictures of the HCl salt during forced degradation experiments. Left: all conditions at 0h. Middle: 1 M HCl, 0.1 M NaOH & 0.3% H_2O_2 at 3h. Right: 1 M HCl, 0.1 M NaOH & 0.3% H_2O_2 at 24h.

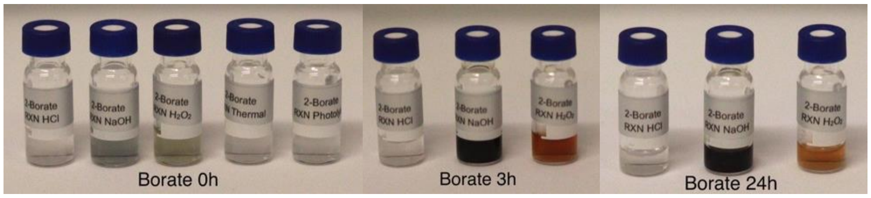

Figure S11 Pictures of the borate salt during forced degradation experiments. Left: all conditions at 0h. Middle: 1 M HCl, 0.1 M NaOH & 0.3% H_2O_2 at 3h. Right: 1 M HCl, 0.1 M NaOH & 0.3% H_2O_2 at 24h.

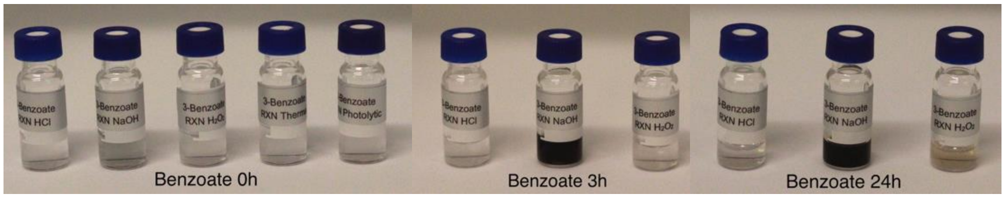

Figure S12 Pictures of the benzoic salt during forced degradation experiments. Left: all conditions at 0h. Middle: 1 M HCl, 0.1 M NaOH & 0.3% H_2O_2 at 3h. Right: 1 M HCl, 0.1 M NaOH & 0.3% H_2O_2 at 24h.

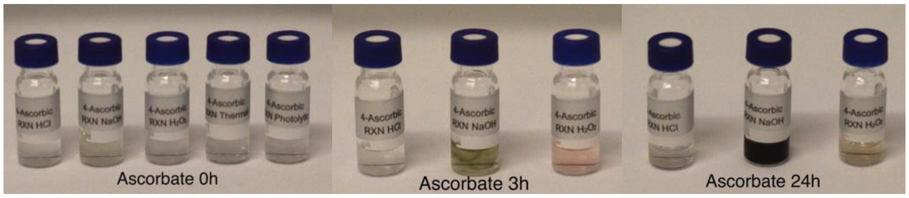

Figure S13 Pictures of the ascorbic salt during forced degradation experiments. Left: all conditions at 0h. Middle: 1 M HCl, 0.1 M NaOH & 0.3% H_2O_2 at 3h. Right: 1 M HCl, 0.1 M NaOH & 0.3% H_2O_2 at 24h.

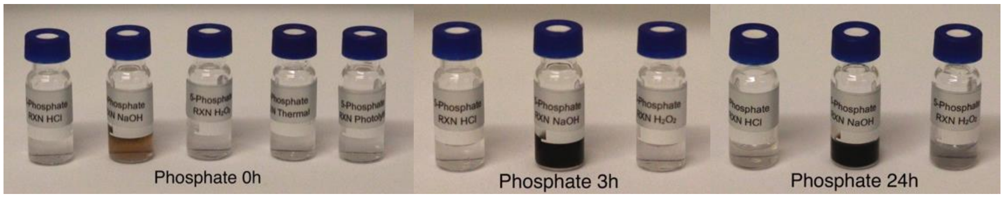

Figure S14 Pictures of the phosphoric salt during forced degradation experiments. Left: all conditions at 0h. Middle: 1 M HCl, 0.1 M NaOH & 0.3% H_2O_2 at 3h. Right: 1 M HCl, 0.1 M NaOH & 0.3% H_2O_2 at 24h.

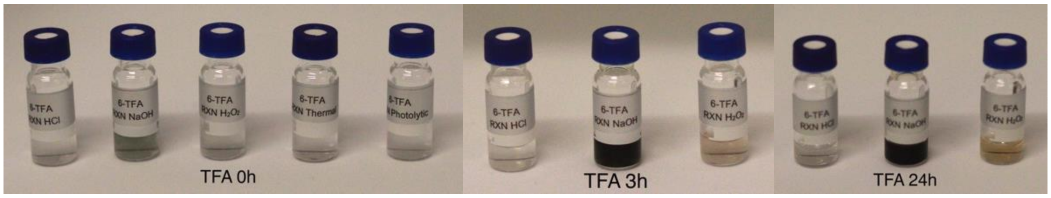

Figure S15 Pictures of the TFA salt during forced degradation experiments. Left: all conditions at 0h. Middle: 1 M HCl, 0.1 M NaOH & 0.3% H_2O_2 at 3h. Right: 1 M HCl, 0.1 M NaOH & 0.3% H_2O_2 at 24h.

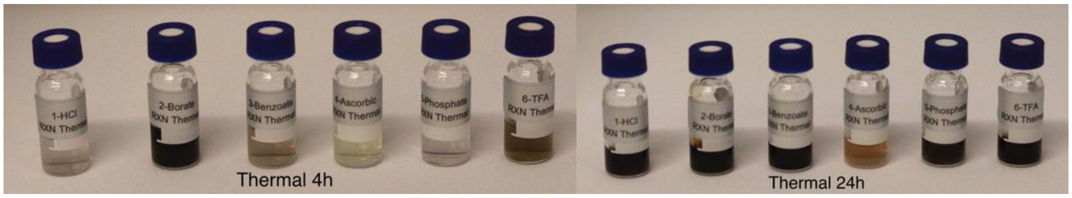

Figure S16 Pictures of the various salts during thermal degradation experiments. Left: thermal at 4h. Right: thermal at 24h.

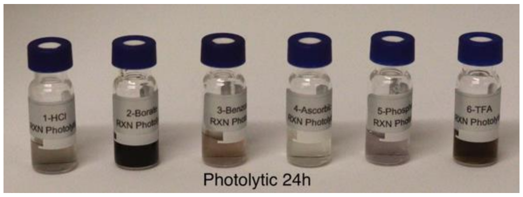

Figure S17 Pictures of the various salts during photolytic degradation experiments at 24h.

Preparation of the calibration curve. 2.47 mg of psilocin HCl was dissolved in 1500 µL acetonitrile:deionized water mixture 1/1 (v/v). An aliquot was withdrawn and diluted with equal volume of IS stock solution. The resulting solution containing psilocin HCl (1.0 mg/mL) and IS stock (2.5 mM, 0.40 mg/mL) was diluted with deionized water to achieve concentrations in the range of 1.0–0.1 mg/mL, which were subjected to chromatographic analysis. The resulting peak areas (AUC), which reflect the quantity of psilocin, were plotted on the x-axis against the corresponding concentrations on the y-axis. The concentration of psilocin was quantified relative to the internal standard. The psilocin AUC had a non-linear relationship to the concentration, most likely due to adsorption to HPLC vial plastic vessel walls. To accurately define the relationship between concentration and peak area, a non-linear regression analysis was conducted. This analysis generated a trend line and a regression equation of the type y = ax^b, which was used to calculate psilocin concentrations in the analyzed samples.

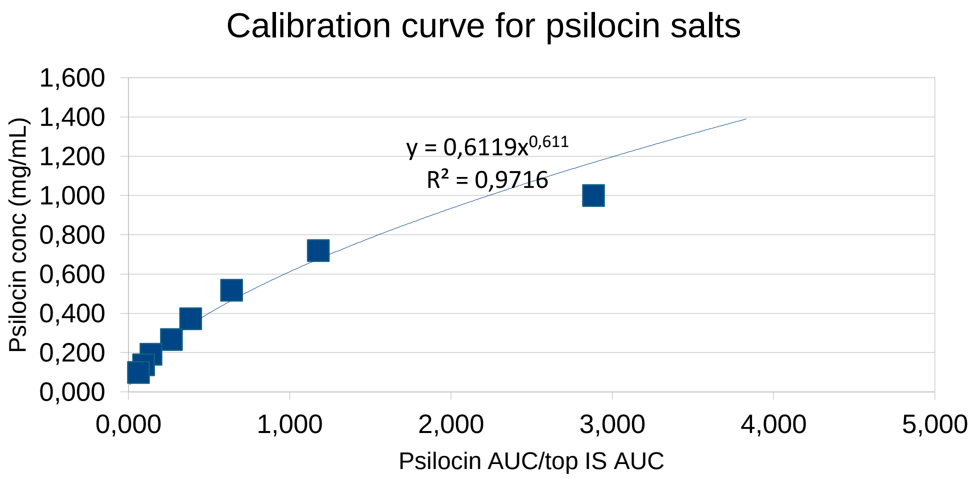
Calibration curve for psilocin salts
1,600
1,400
1,200
1,000
0,800
0,600
0,400
0,200
0,000
Psilocin conc (mg/mL)
y = 0,6119x^0,611
R² = 0,9716
0,000
1,000
2,000
3,000
4,000
5,000
Psilocin AUC/top IS AUC

Figure S18

^{1}H NMR and ^{13}C NMR for compounds **4a-k**, **5**, **6**, **8**, **9a-f**

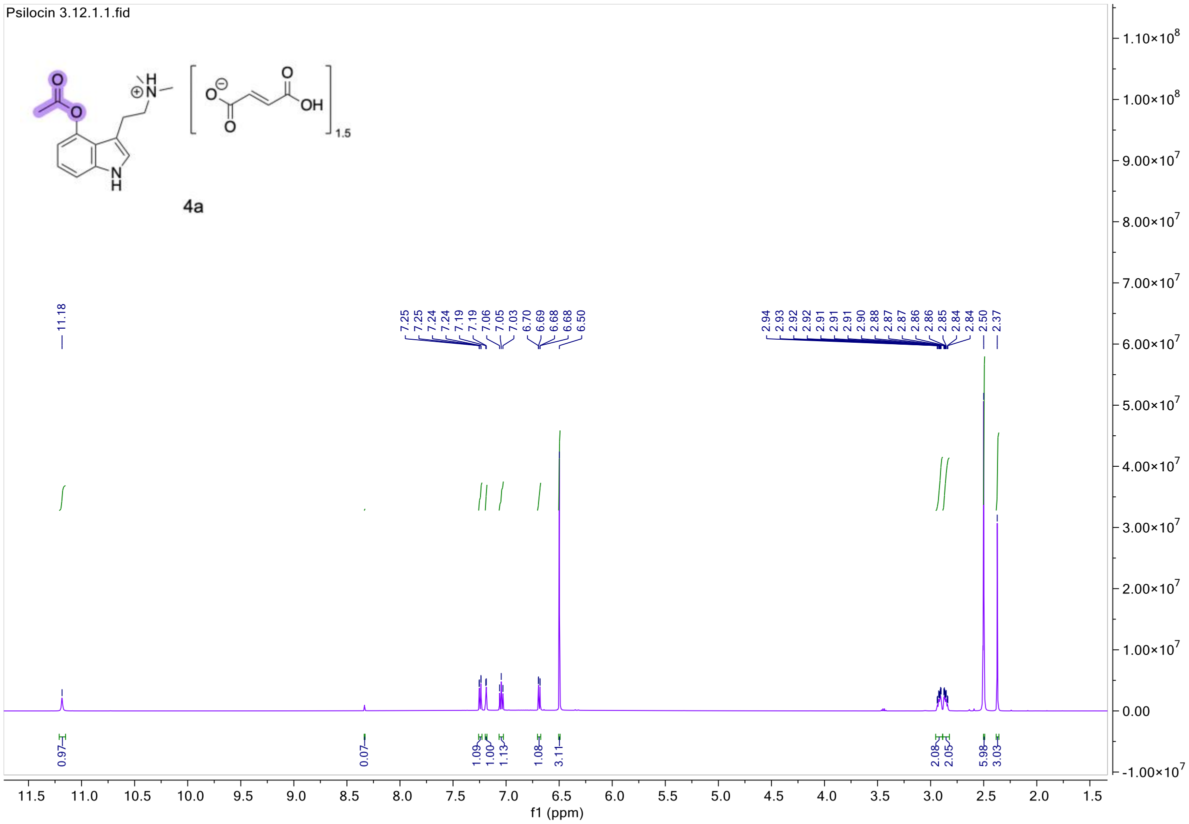

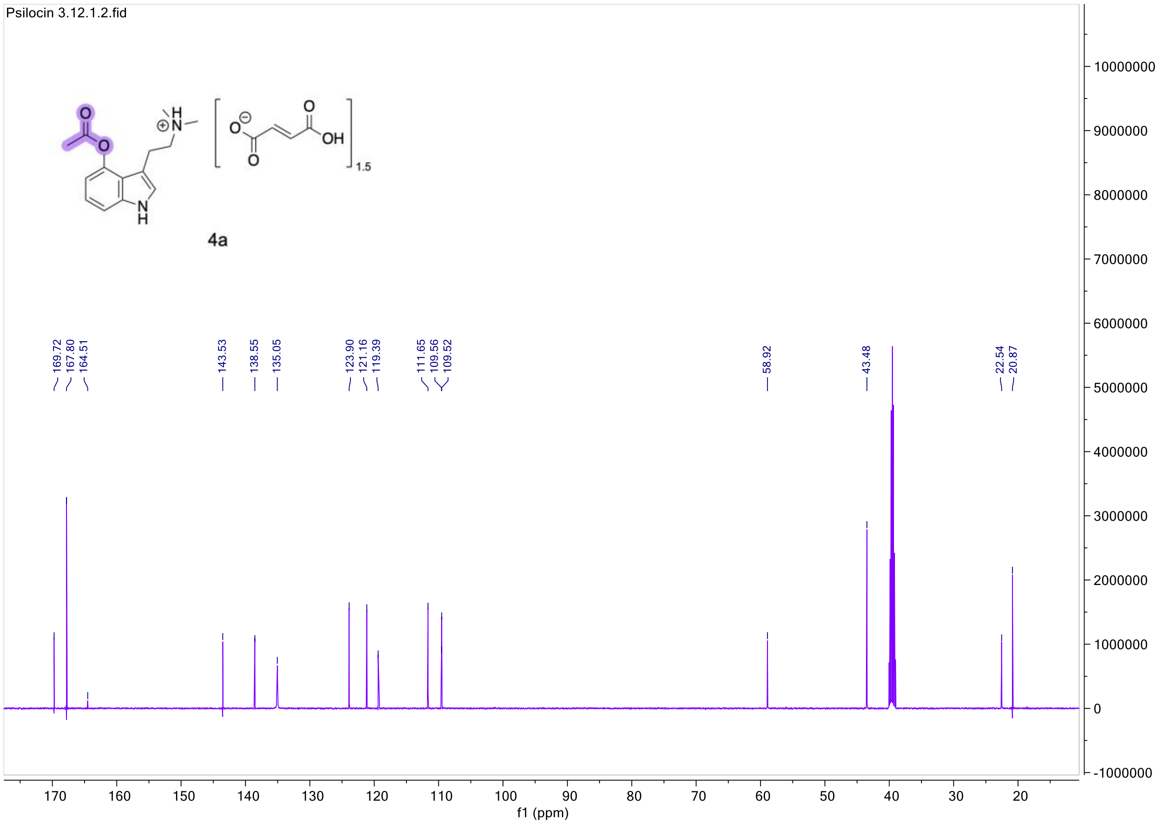

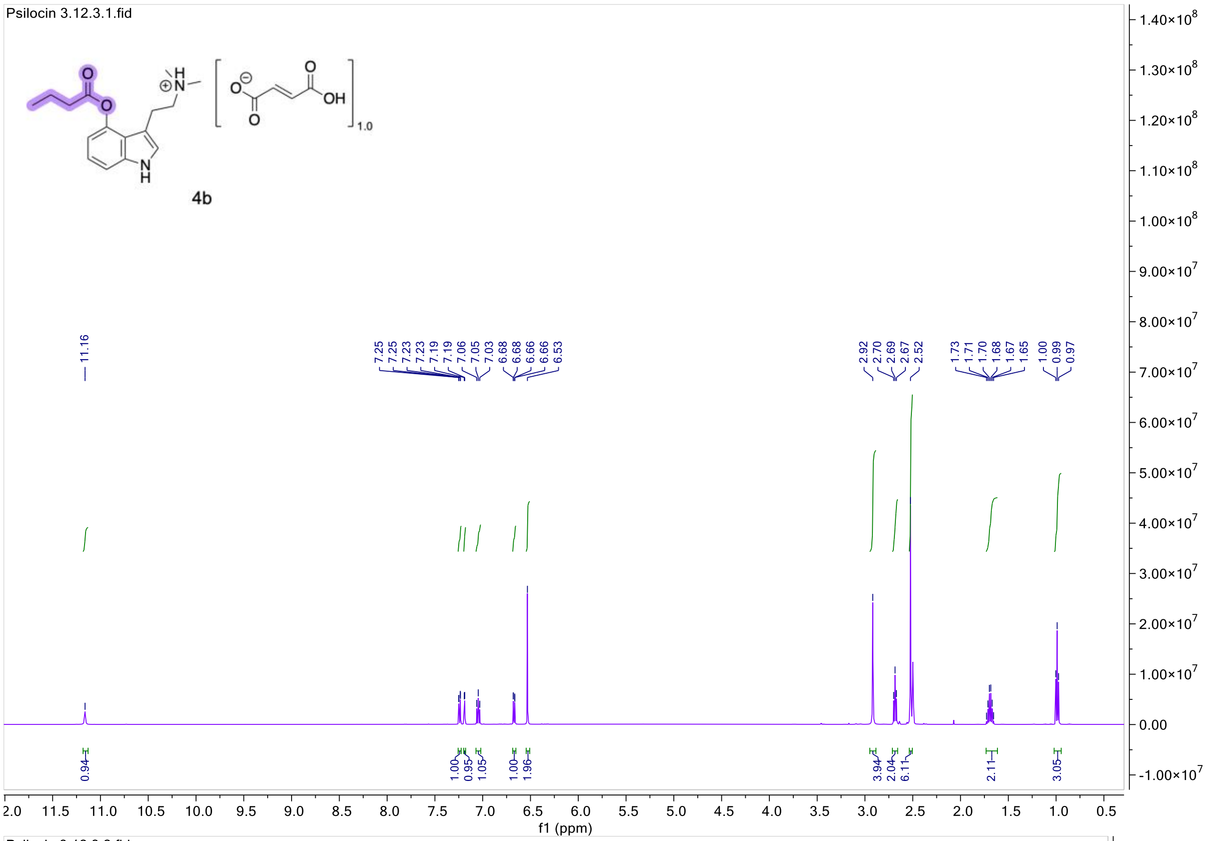
Psilocin 3.12.3.1.fid
4b
1.0
f1 (ppm)

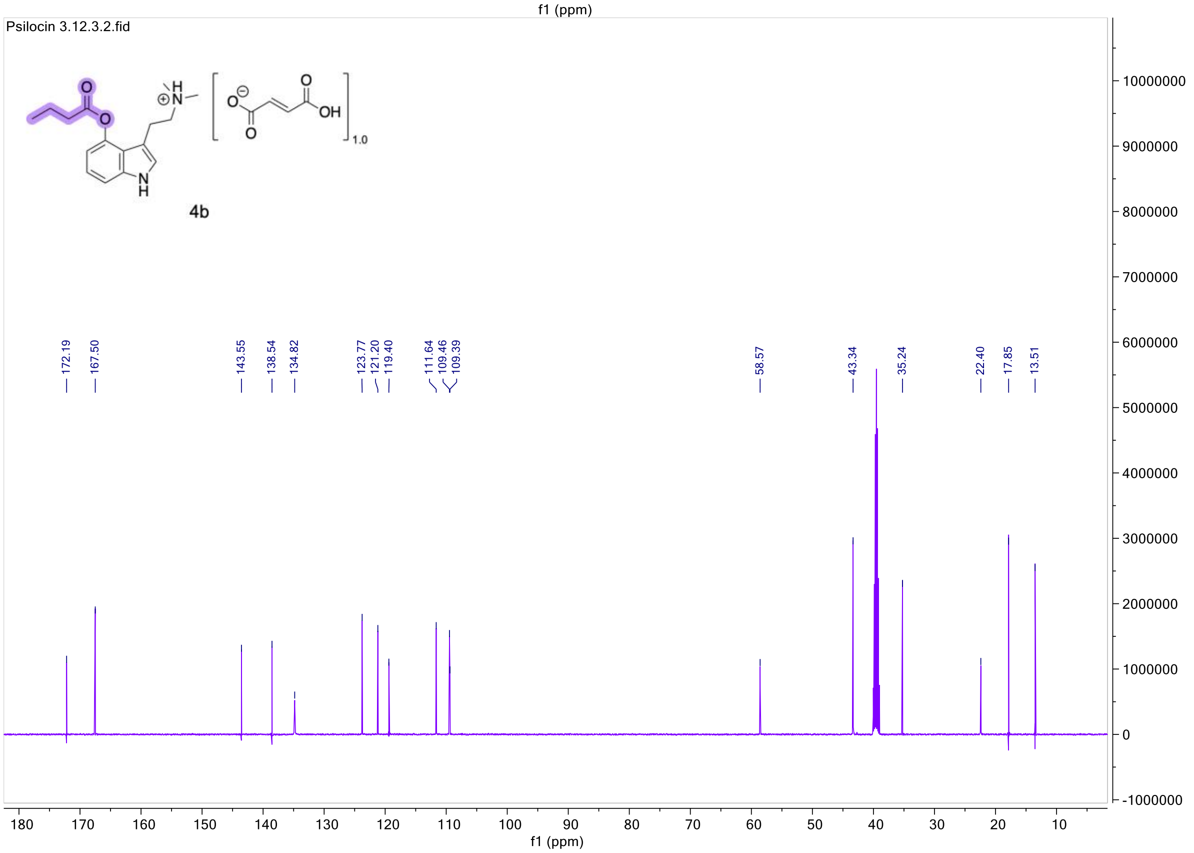
Psilocin 3.12.3.2.fid
4b
1.0
f1 (ppm)

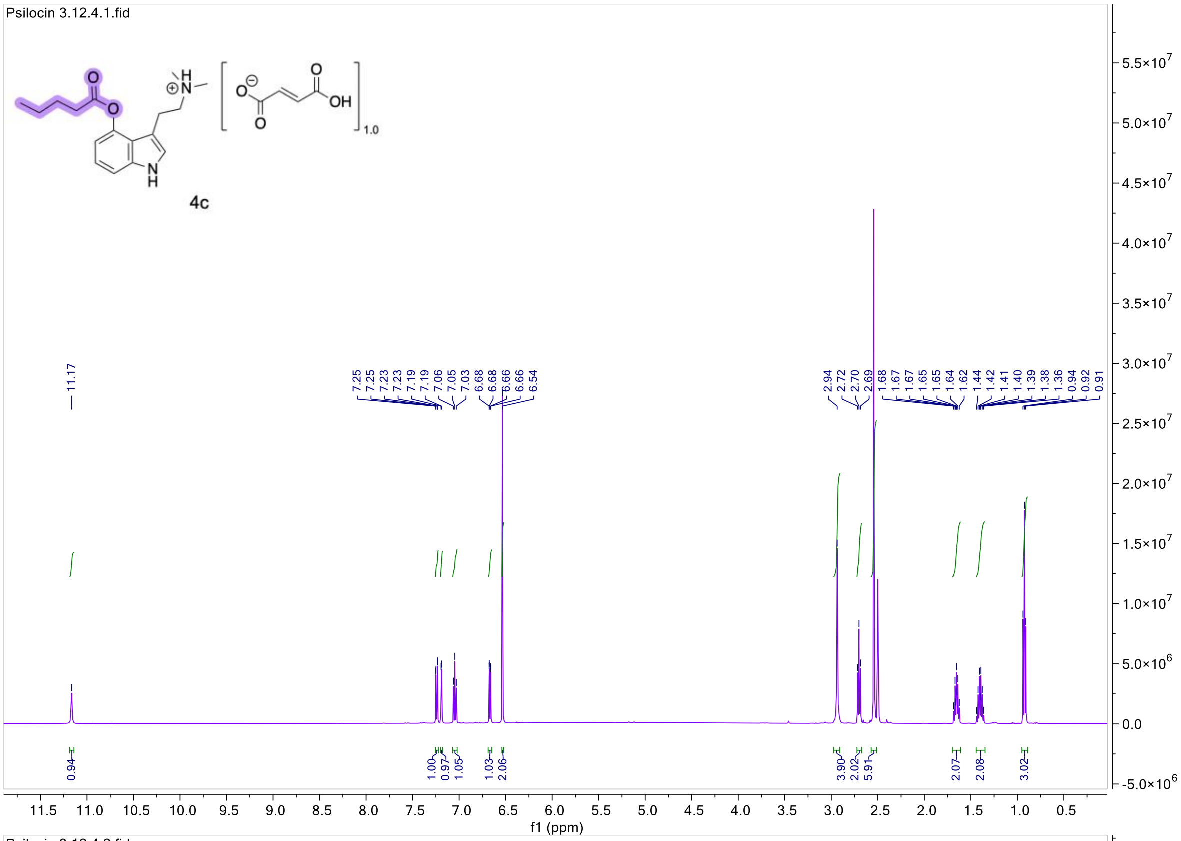
Psilocin 3.12.4.1.fid
4c
11.17
7.25 7.25 7.23 7.23 7.19 7.19 7.06 7.05 7.03 6.68 6.68 6.66 6.66 6.54
2.94 2.72 2.70 2.69 1.68 1.67 1.67 1.65 1.65 1.64 1.62 1.44 1.42 1.41 1.40 1.39 1.38 1.36 0.94 0.92 0.91
0.94 1.00 0.97 1.05 1.03 2.06 3.90 2.02 5.91 2.07 2.08 3.02
f1 (ppm)

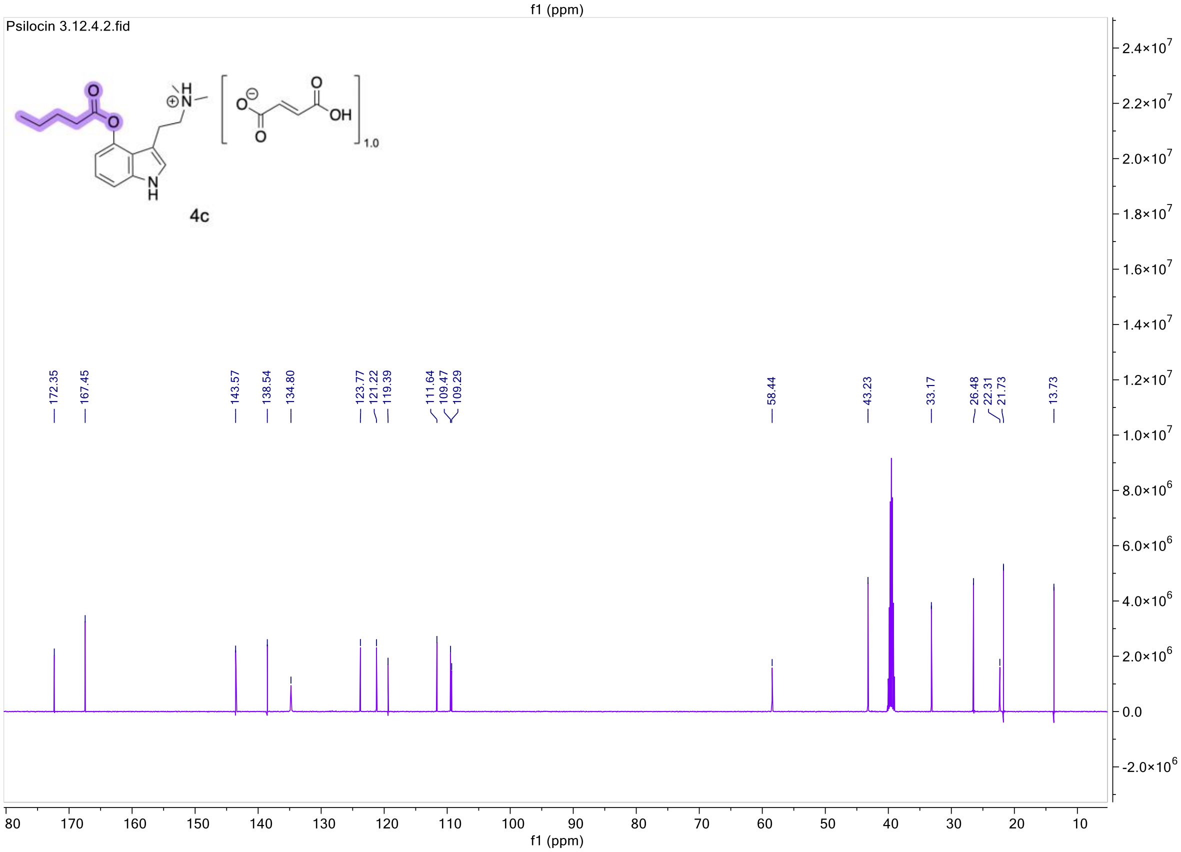
Psilocin 3.12.4.2.fid
4c
172.35 167.45 143.57 138.54 134.80 123.77 121.22 119.39 111.64 109.47 109.29 58.44 43.23 33.17 26.48 22.31 21.73 13.73
f1 (ppm)

Psilocin 3.14.2.1.fid

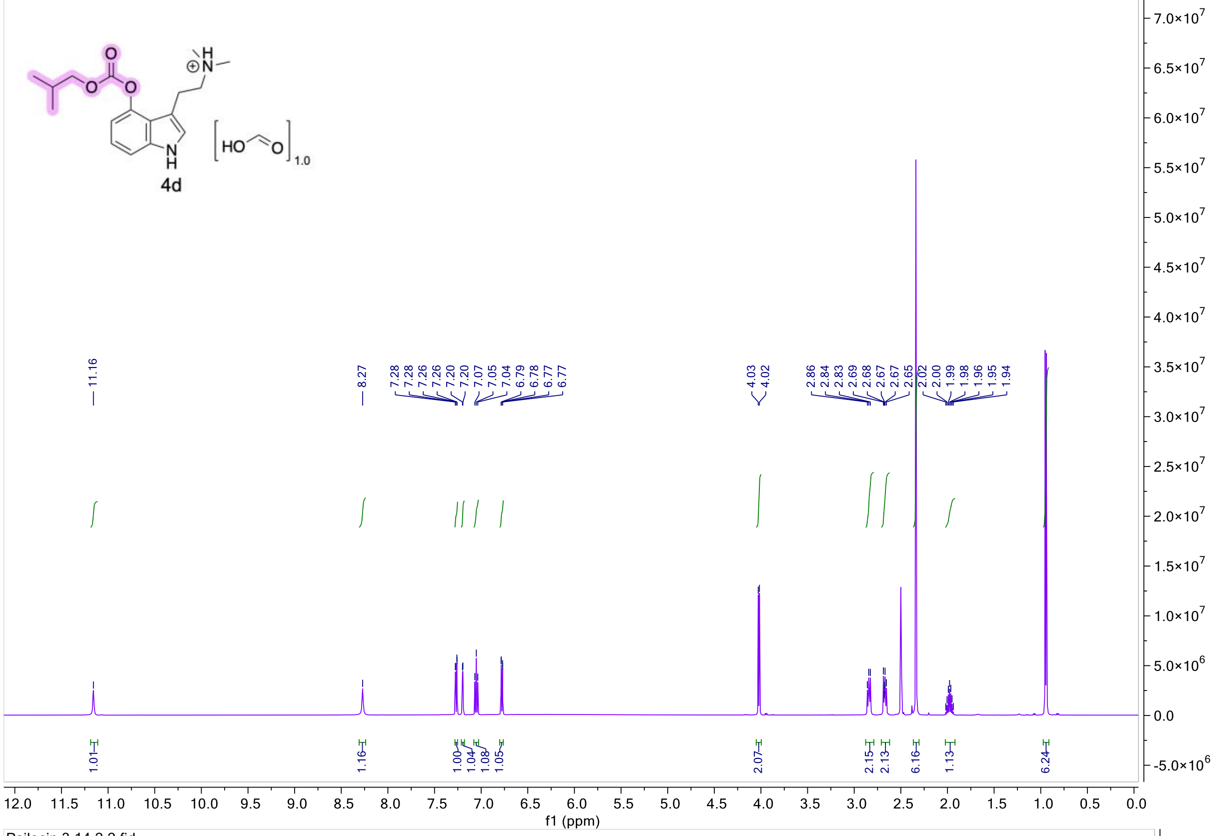

Psilocin 3.14.2.2.fid

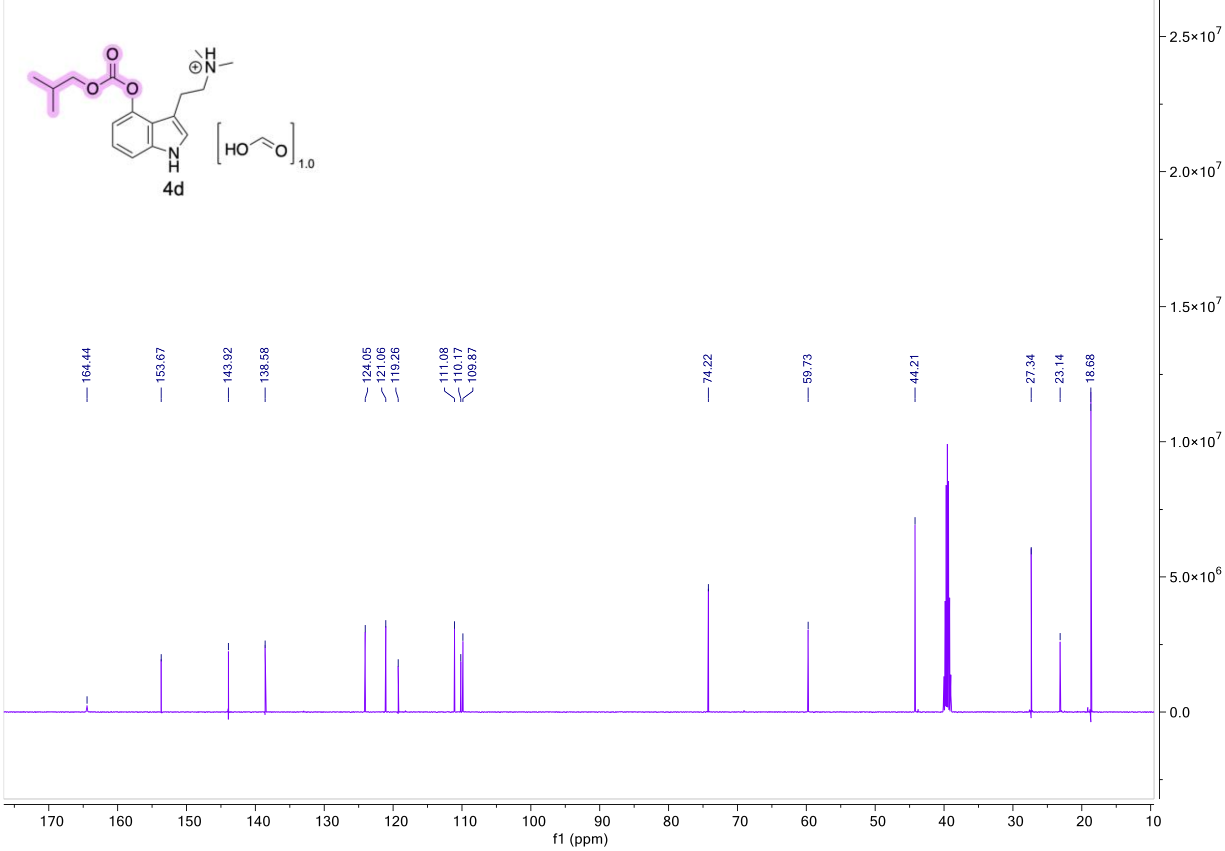

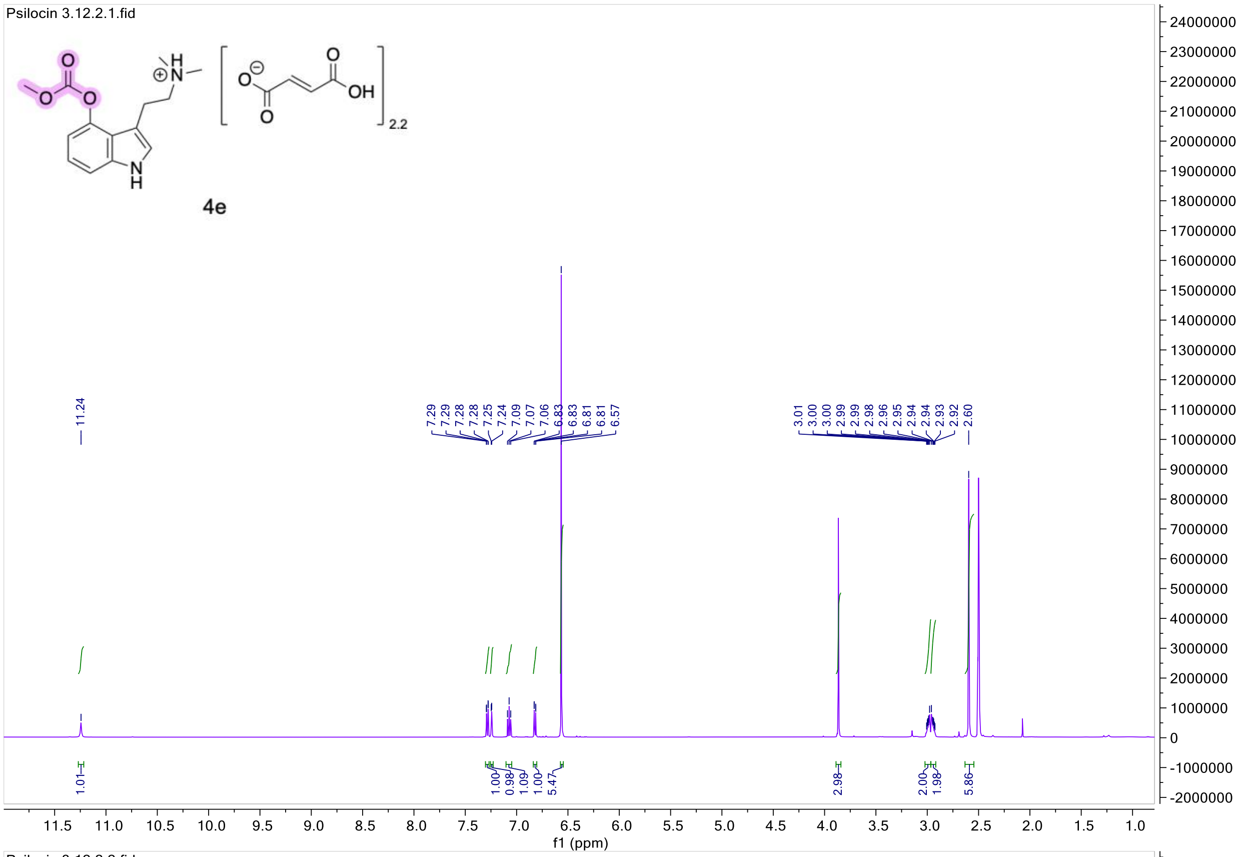
Psilocin 3.12.2.1.fid
4e
f1 (ppm)

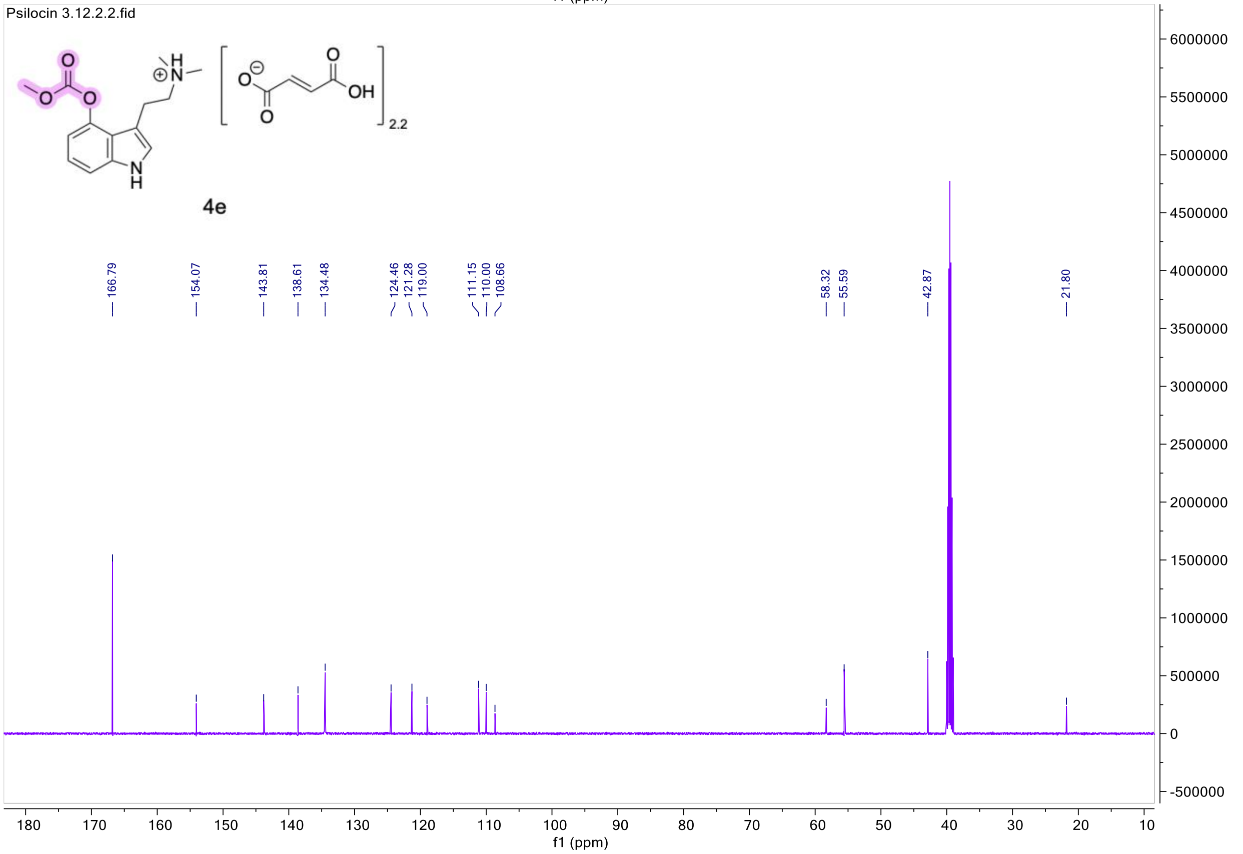
Psilocin 3.12.2.2.fid
4e
f1 (ppm)

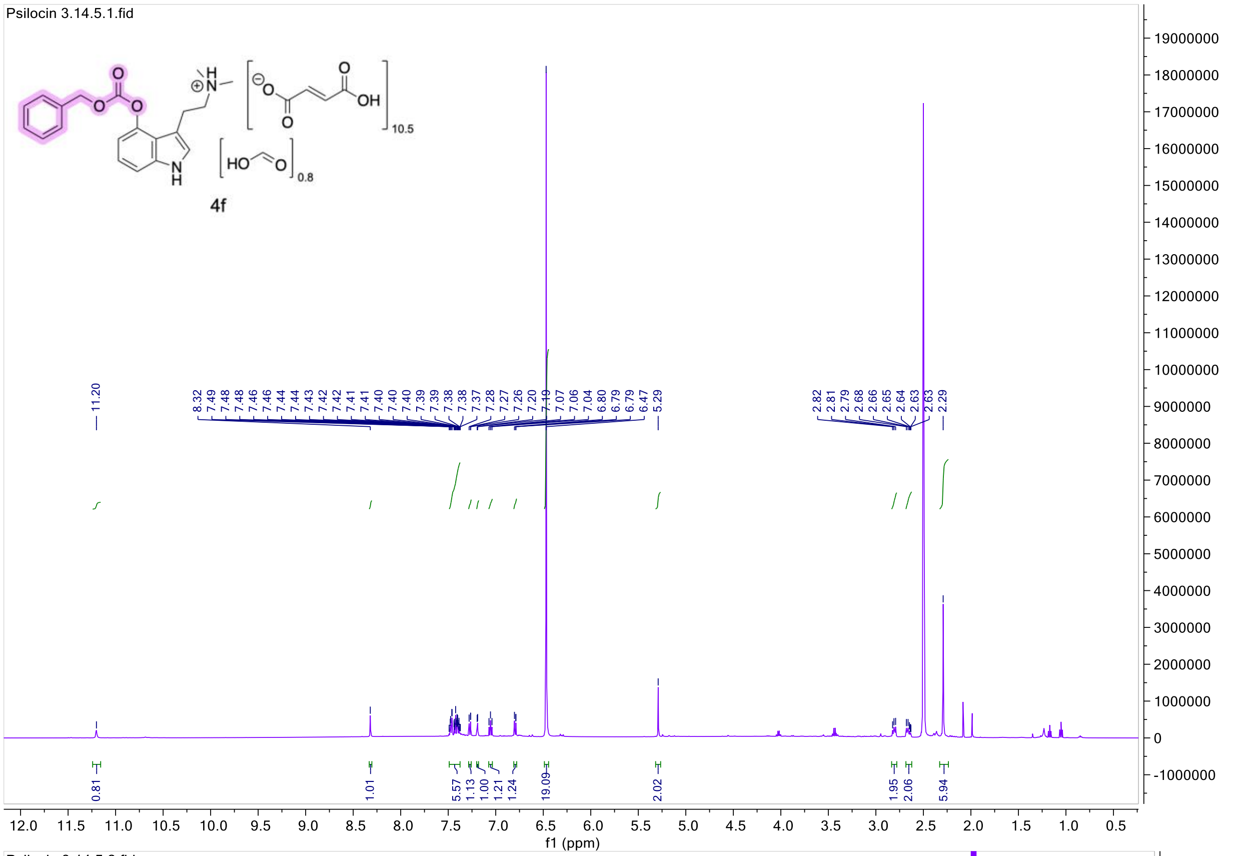

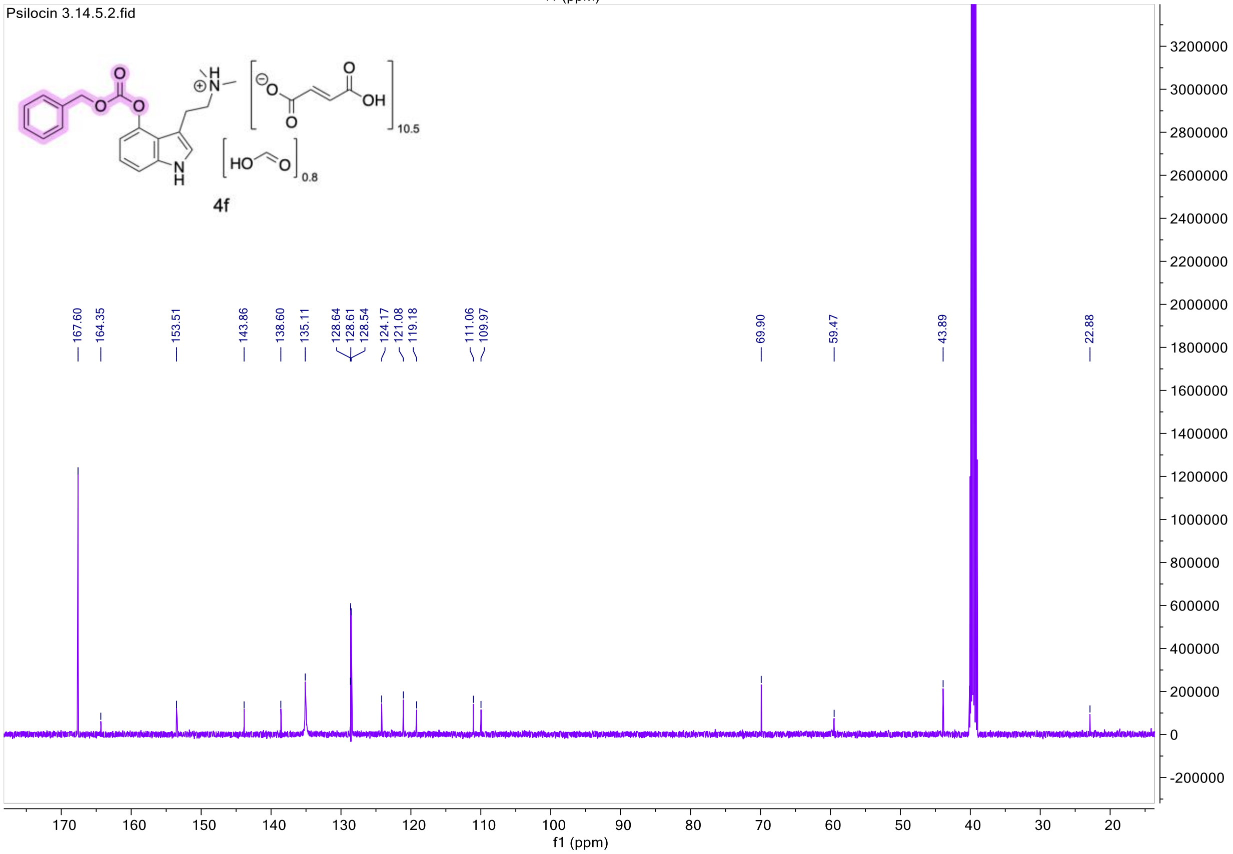

Psilocin 3.11.4-2.1.fid

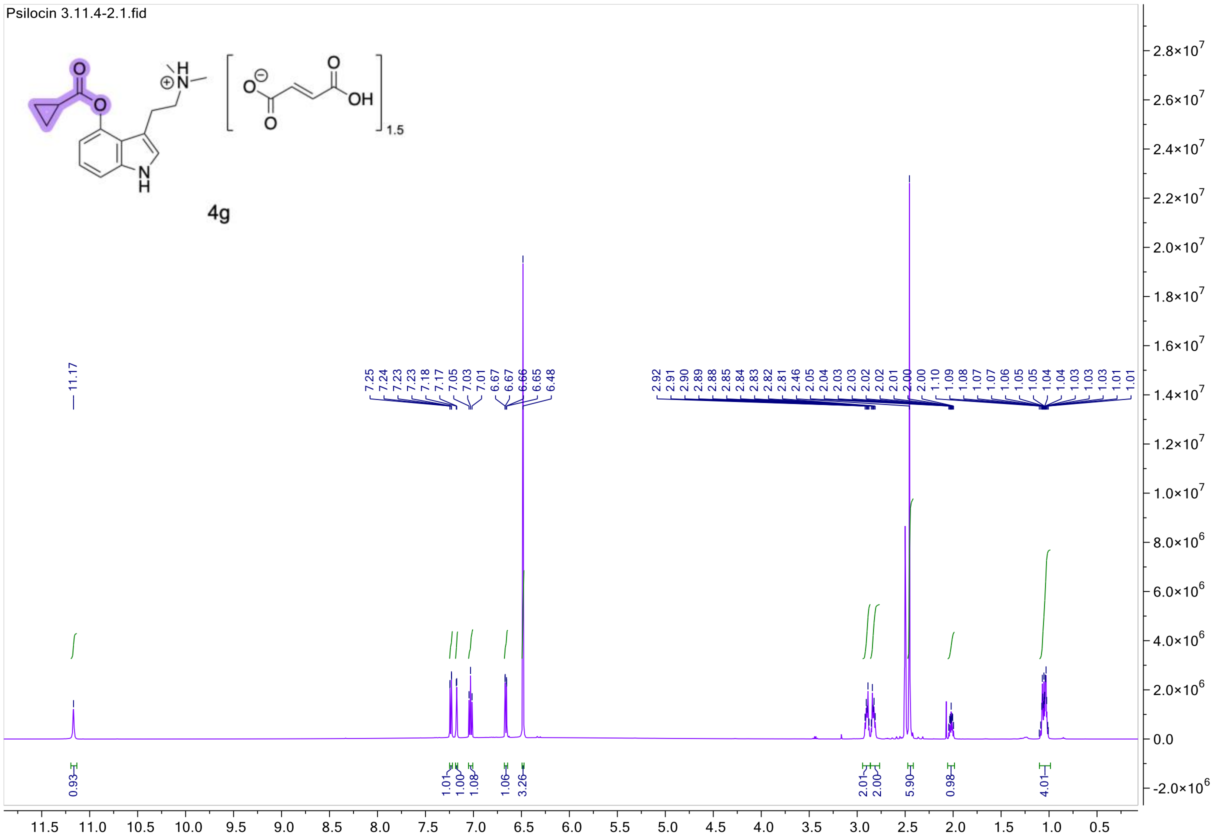

Psilocin 3.11.4-2.2.fid

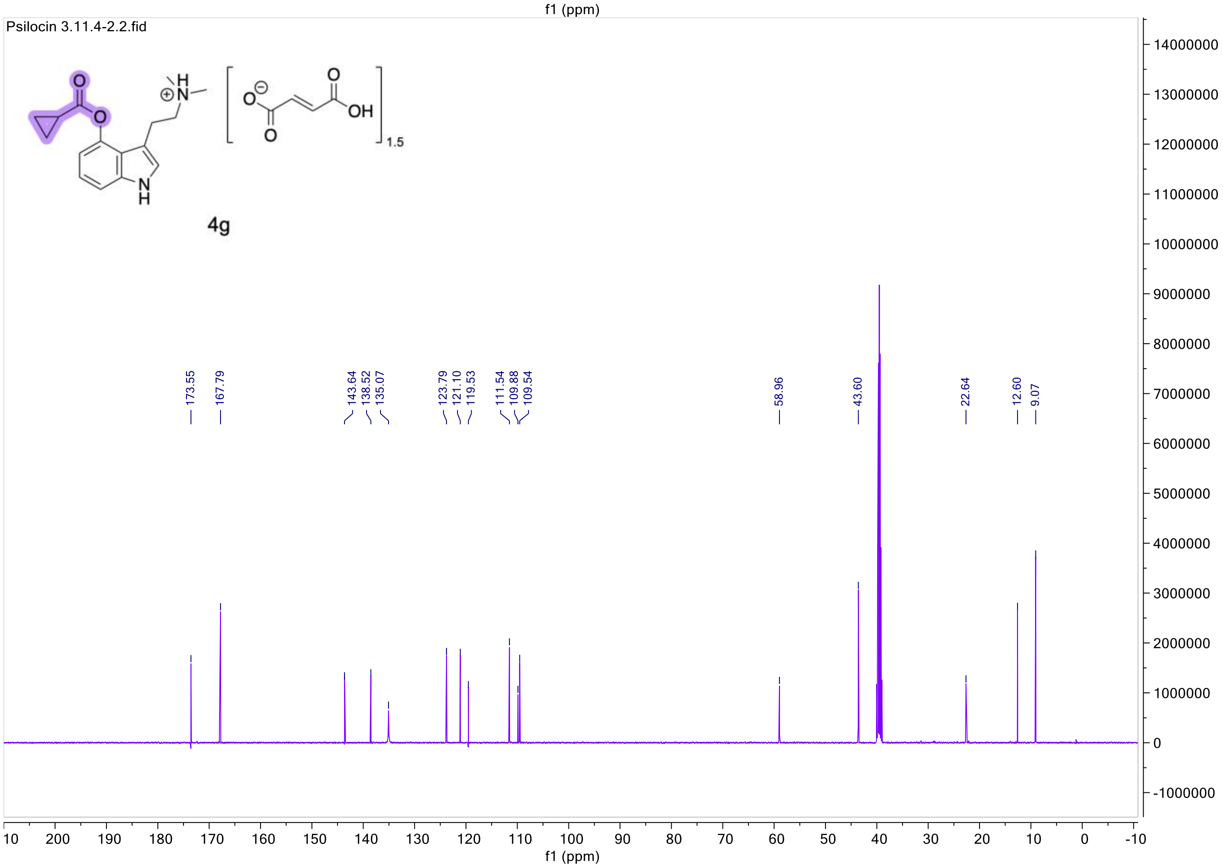

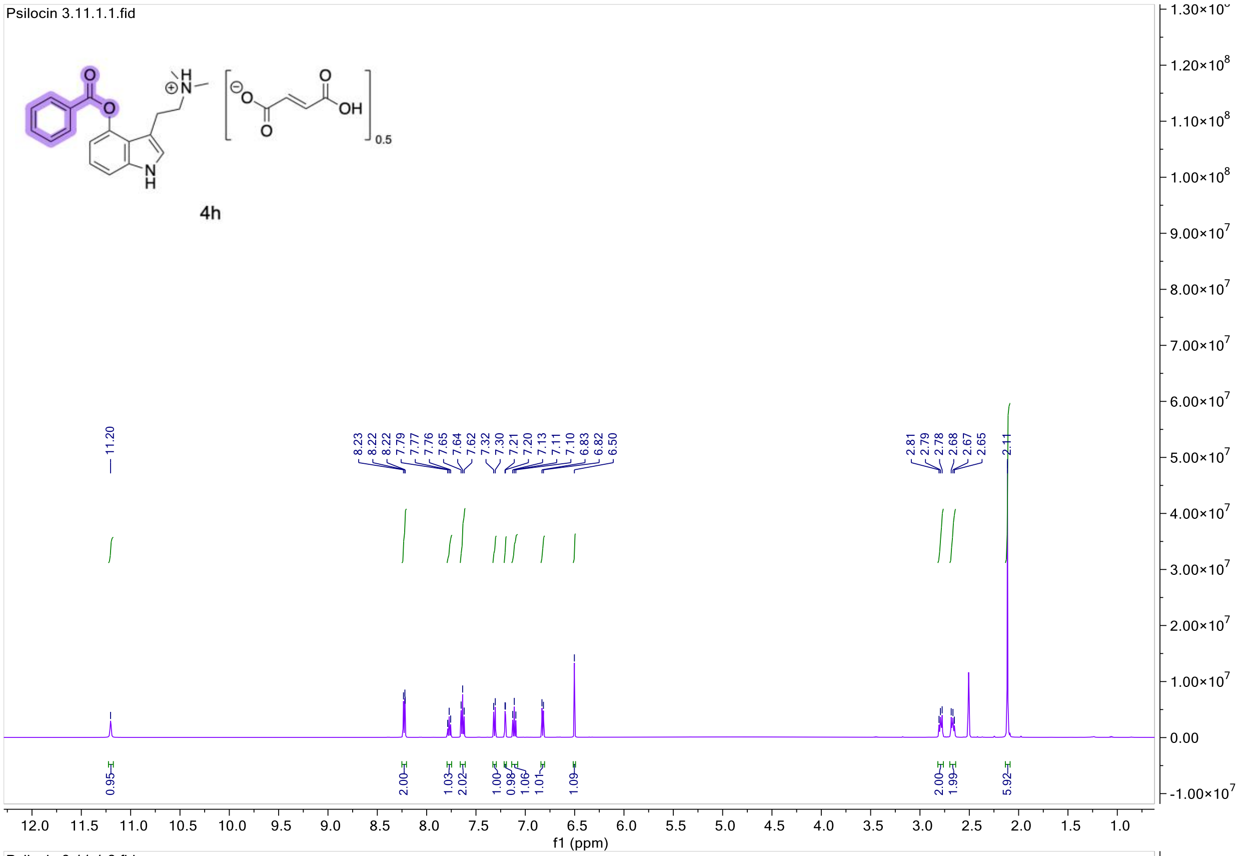
Psilocin 3.11.1.1.fid
4h
11.20
8.23
8.22
8.22
7.79
7.77
7.76
7.65
7.64
7.62
7.32
7.30
7.21
7.20
7.13
7.11
7.10
6.83
6.82
6.50
2.81
2.79
2.78
2.68
2.67
2.65
2.11
0.95
2.00
1.03
2.02
1.00
0.98
1.06
1.01
1.09
2.00
1.99
5.92
f1 (ppm)

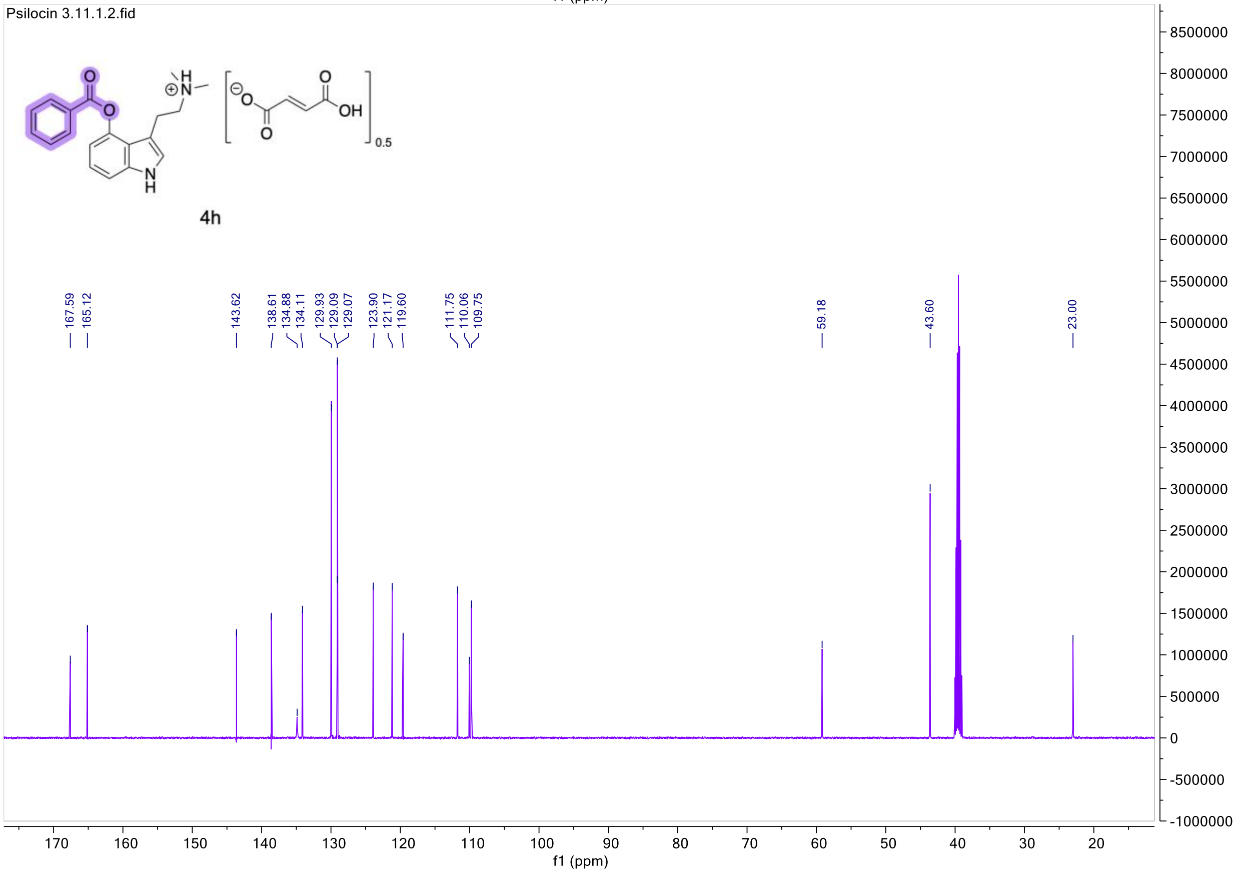
Psilocin 3.11.1.2.fid
4h
167.59
165.12
143.62
138.61
134.88
134.11
129.93
129.09
129.07
123.90
121.17
119.60
111.75
110.06
109.75
59.18
43.60
23.00
f1 (ppm)

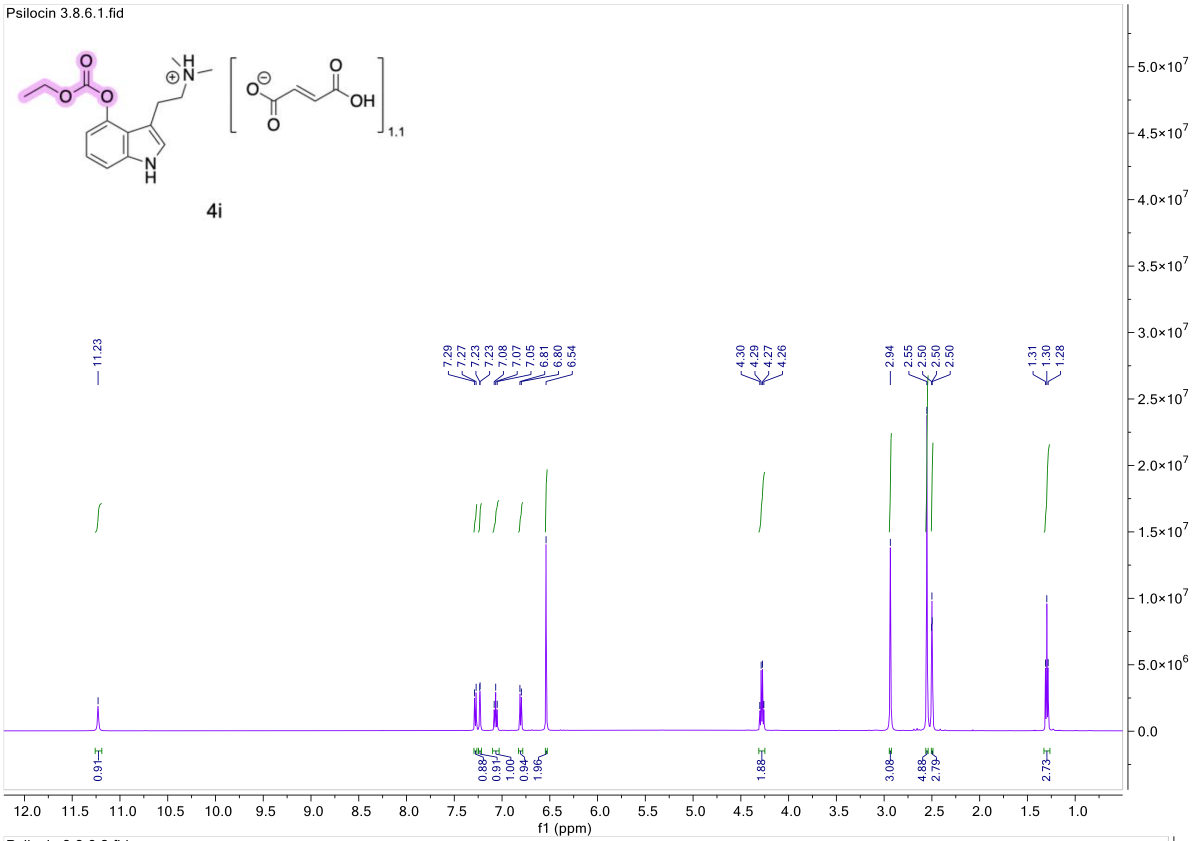

Psilocin 3.8.6.1.fid
4i
11.23
7.29 7.27 7.23 7.23 7.08 7.07 7.05 6.81 6.80 6.54
4.30 4.29 4.27 4.26
2.94 2.55 2.50 2.50 2.50 2.50
1.31 1.30 1.28
0.91 0.88 0.91 1.00 0.94 1.96 1.88 3.08 4.88 2.79 2.73
f1 (ppm)

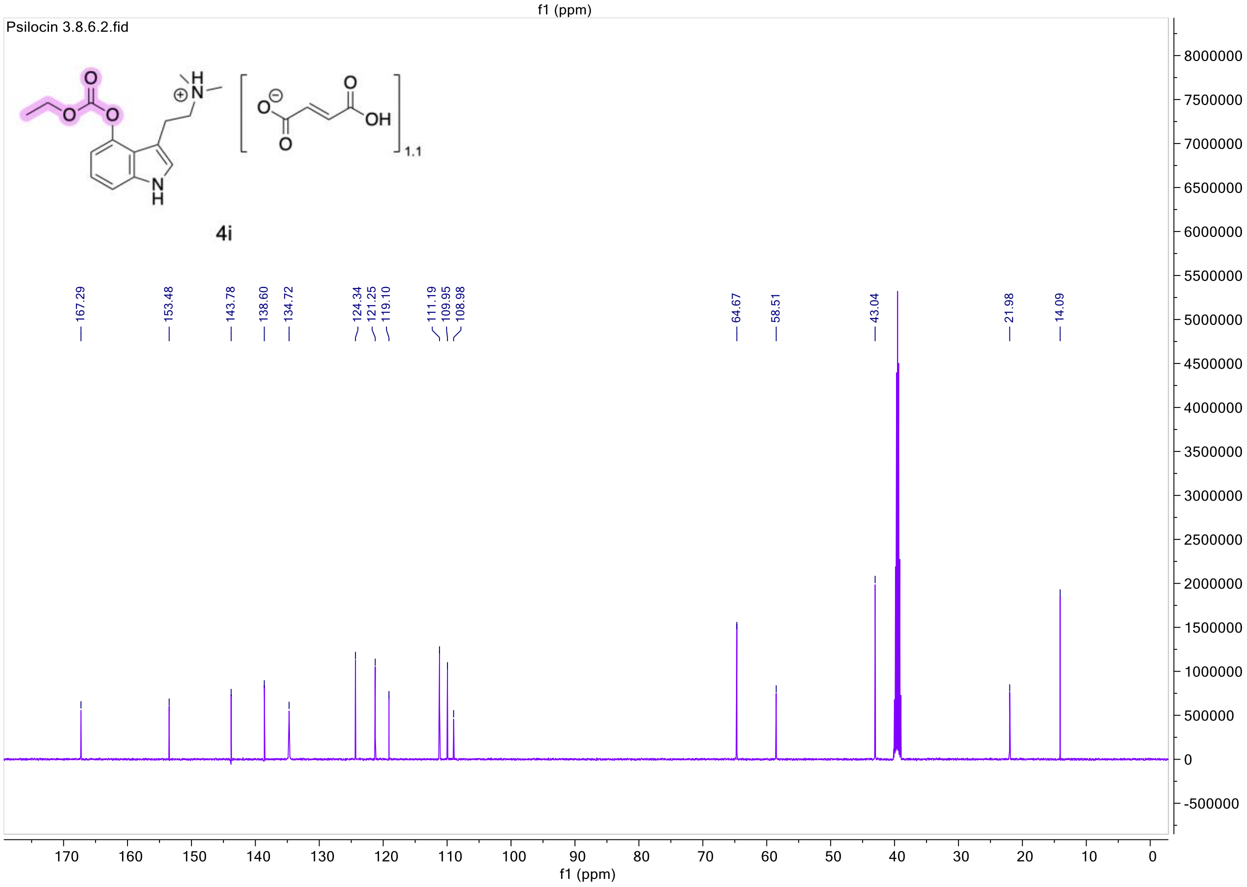

Psilocin 3.8.6.2.fid
4i
167.29 153.48 143.78 138.60 134.72 124.34 121.25 119.10 111.19 109.95 108.98 64.67 58.51 43.04 21.98 14.09
f1 (ppm)

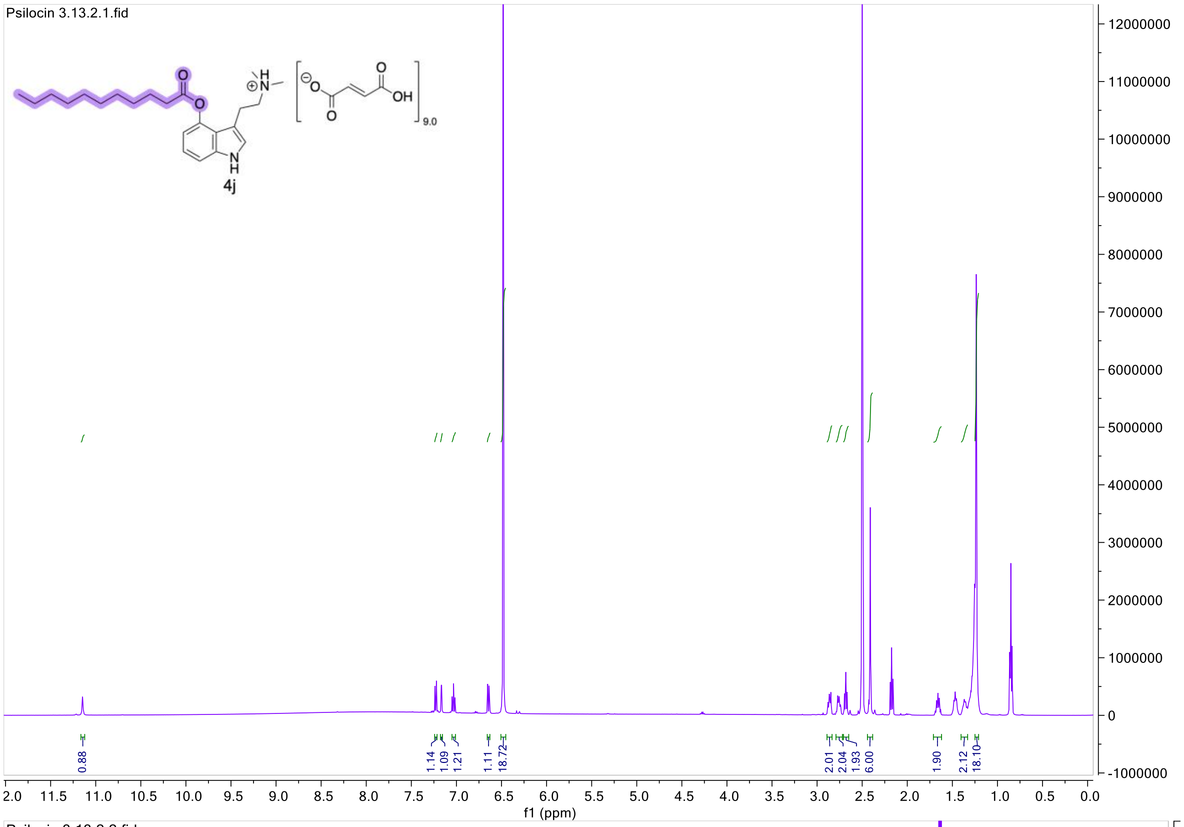

Psilocin 3.13.2.1.fid
4j
9.0
0.88
1.14
1.09
1.21
1.11
18.72
2.01
2.04
1.93
6.00
1.90
2.12
18.10
f1 (ppm)

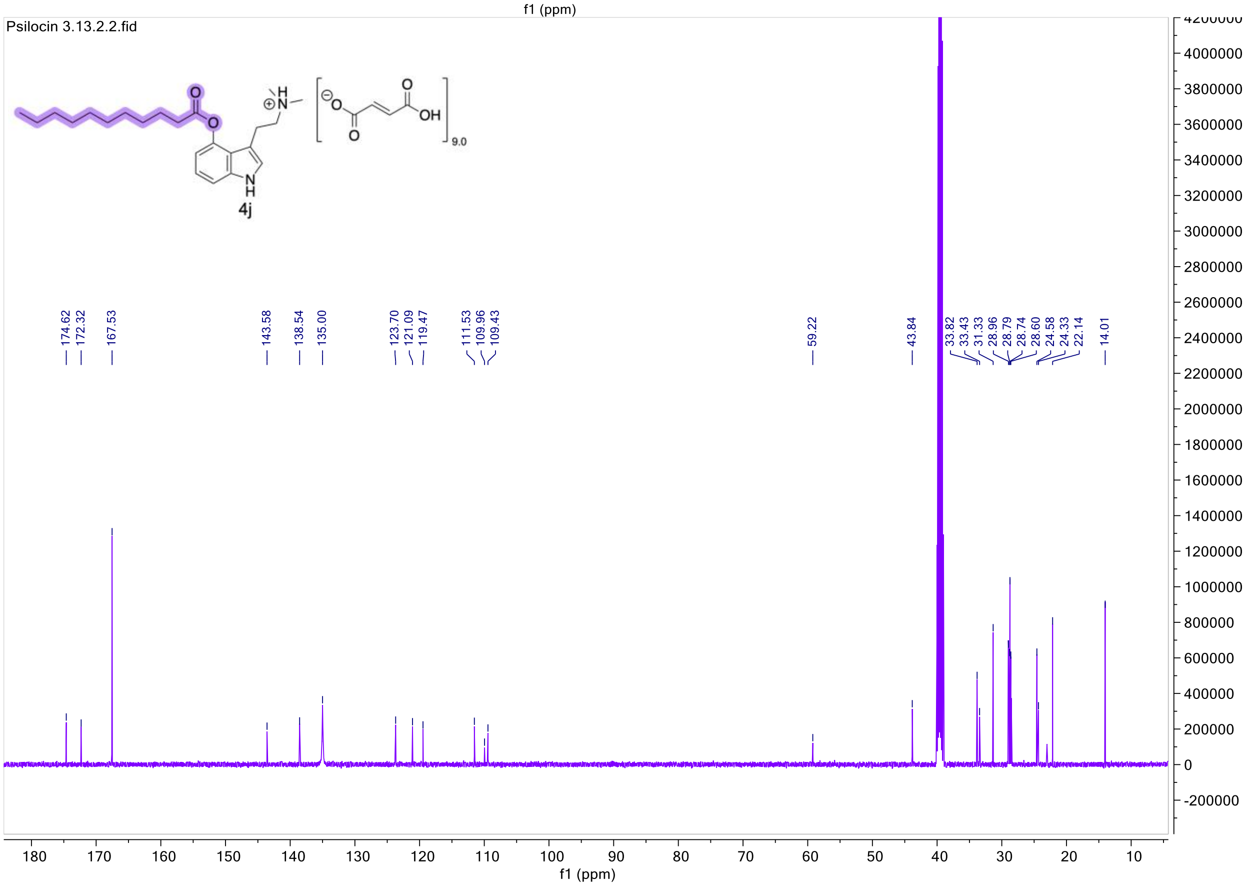

Psilocin 3.13.2.2.fid
4j
9.0
174.62
172.32
167.53
143.58
138.54
135.00
123.70
121.09
119.47
111.53
109.96
109.43
59.22
43.84
33.82
33.43
31.33
28.96
28.79
28.74
28.60
24.58
24.33
22.14
14.01
f1 (ppm)

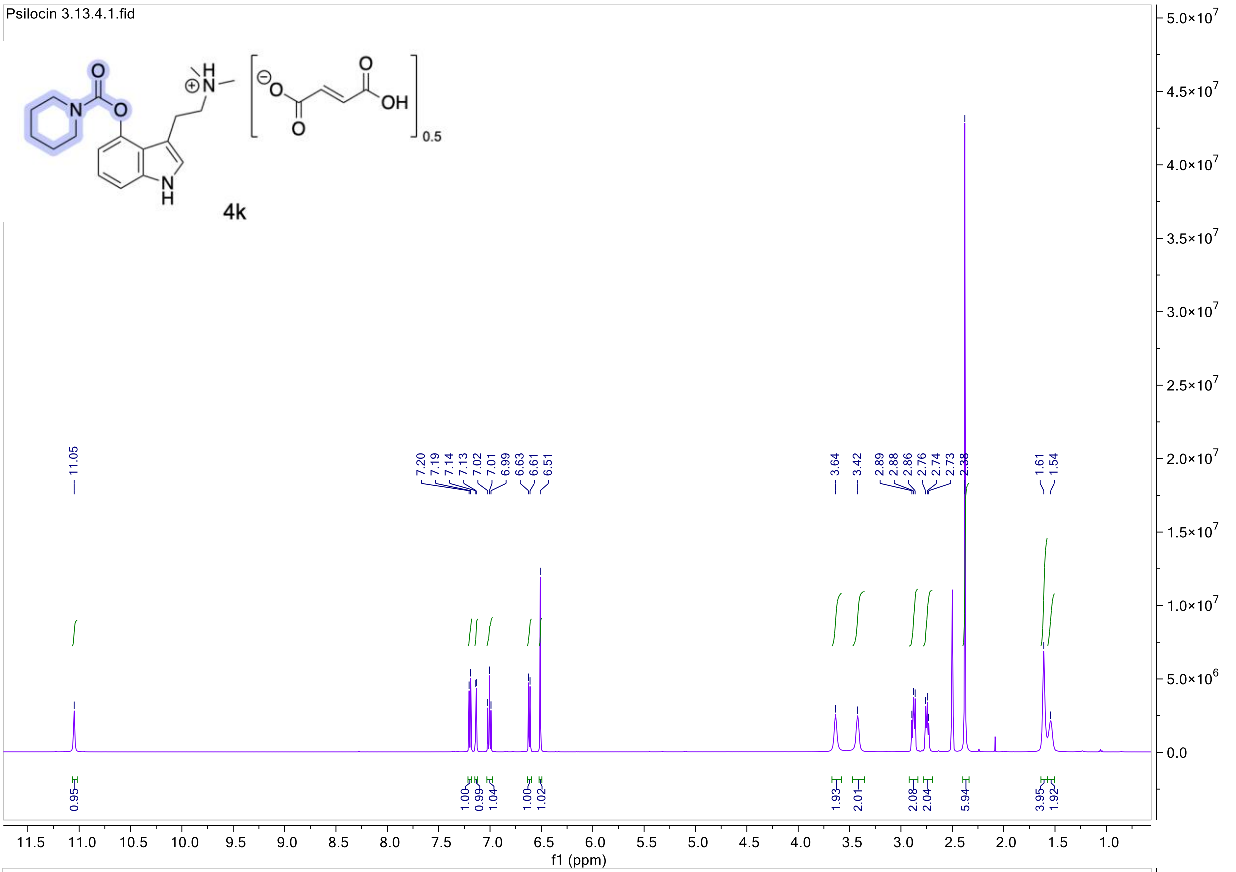
Psilocin 3.13.4.1.fid
4k
0.5
11.05
7.20 7.19 7.14 7.13 7.02 7.01 6.99 6.63 6.61 6.51
3.64 3.42 2.89 2.88 2.86 2.76 2.74 2.73 2.38
1.61 1.54
0.95 1.00 0.99 1.04 1.00 1.02 1.93 2.01 2.08 2.04 5.94 3.95 1.92
f1 (ppm)

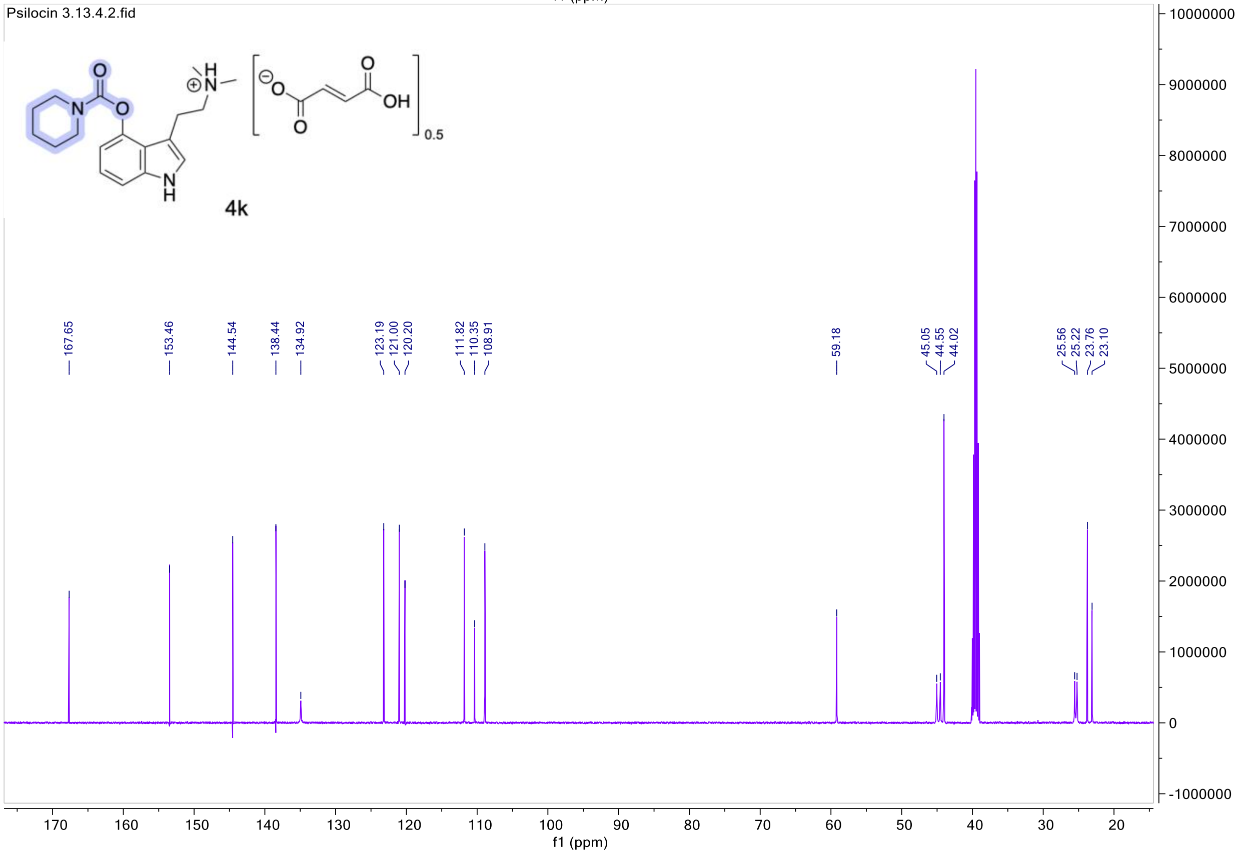
Psilocin 3.13.4.2.fid
4k
0.5
167.65 153.46 144.54 138.44 134.92 123.19 121.00 120.20 111.82 110.35 108.91
59.18 45.05 44.55 44.02 25.56 25.22 23.76 23.10
f1 (ppm)

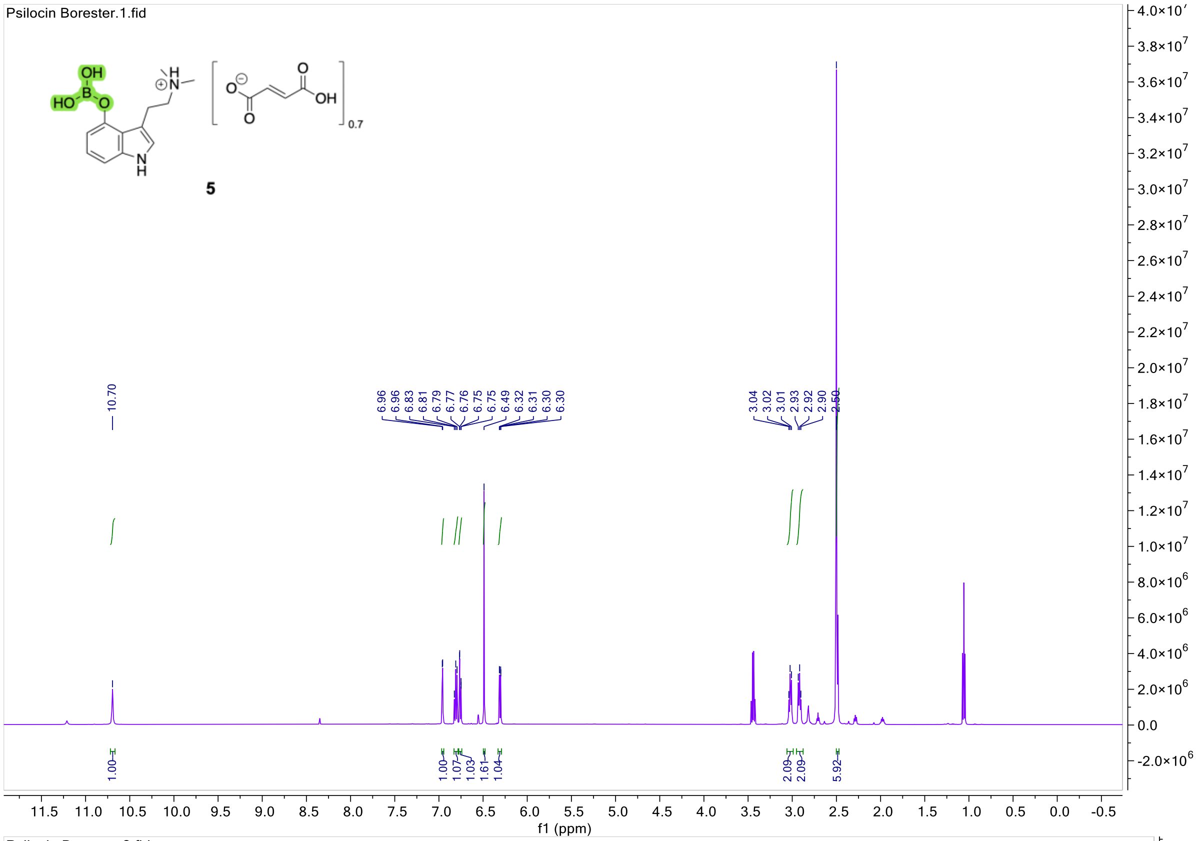
Psilocin Borester.1.fid
5
10.70
6.96
6.96
6.83
6.81
6.79
6.77
6.76
6.75
6.75
6.49
6.32
6.31
6.30
6.30
3.04
3.02
3.01
2.93
2.92
2.90
2.50
1.00
1.07
1.03
1.61
1.04
2.09
2.09
5.92
f1 (ppm)

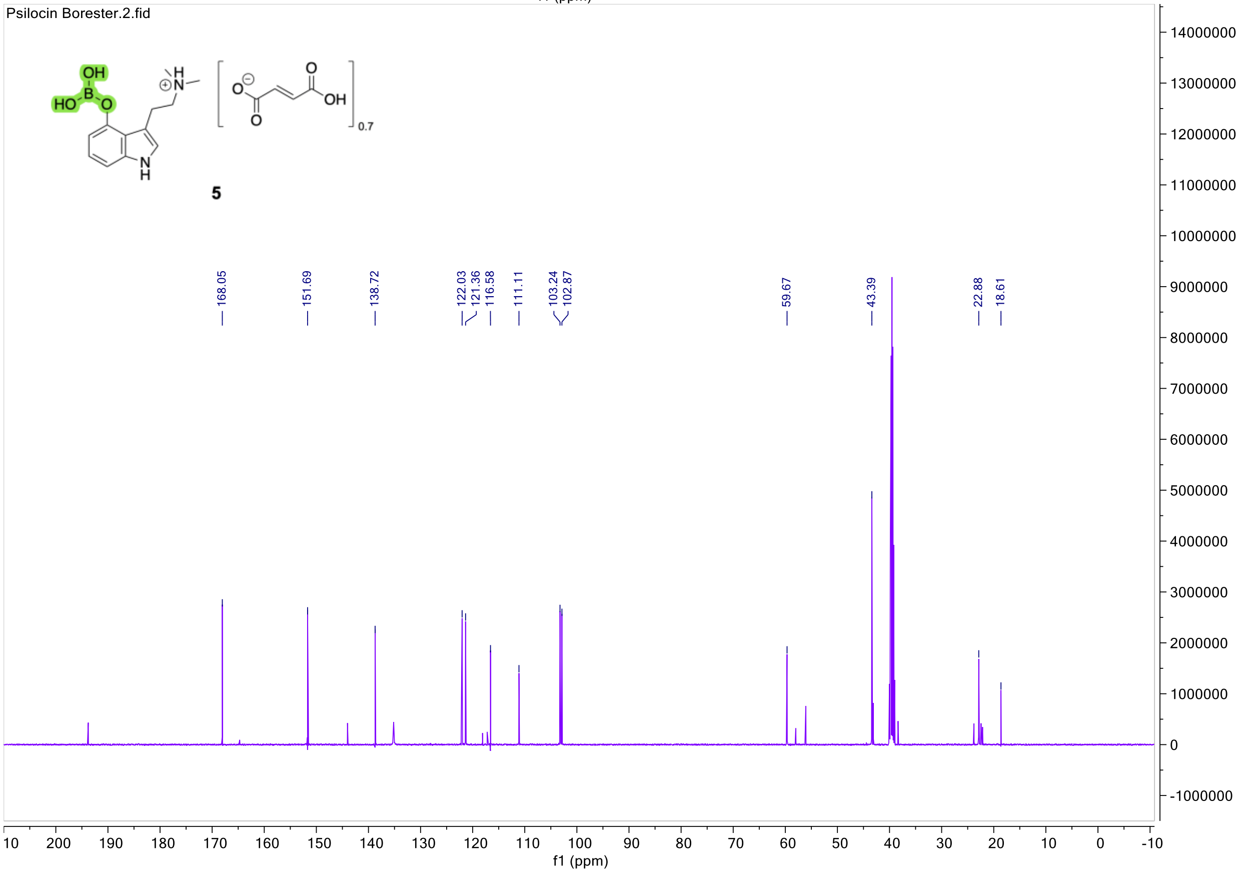
Psilocin Borester.2.fid
5
168.05
151.69
138.72
122.03
121.36
116.58
111.11
103.24
102.87
59.67
43.39
22.88
18.61
f1 (ppm)

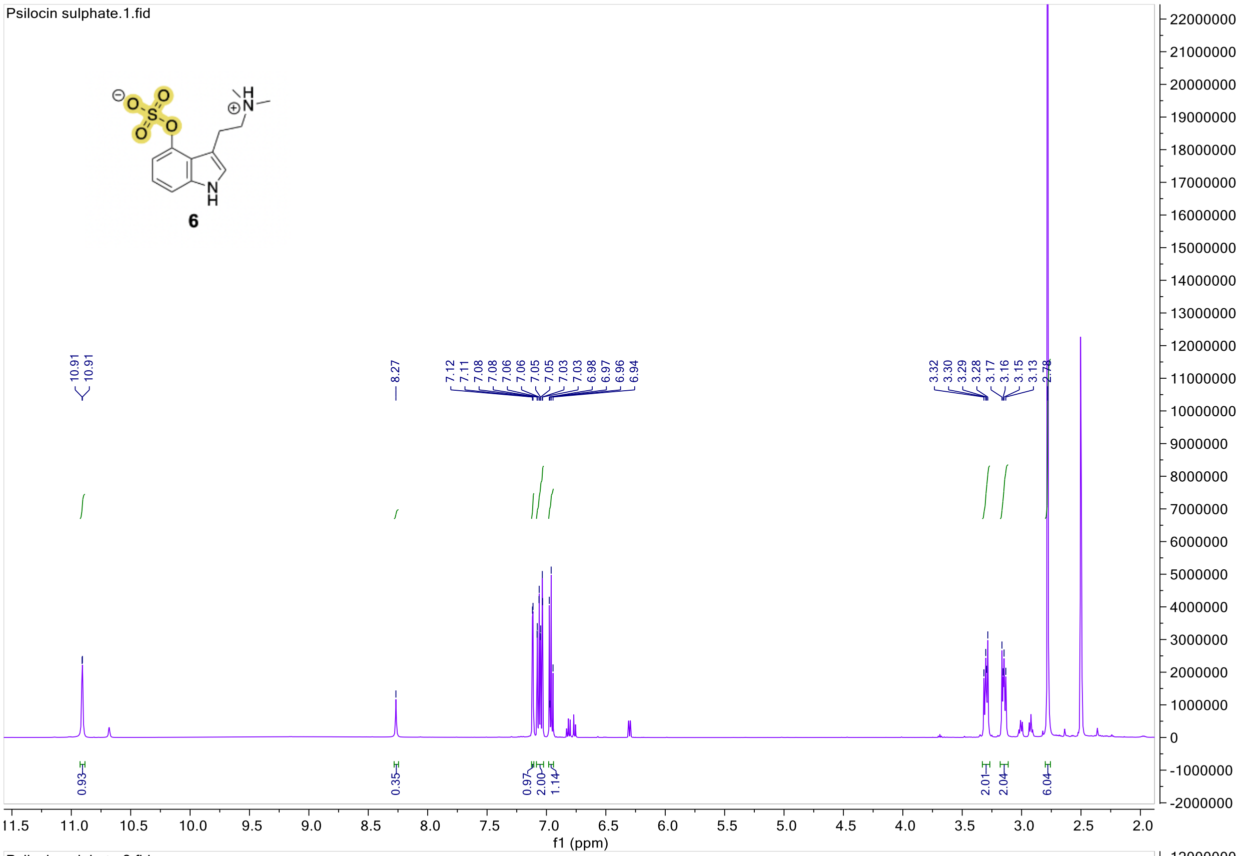

Psilocin sulphate.1.fid
6
10.91
10.91
8.27
7.12
7.11
7.08
7.08
7.06
7.06
7.05
7.05
7.03
7.03
6.98
6.97
6.96
6.94
3.32
3.30
3.29
3.28
3.17
3.16
3.15
3.13
2.78
0.93
0.35
0.97
2.00
1.14
2.01
2.04
6.04
f1 (ppm)

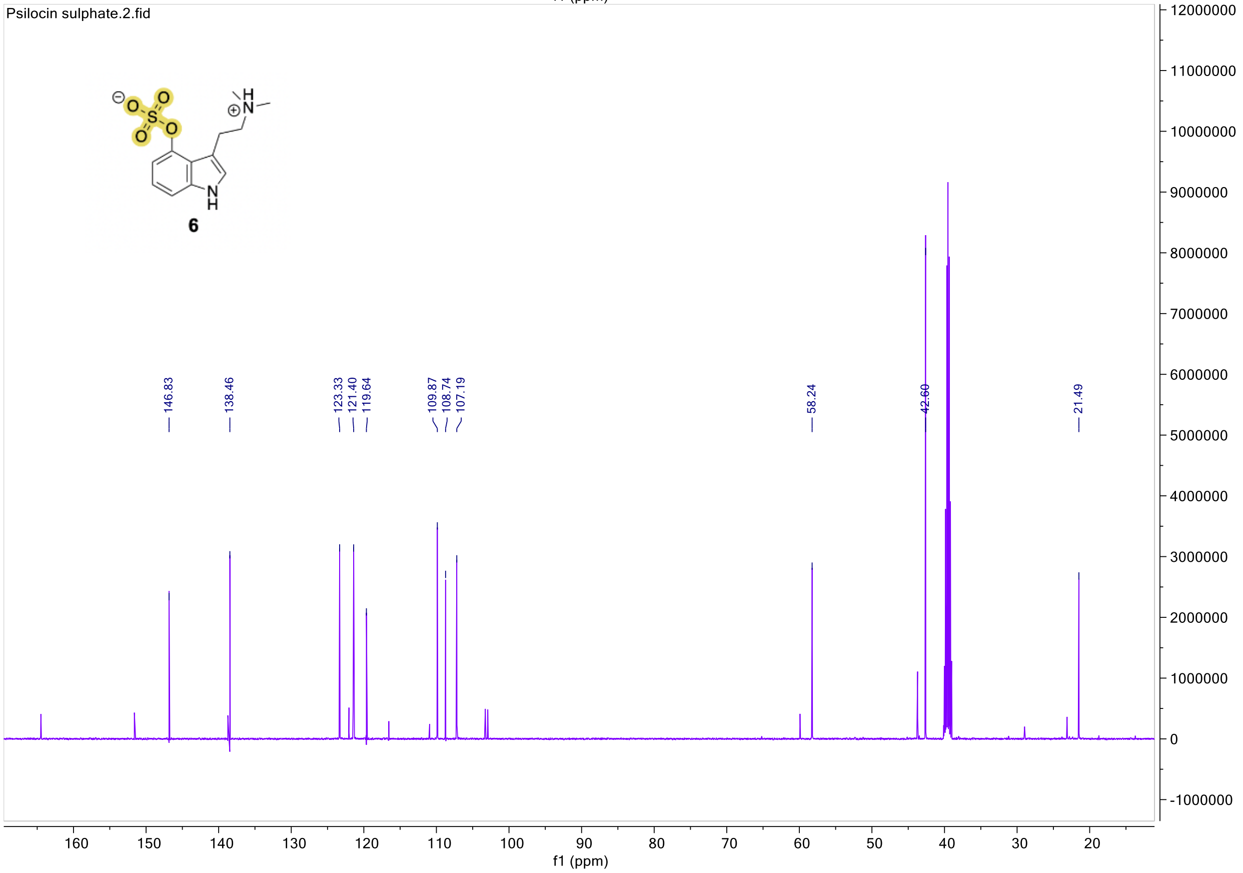

Psilocin sulphate.2.fid
6
146.83
138.46
123.33
121.40
119.64
109.87
108.74
107.19
58.24
42.60
21.49
f1 (ppm)

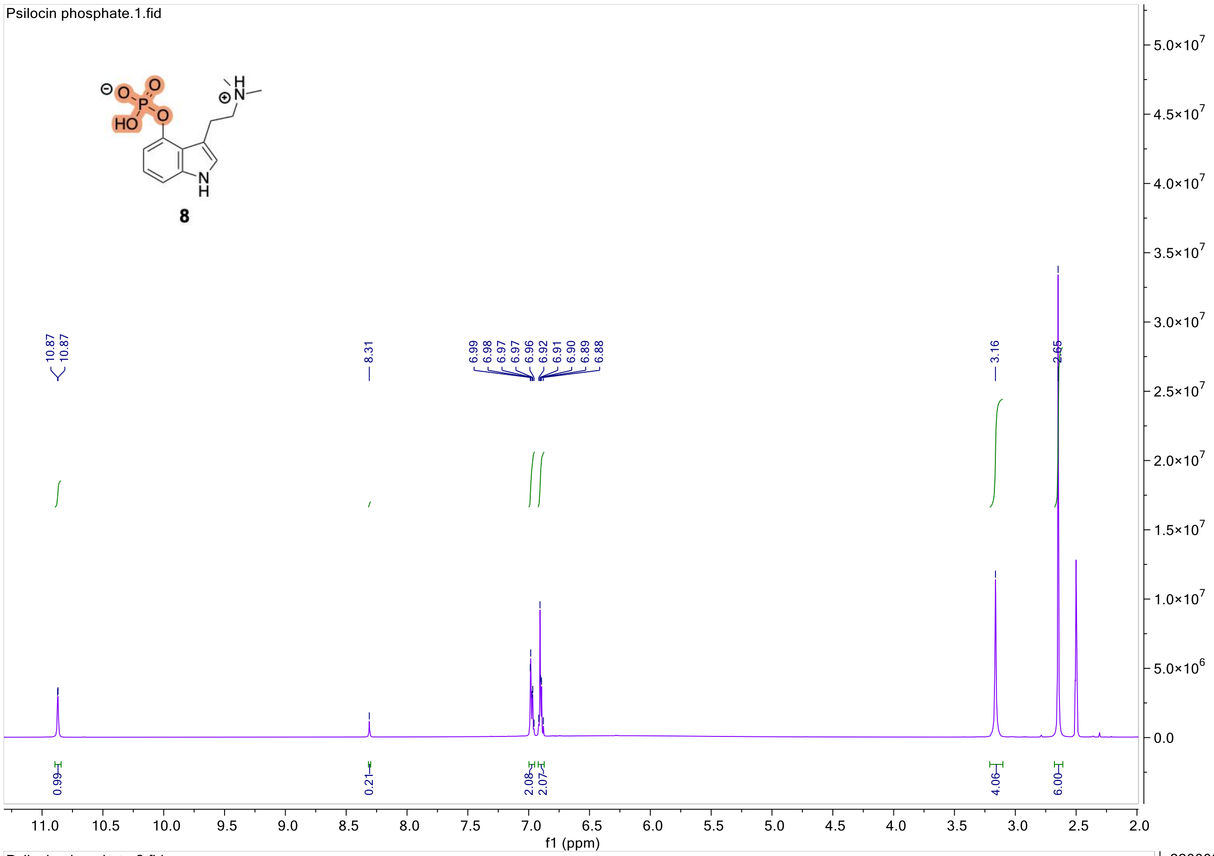
Psilocin phosphate.1.fid
HO
O
P
O
O
H
N
N
H
8
10.87
10.87
8.31
6.99
6.98
6.97
6.97
6.96
6.92
6.91
6.90
6.89
6.88
3.16
2.65
0.99
0.21
2.08
2.07
4.06
6.00
f1 (ppm)

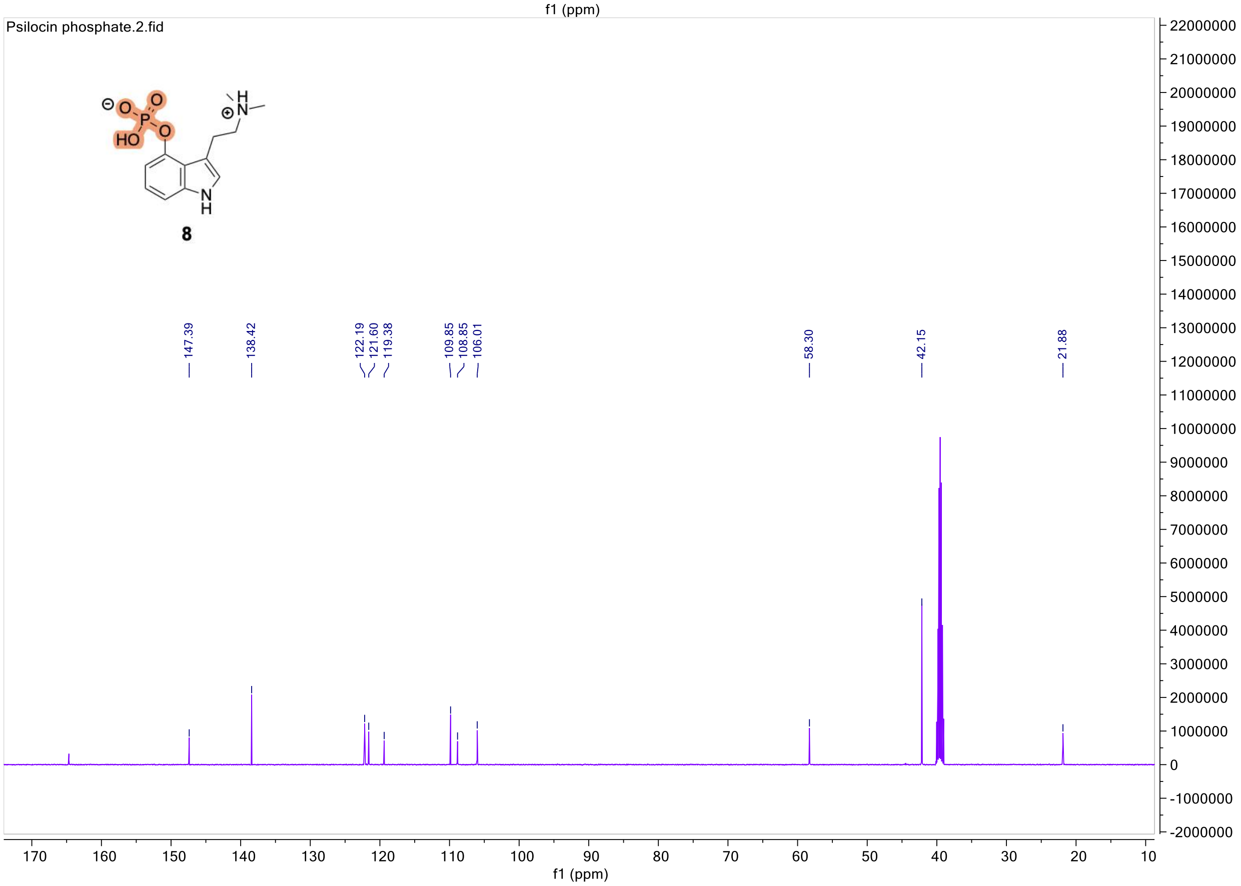
Psilocin phosphate.2.fid
HO
O
P
O
O
H
N
N
H
8
147.39
138.42
122.19
121.60
119.38
109.85
108.85
106.01
58.30
42.15
21.88
f1 (ppm)

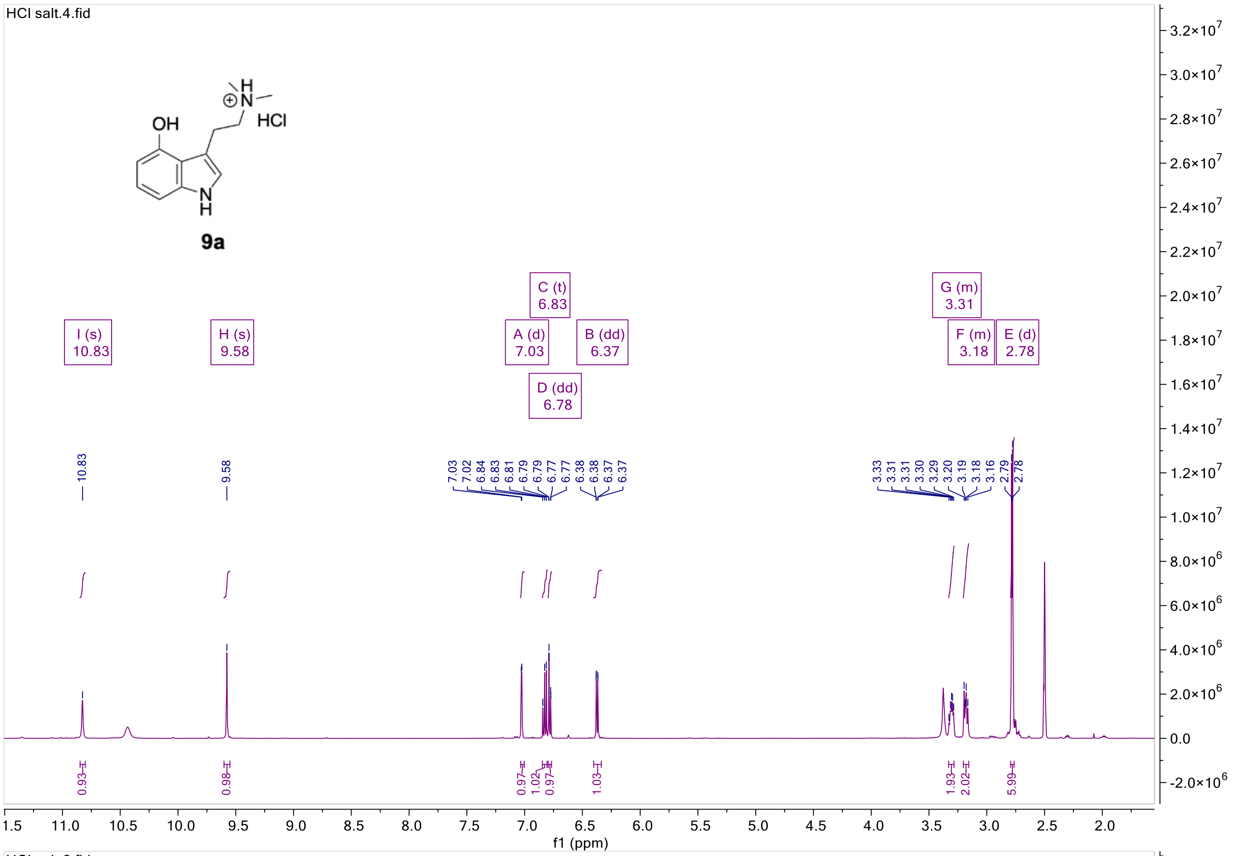
HCl salt.4.fid
OH
H
N
HCl
N
H
9a
I (s) 10.83
H (s) 9.58
A (d) 7.03
C (t) 6.83
D (dd) 6.78
B (dd) 6.37
G (m) 3.31
F (m) 3.18
E (d) 2.78
f1 (ppm)

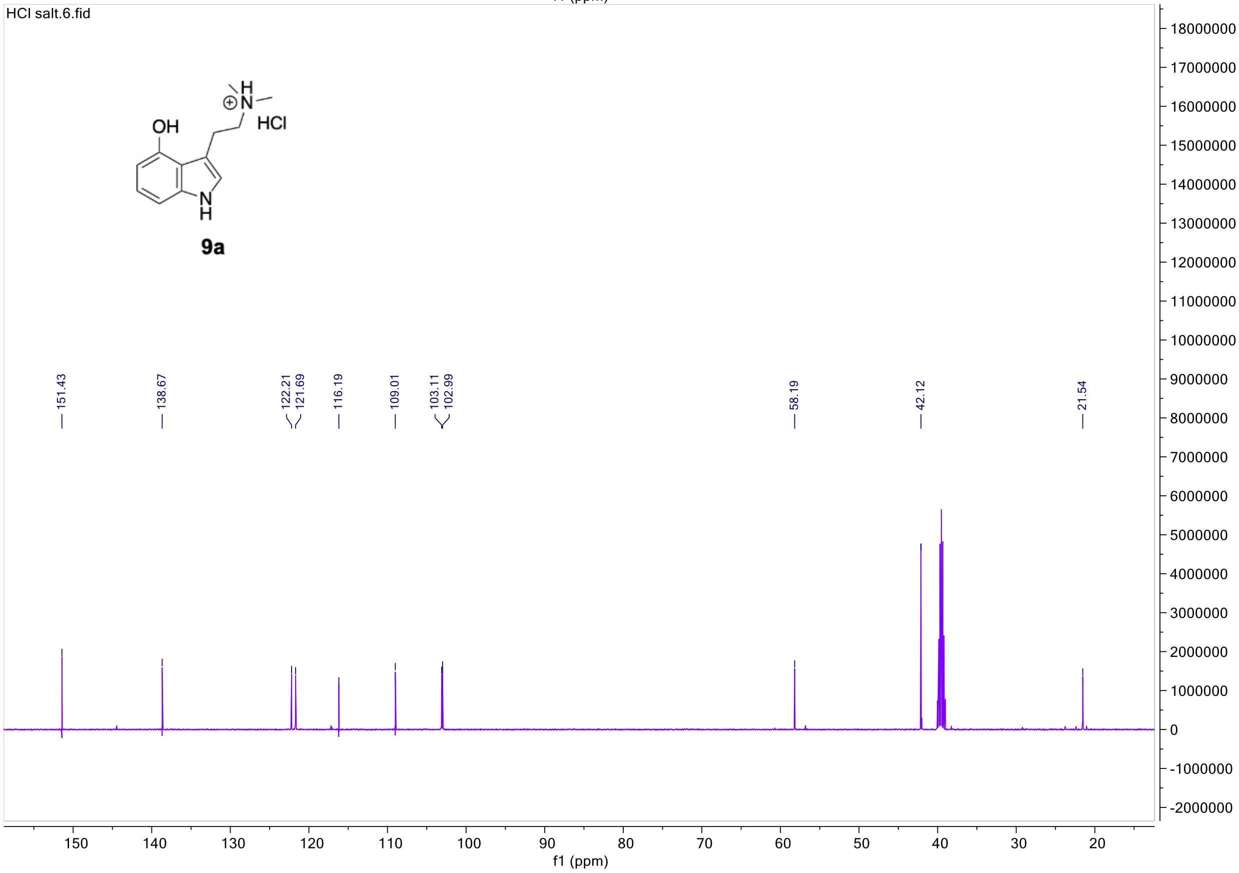
HCl salt.6.fid
OH
H
N
HCl
N
H
9a
151.43
138.67
122.21
121.69
116.19
109.01
103.11
102.99
58.19
42.12
21.54
f1 (ppm)

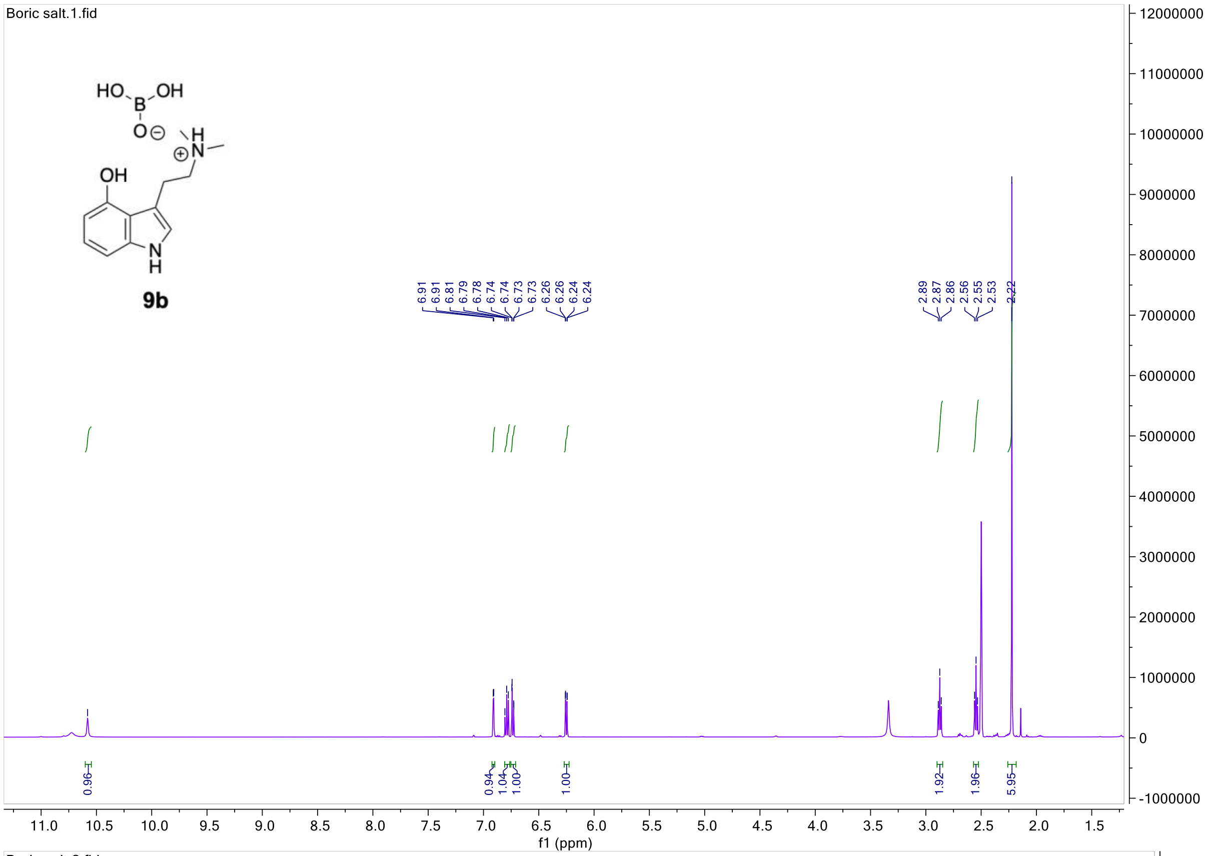
Boric salt.1.fid
HO B OH
O
H
N
OH
N
H
9b
6.91
6.91
6.81
6.79
6.78
6.74
6.74
6.73
6.73
6.26
6.26
6.24
6.24
2.89
2.87
2.86
2.56
2.55
2.53
2.22
0.96
0.94
1.04
1.00
1.00
1.92
1.96
5.95
f1 (ppm)

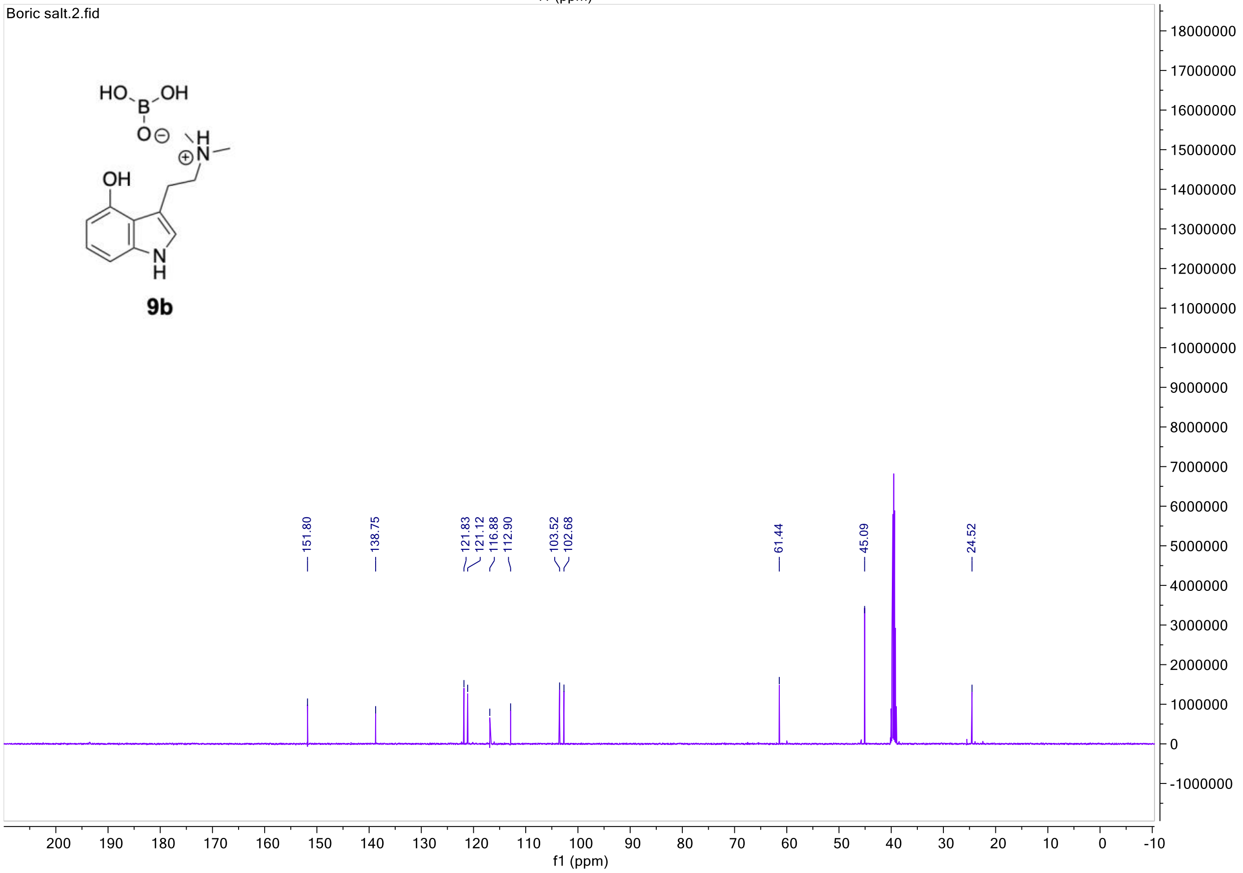
Boric salt.2.fid
HO B OH
O
H
N
OH
N
H
9b
151.80
138.75
121.83
121.12
116.88
112.90
103.52
102.68
61.44
45.09
24.52
f1 (ppm)

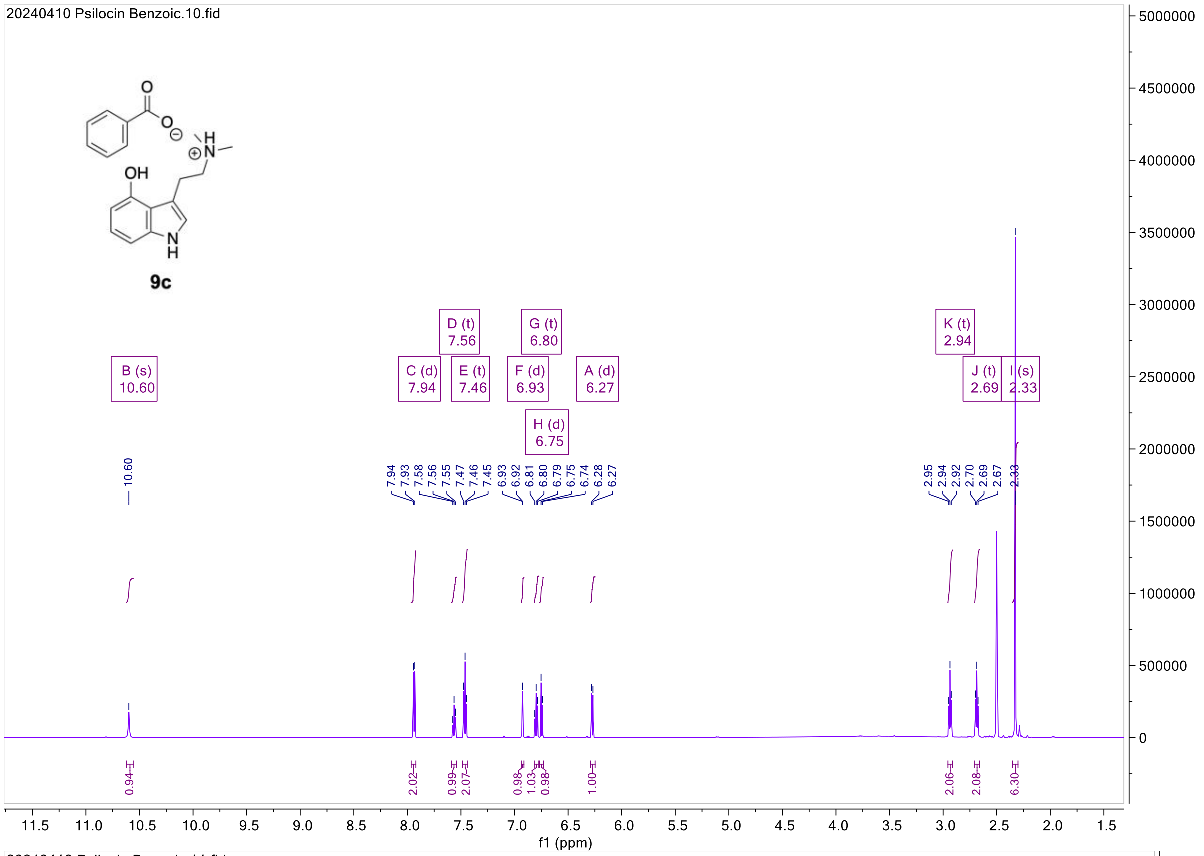
20240410 Psilocin Benzoic.10.fid
9c
B (s) 10.60
C (d) 7.94
D (t) 7.56
E (t) 7.46
F (d) 6.93
G (t) 6.80
H (d) 6.75
A (d) 6.27
K (t) 2.94
J (t) 2.69
I (s) 2.33
f1 (ppm)

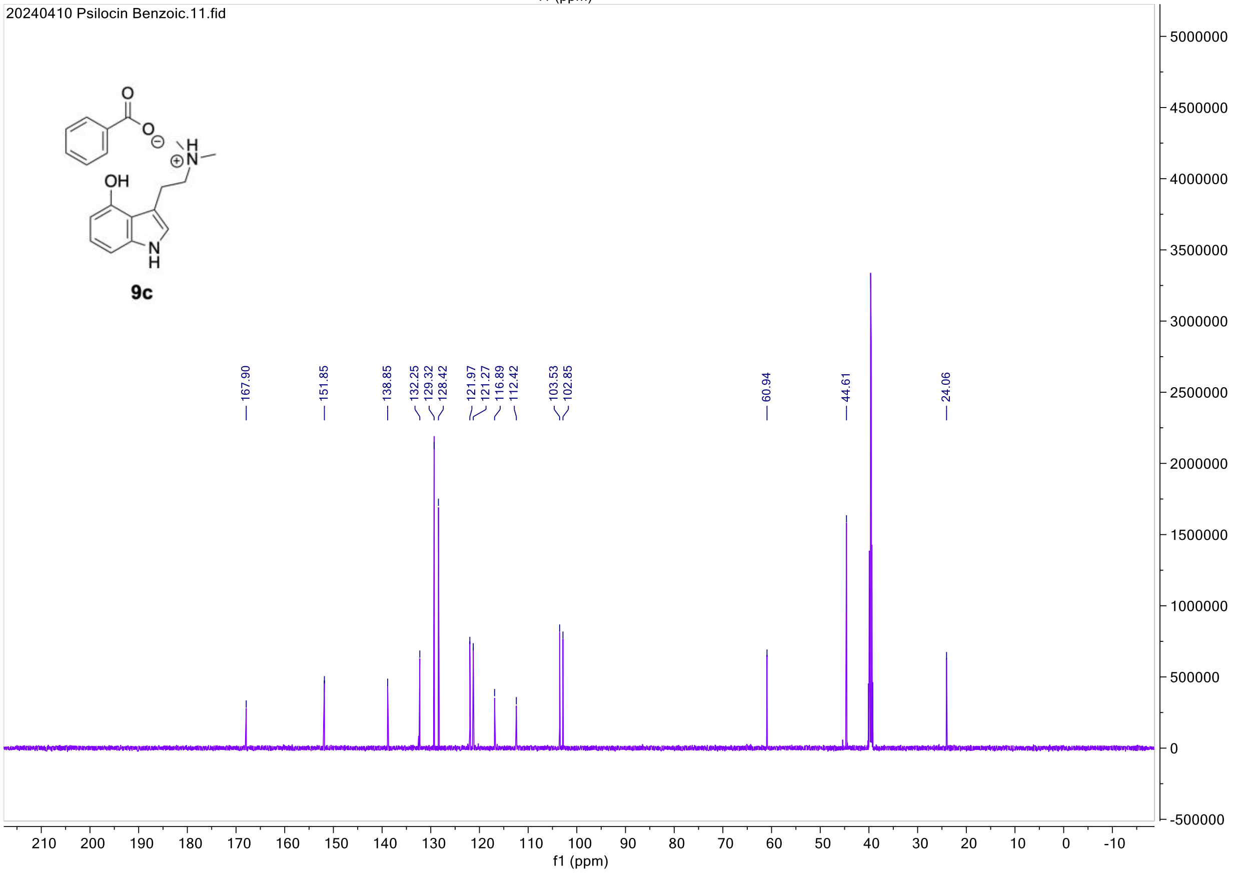
20240410 Psilocin Benzoic.11.fid
9c
167.90
151.85
138.85
132.25
129.32
128.42
121.97
121.27
116.89
112.42
103.53
102.85
60.94
44.61
24.06
f1 (ppm)

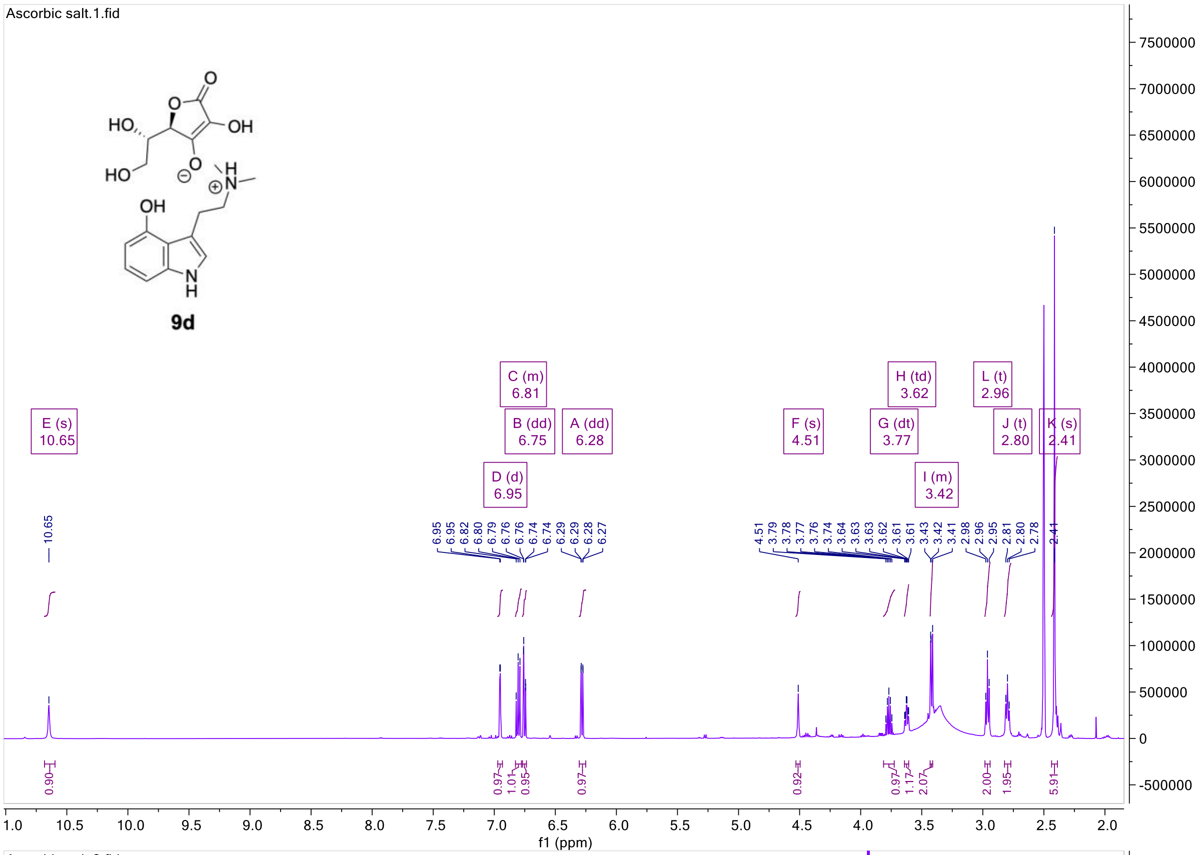
Ascorbic salt.1.fid
9d
E (s) 10.65
C (m) 6.81
B (dd) 6.75
A (dd) 6.28
D (d) 6.95
F (s) 4.51
H (td) 3.62
G (dt) 3.77
I (m) 3.42
L (t) 2.96
J (t) 2.80
K (s) 2.41
10.65
6.95 6.95 6.82 6.80 6.79 6.76 6.76 6.74 6.29 6.29 6.28 6.27
4.51 3.79 3.78 3.77 3.76 3.74 3.64 3.63 3.63 3.62 3.61 3.61 3.43 3.42 3.41 2.98 2.96 2.95 2.81 2.80 2.78 2.41
0.90 0.97 1.01 0.95 0.97 0.92 0.97 1.17 2.07 2.00 1.95 5.91
f1 (ppm)
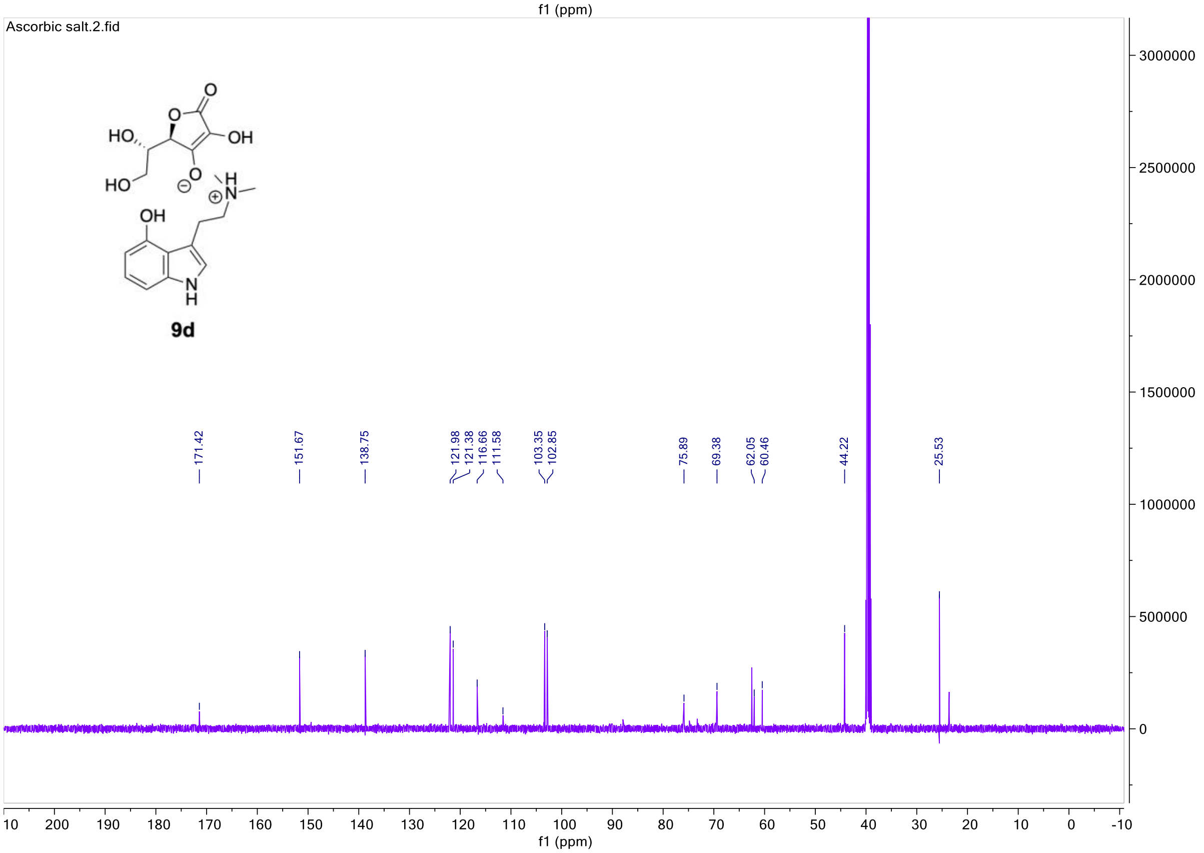
Ascorbic salt.2.fid
9d
171.42
151.67
138.75
121.98 121.38 116.66 111.58
103.35 102.85
75.89
69.38
62.05 60.46
44.22
25.53
f1 (ppm)

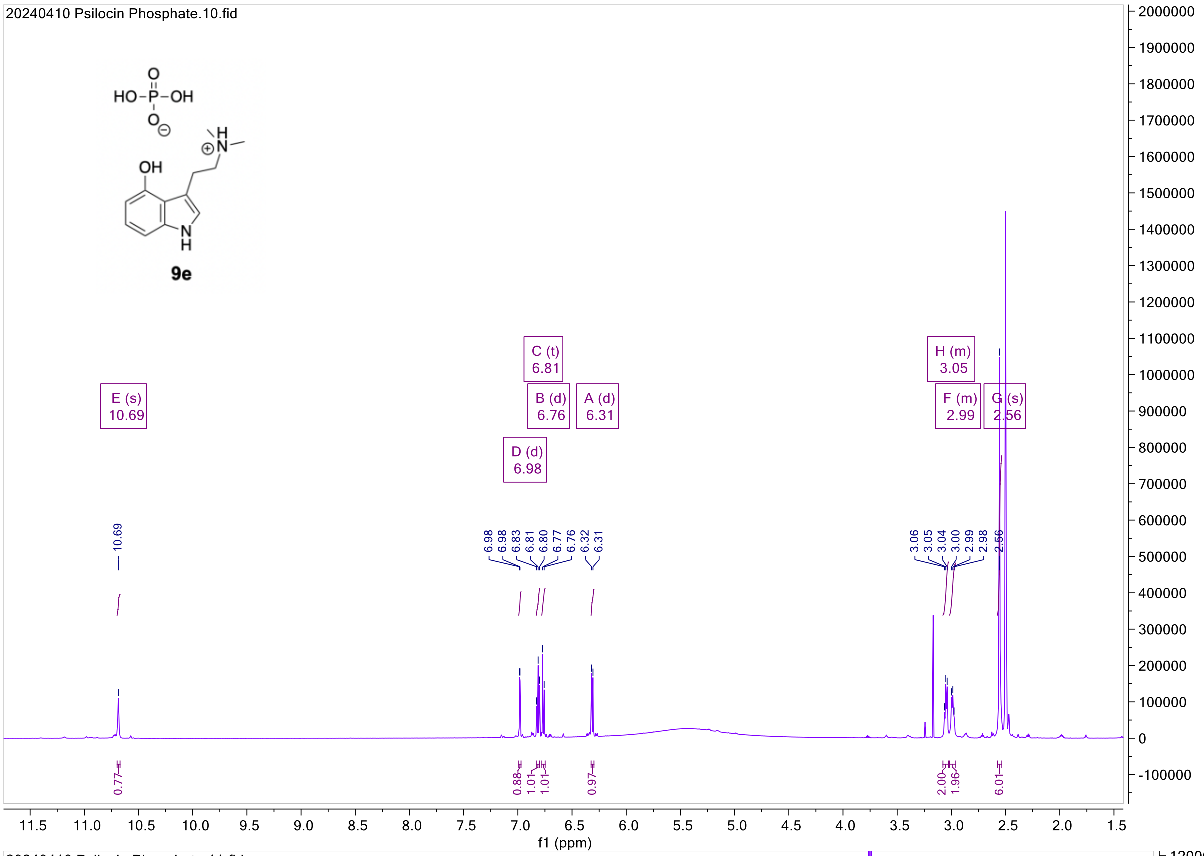

20240410 Psilocin Phosphate.10.fid
9e
E (s) 10.69
C (t) 6.81
B (d) 6.76
A (d) 6.31
D (d) 6.98
H (m) 3.05
F (m) 2.99
G (s) 2.56
f1 (ppm)

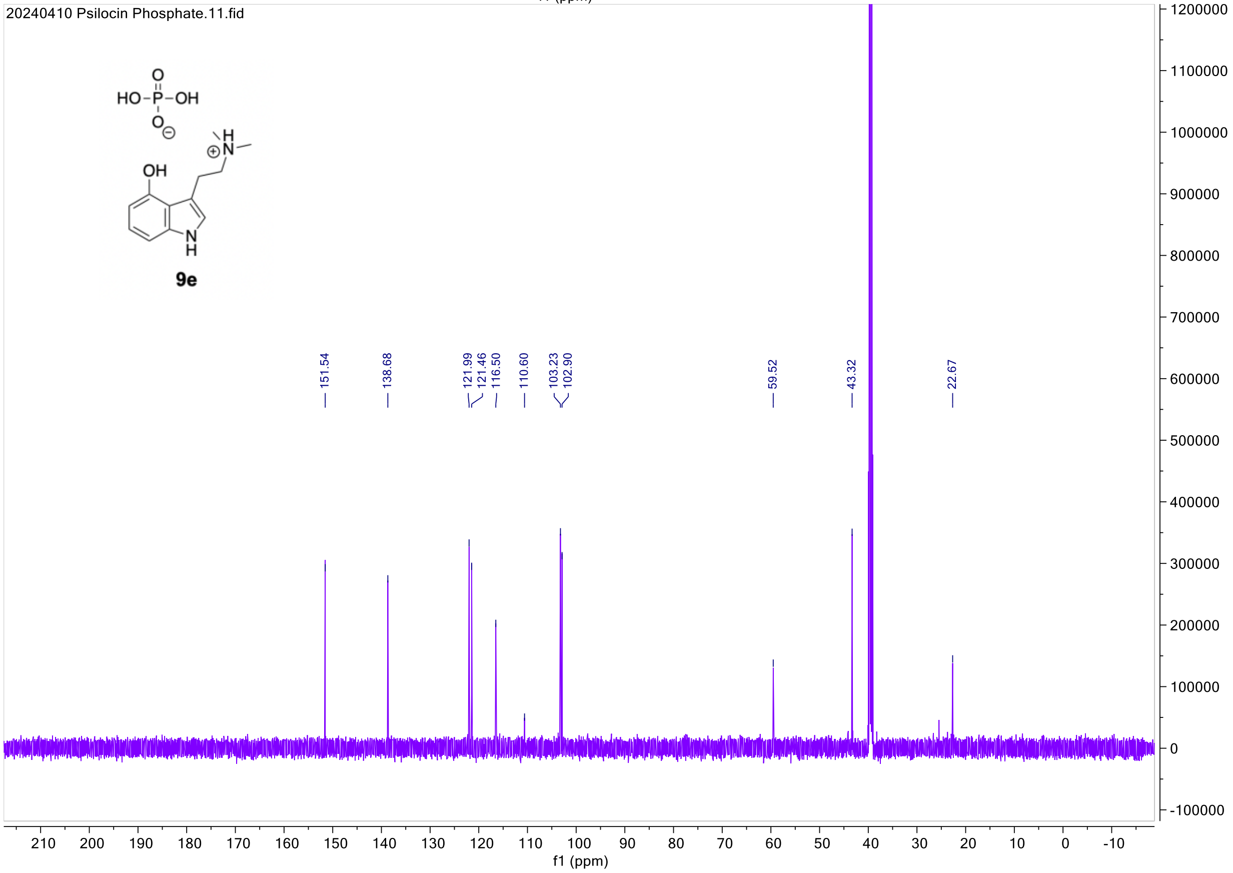

20240410 Psilocin Phosphate.11.fid
9e
151.54
138.68
121.99
121.46
116.50
110.60
103.23
102.90
59.52
43.32
22.67
f1 (ppm)

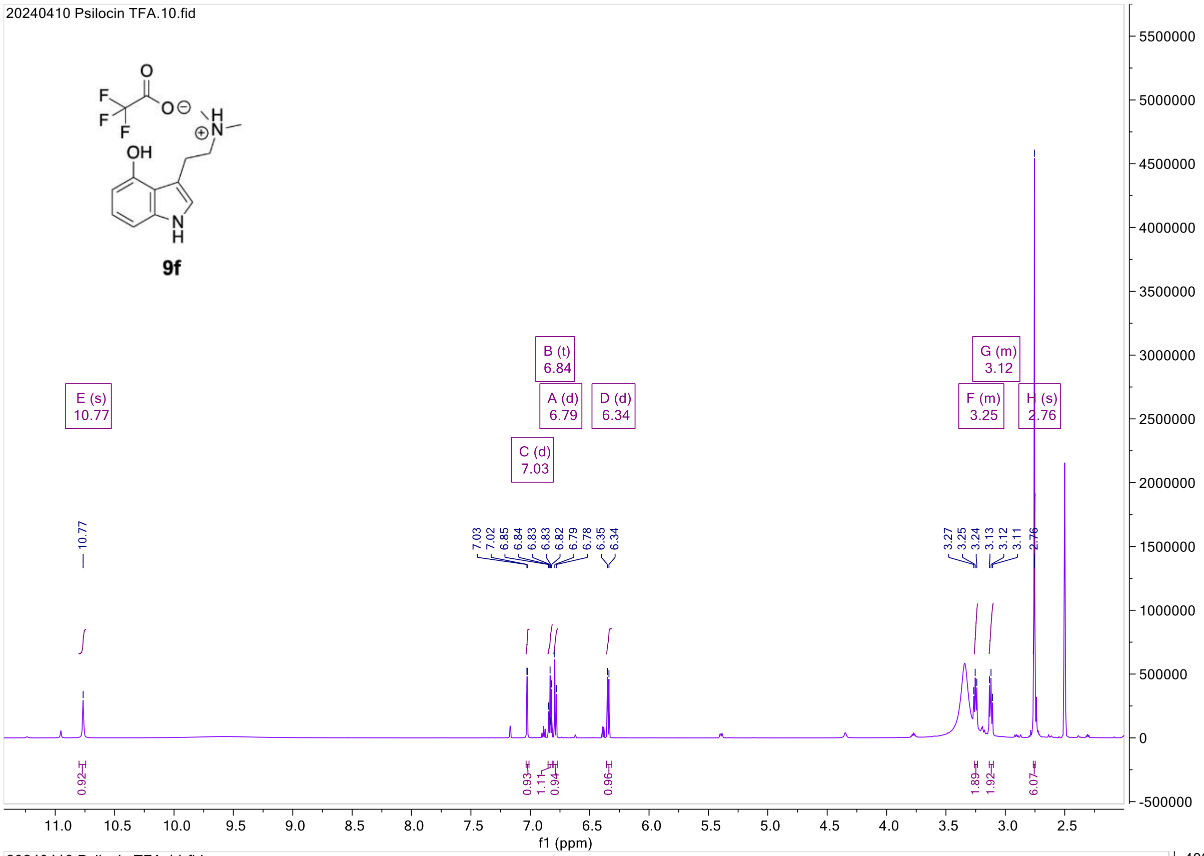
20240410 Psilocin TFA.10.fid
9f
E (s) 10.77
C (d) 7.03
B (t) 6.84
A (d) 6.79
D (d) 6.34
G (m) 3.12
F (m) 3.25
H (s) 2.76
10.77
7.03 7.02 6.85 6.84 6.83 6.83 6.82 6.79 6.78 6.35 6.34
3.27 3.25 3.24 3.13 3.12 3.11 2.76
0.92 0.93 1.11 0.94 0.96 1.89 1.92 6.07
f1 (ppm)

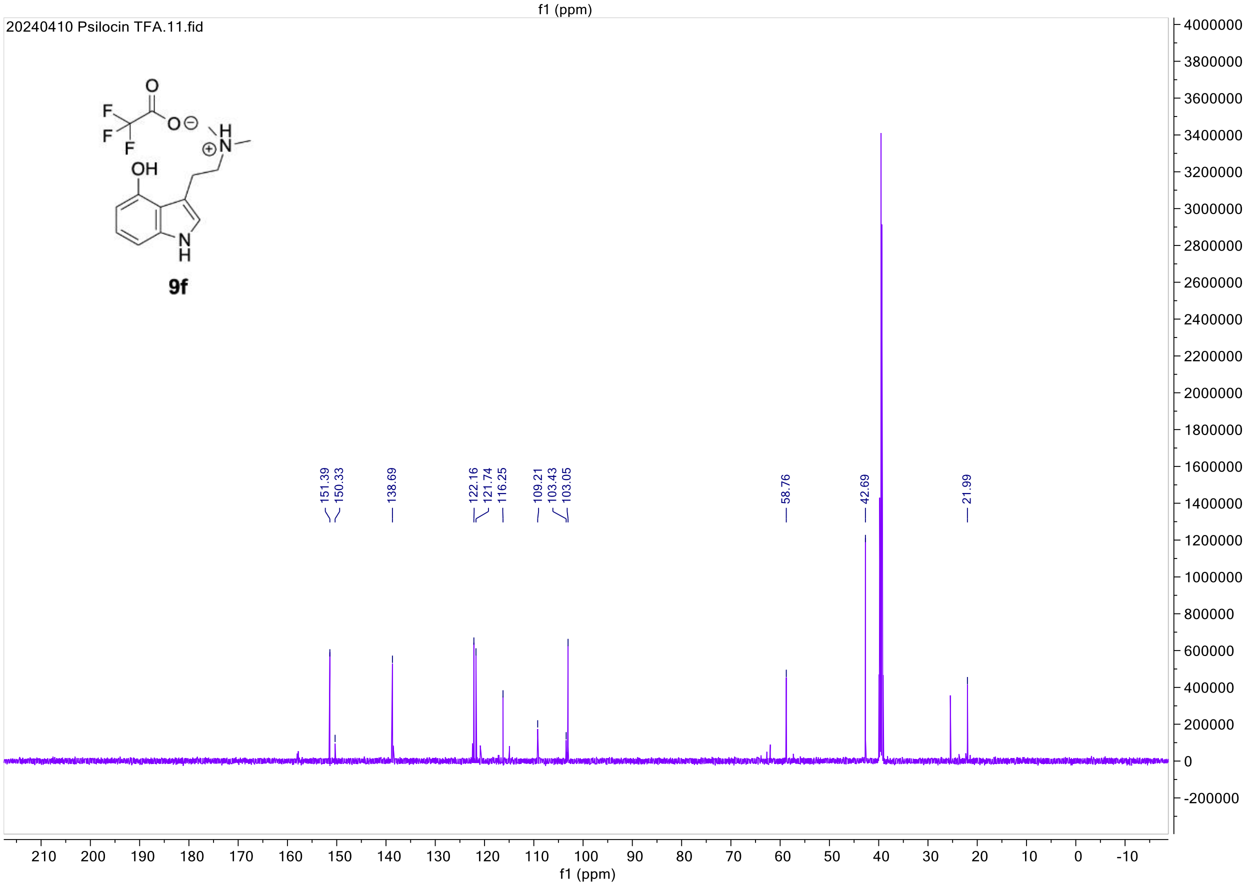
20240410 Psilocin TFA.11.fid
9f
151.39 150.33 138.69 122.16 121.74 116.25 109.21 103.43 103.05 58.76 42.69 21.99
f1 (ppm)

Representative LCMS

9b

[illegible]

**Sample Name:**	Boric salt	**Injection Volume:**	1.0 uL
**Control Program:**	0-40% positive	**UserID**	JuliaE
**Recording Time:**	2/28/2024 13:13		

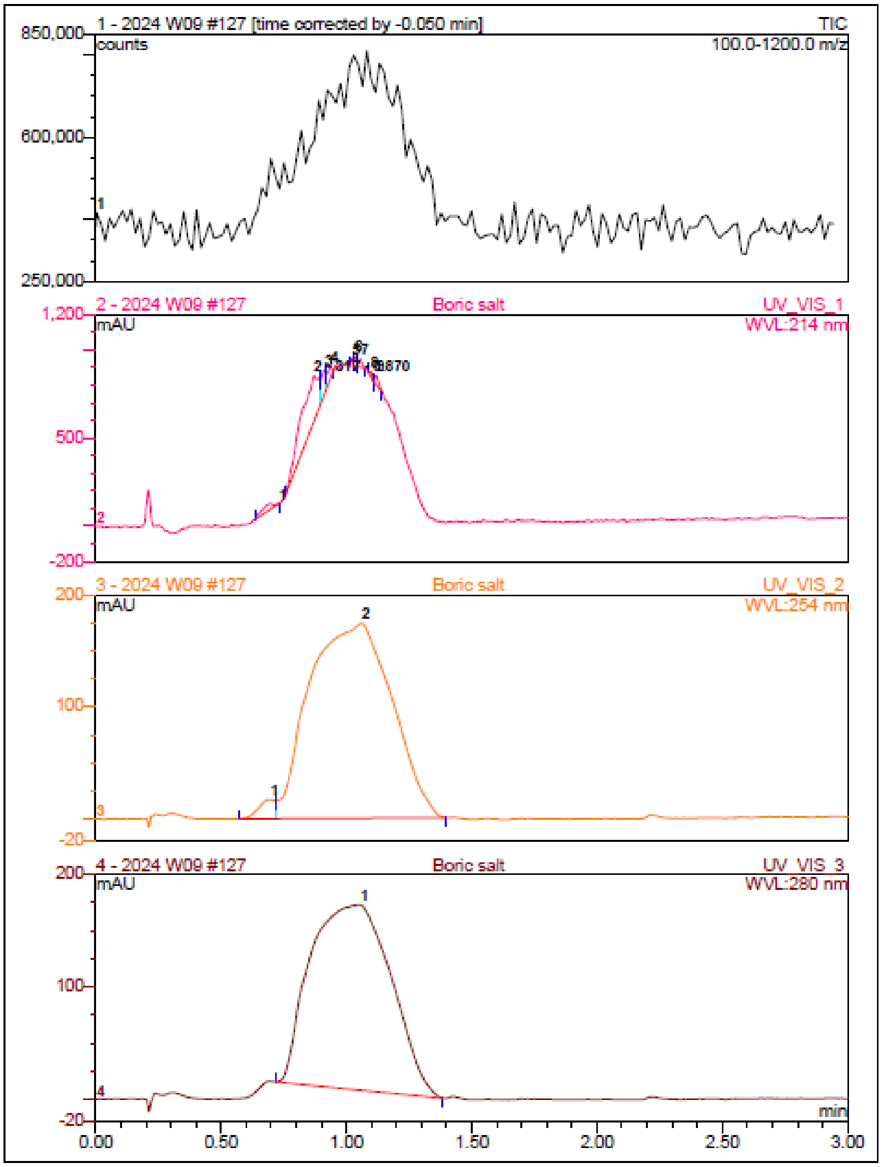

No.	Ret.Time min	Height mAU	Area mAU*min	Rel.Area %	Highest m/z responses Mass - 1 m/z	Mass -2 m/z	Mass - 3 m/z
1	0.70	16.903	1.251	1.86	203	204	188
2	1.06	173.619	66.160	98.14	160	205	207

9c

**Sample Name:**	Benzoic salt	**Injection Volume:**	1.0 uL
**Control Program:**	0-40% positive	**UserID**	JuliaE
**Recording Time:**	2/28/2024 13:18		

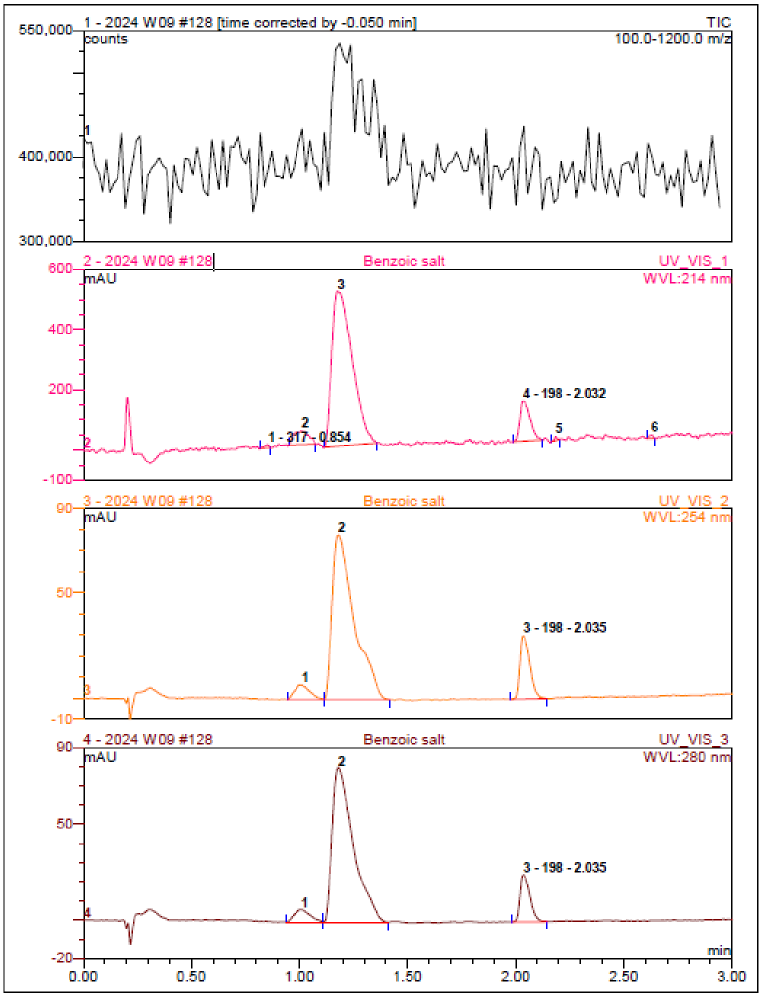

No.	Ret.Time	Height	Area	Rel.Area	Mass - 1	Mass -2	Mass - 3
	min	mAU	mAU*min	%	m/z	m/z	m/z
1	1.01	6.818	0.559	4.93	203	204	130
2	1.18	78.096	9.178	80.97	205	160	206
3	2.04	29.935	1.598	14.10	104	141	183

Highest m/z responses

9d

E-240204 vial 1 Cru

**Sample Name:**	Ascorbic salt	**Injection Volume:**	1.0 uL
**Control Program:**	0-40% positive	**UserID**	JuliaE
**Recording Time:**	2/28/2024 13:23		

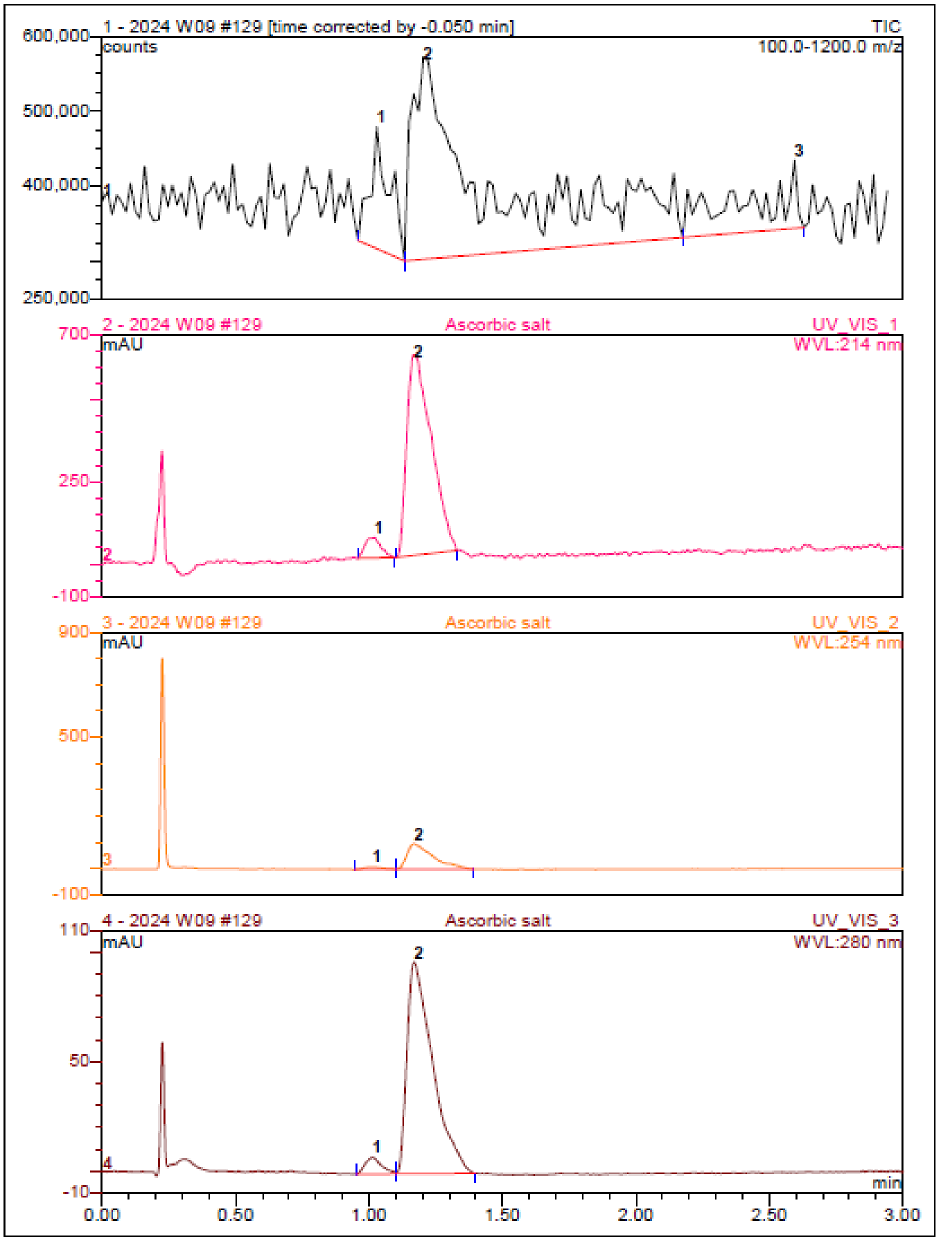

No.	Ret.Time min	Height mAU	Area mAU*min	Rel.Area %	Mass - 1 m/z	Mass -2 m/z	Mass - 3 m/z
					Highest m/z responses		
1	1.01	8.189	0.598	5.28	203	204	103
2	1.17	94.889	10.744	94.72	205	160	206

4a

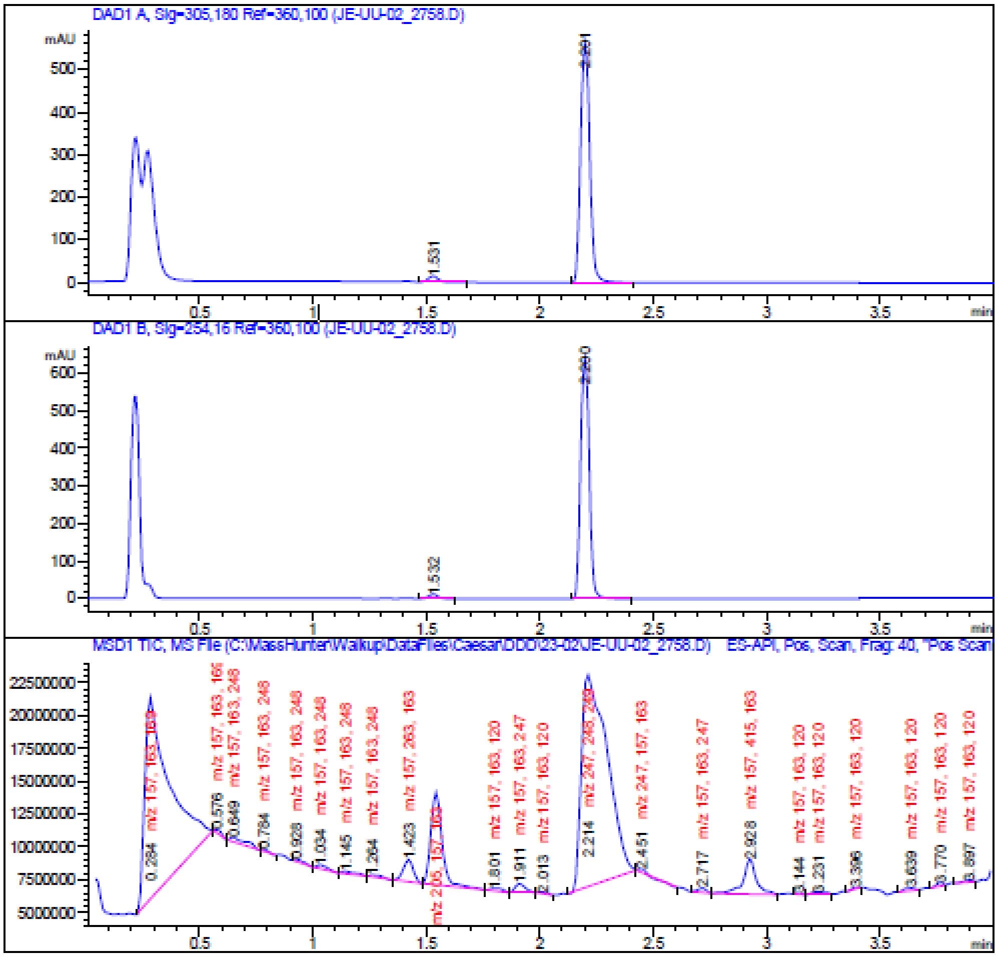
DAD1 A, Sig=305,180 Ref=360,100 (JE-UU-02_2758.D)
DAD1 B, Sig=254,16 Ref=360,100 (JE-UU-02_2758.D)
MSD1 TIC, MS File (C:\MassHunter\Walkup\DataFiles\Caesar\DDD\23-02\JE-UU-02_2758.D) ES-API, Pos, Scan, Frag: 40, "Pos Scan
mAU
min

4h

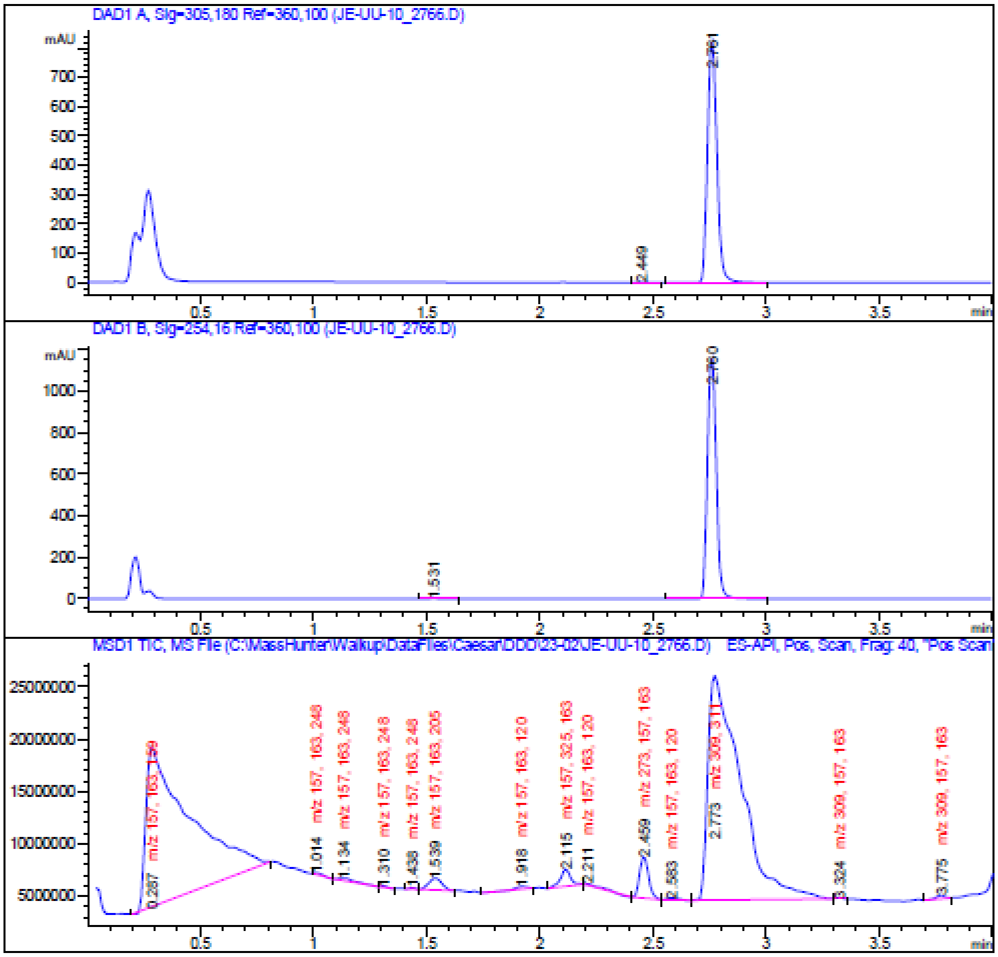
DAD1 A, Sig=305,180 Ref=360,100 (JE-UU-10_2766.D)
DAD1 B, Sig=254,16 Ref=360,100 (JE-UU-10_2766.D)
MSD1 TIC, MS File (C:\MassHunter\Walkup\DataFiles\Caesar\DDD\23-02\JE-UU-10_2766.D) ES-API, Pos, Scan, Frag: 40, "Pos Scan
mAU
min

4k

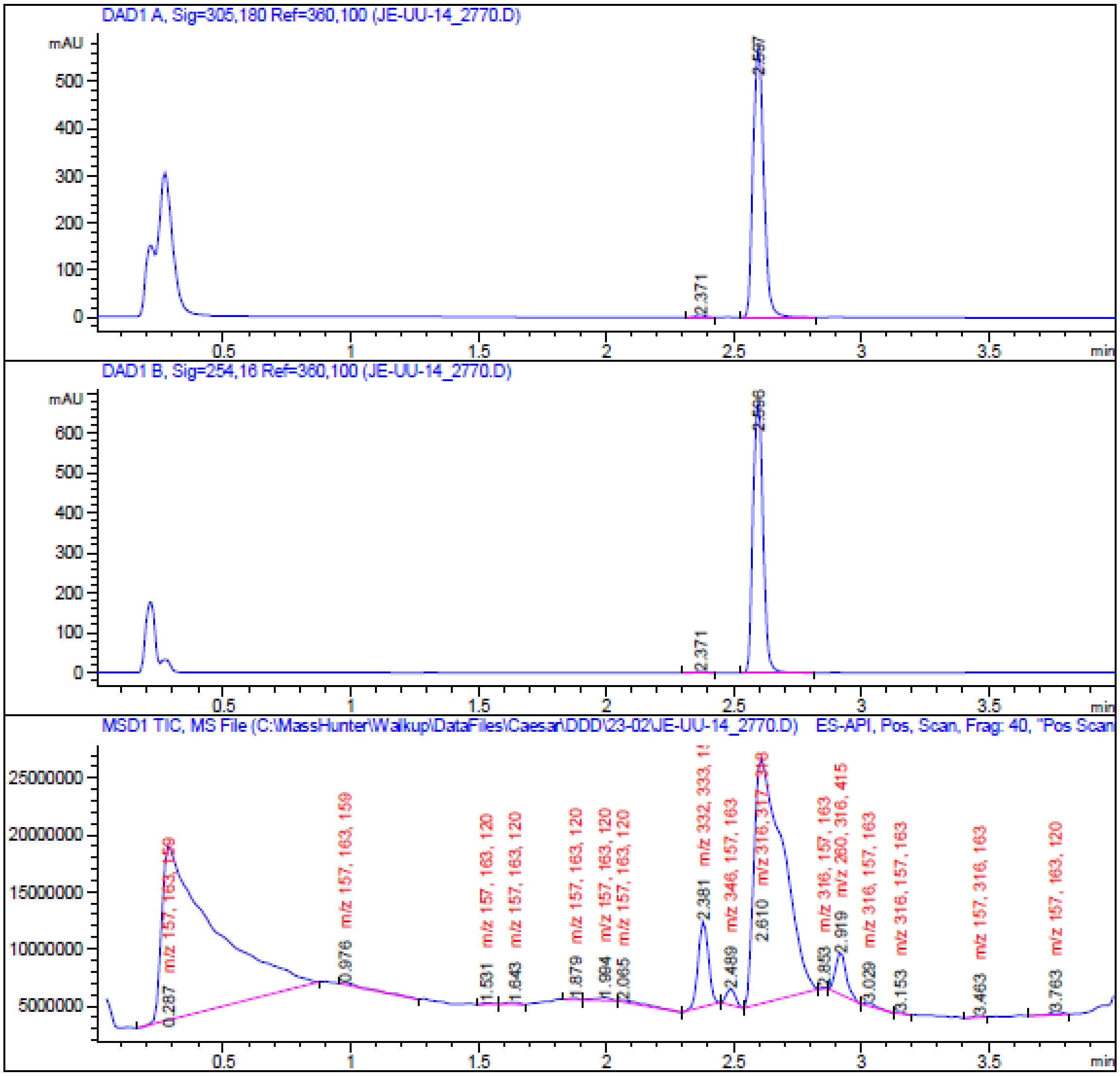
DAD1 A, Sig=305,180 Ref=360,100 (JE-UU-14_2770.D)
mAU
2.371
2.597
DAD1 B, Sig=254,16 Ref=360,100 (JE-UU-14_2770.D)
2.371
2.596
MSD1 TIC, MS File (C:\MassHunter\Walkup\DataFiles\Caesar\DDD\23-02\JE-UU-14_2770.D) ES-API, Pos, Scan, Frag: 40, "Pos Scan
0.287 m/z 157, 163, 159
0.976 m/z 157, 163, 159
1.531 m/z 157, 163, 120
1.643 m/z 157, 163, 120
1.879 m/z 157, 163, 120
1.994 m/z 157, 163, 120
2.065 m/z 157, 163, 120
2.381
2.489 m/z 346, 157, 163
2.610 m/z 316, 317
2.853 m/z 316, 157, 163
2.919 m/z 260, 316, 415
3.029 m/z 316, 157, 163
3.153 m/z 316, 157, 163
3.463 m/z 157, 316, 163
3.763 m/z 157, 163, 120
min

References

(1) Vinarov, Z.; Abdallah, M.; Agundez, J. A. G.; Allegaert, K.; Basit, A. W.; Braeckmans, M.; Ceulemans, J.; Corsetti, M.; Griffin, B. T.; Grimm, M.; Keszthelyi, D.; Koziolek, M.; Madla, C. M.; Matthys, C.; McCoubrey, L. E.; Mitra, A.; Reppas, C.; Stappaerts, J.; Steenackers, N.; Trevaskis, N. L.; Vanuytsel, T.; Vertzoni, M.; Weitschies, W.; Wilson, C.; Augustijns, P. Impact of Gastrointestinal Tract Variability on Oral Drug Absorption and Pharmacokinetics: An UNGAP Review. *European Journal of Pharmaceutical Sciences* 2021, *162*. https://doi.org/10.1016/j.ejps.2021.105812.

(2) Biorelevant. *FaSSIF/FeSSIF/FaSSGF*. https://biorelevant.com/fassif-fessif-fassgf/buy/ (accessed 2024-09-10).

(3) Riethorst, D.; Mols, R.; Duchateau, G.; Tack, J.; Brouwers, J.; Augustijns, P. Characterization of Human Duodenal Fluids in Fasted and Fed State Conditions. *J Pharm Sci* 2016, *105* (2), 673–681. https://doi.org/10.1002/jps.24603.

(4) Kelly, K.; O'mahony, B.; Lindsay, B.; Jones, T.; Grattan, T. J.; Rostami-Hodjegan, A.; Stevens, H. N. E.; Wilson, C. G. Comparison of the Rates of Disintegration, Gastric Emptying, and Drug Absorption Following Administration of a New and a Conventional Paracetamol Formulation, Using Scintigraphy. *Pharm Res* 2003, *20* (10).

(5) Lenz, C.; Wick, J.; Braga, D.; García-Altares, M.; Lackner, G.; Hertweck, C.; Gressler, M.; Hoffmeister, D. Injury-Triggered Blueing Reactions of Psilocybe "Magic" Mushrooms. *Angewandte Chemie International Edition* 2020, *59* (4), 1450–1454.
